# Supplementary figures and images for: mTORC1-induced retinal progenitor cell overproliferation leads to accelerated mitotic aging and degeneration of descendent Müller glia
Source: eLife. 2021 Oct 22;10:e70079. doi: 10.7554/eLife.70079 (PMC8577849; doi:10.7554/eLife.70079)

Fig.5C

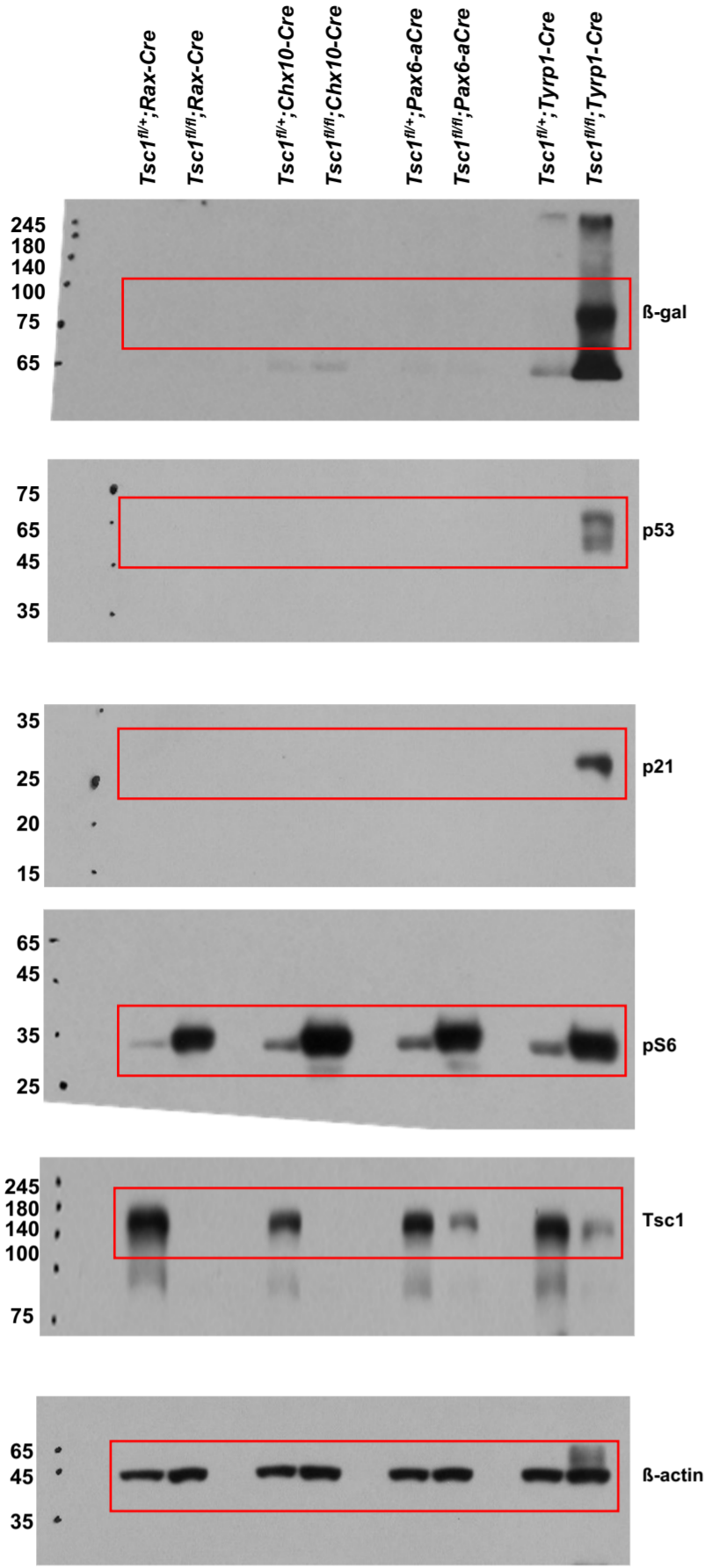

Fig.5I

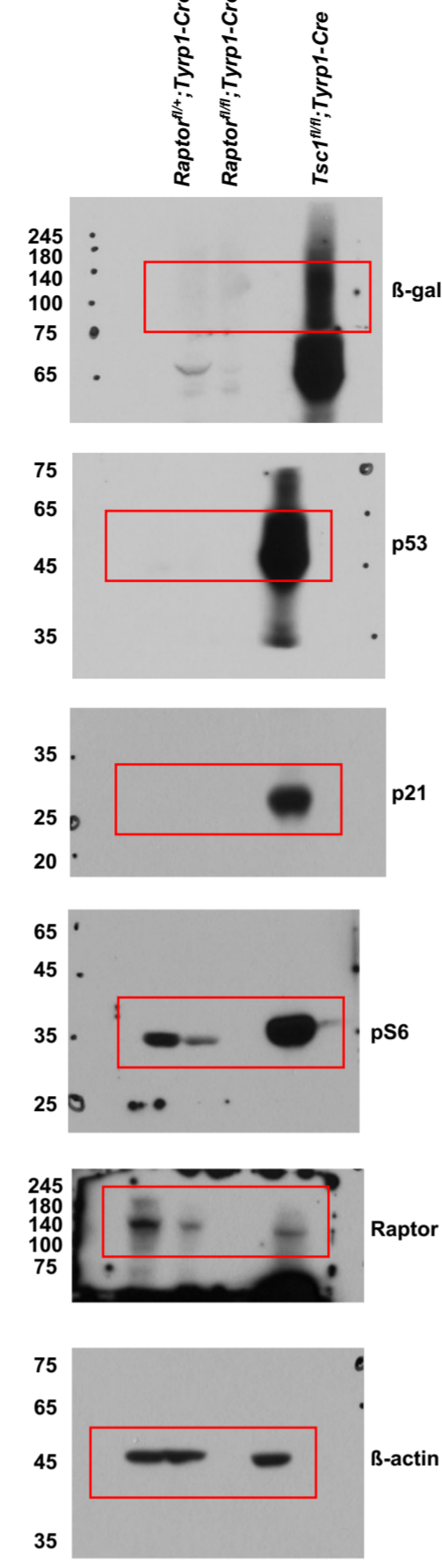

Fig.6A

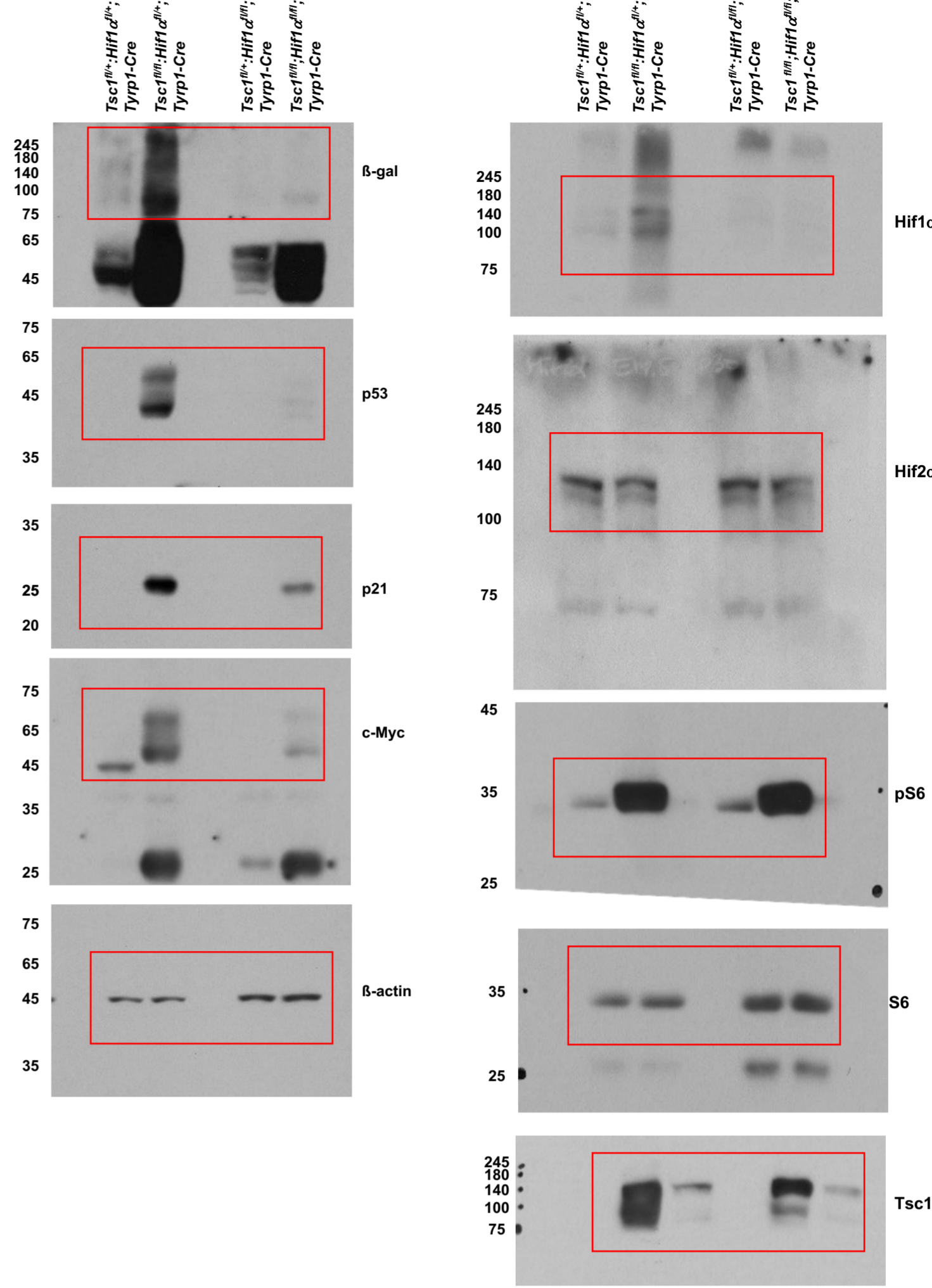

Fig.7E

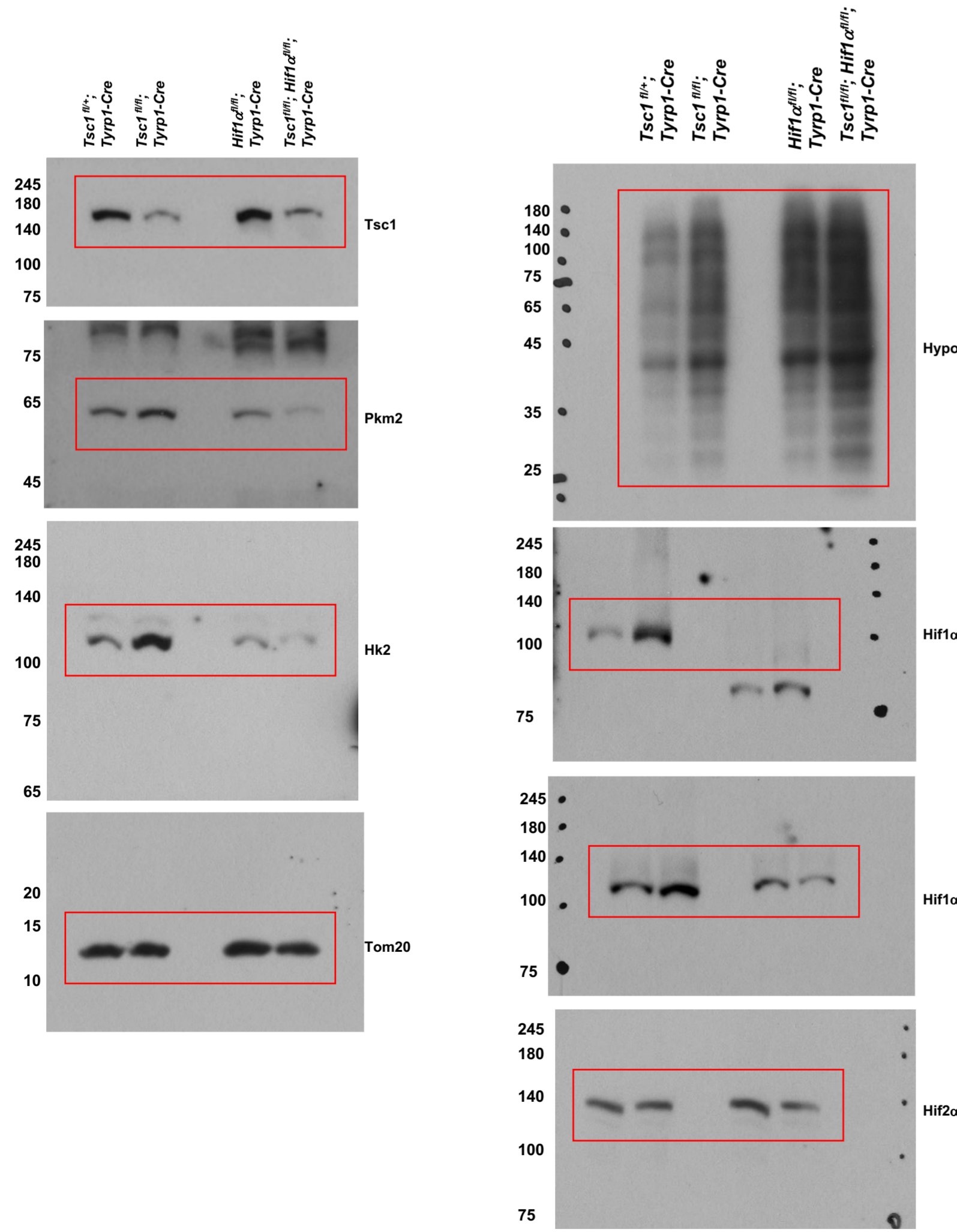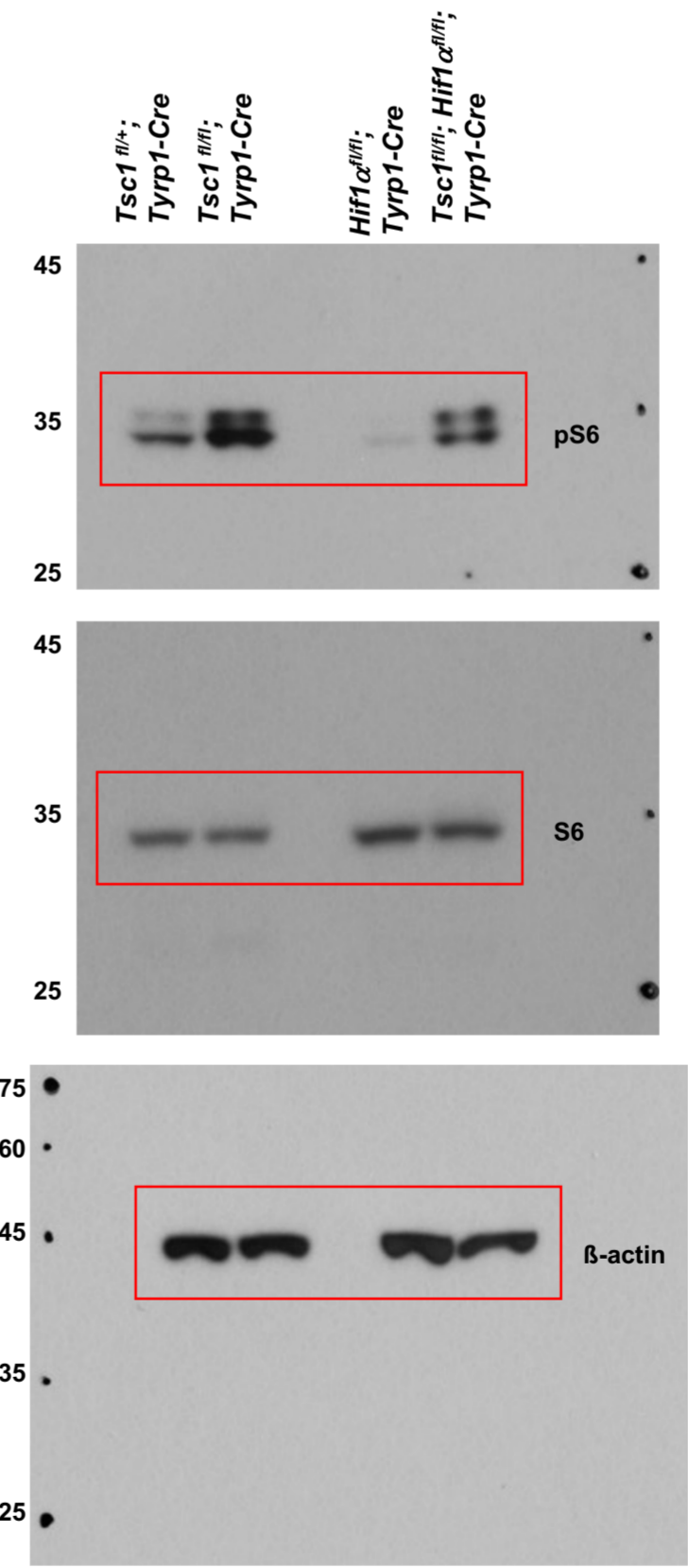

Supplement: Source data 1. [file elife-70079-supp1.pdf]

Fig.1 – fig. suppl. 2B

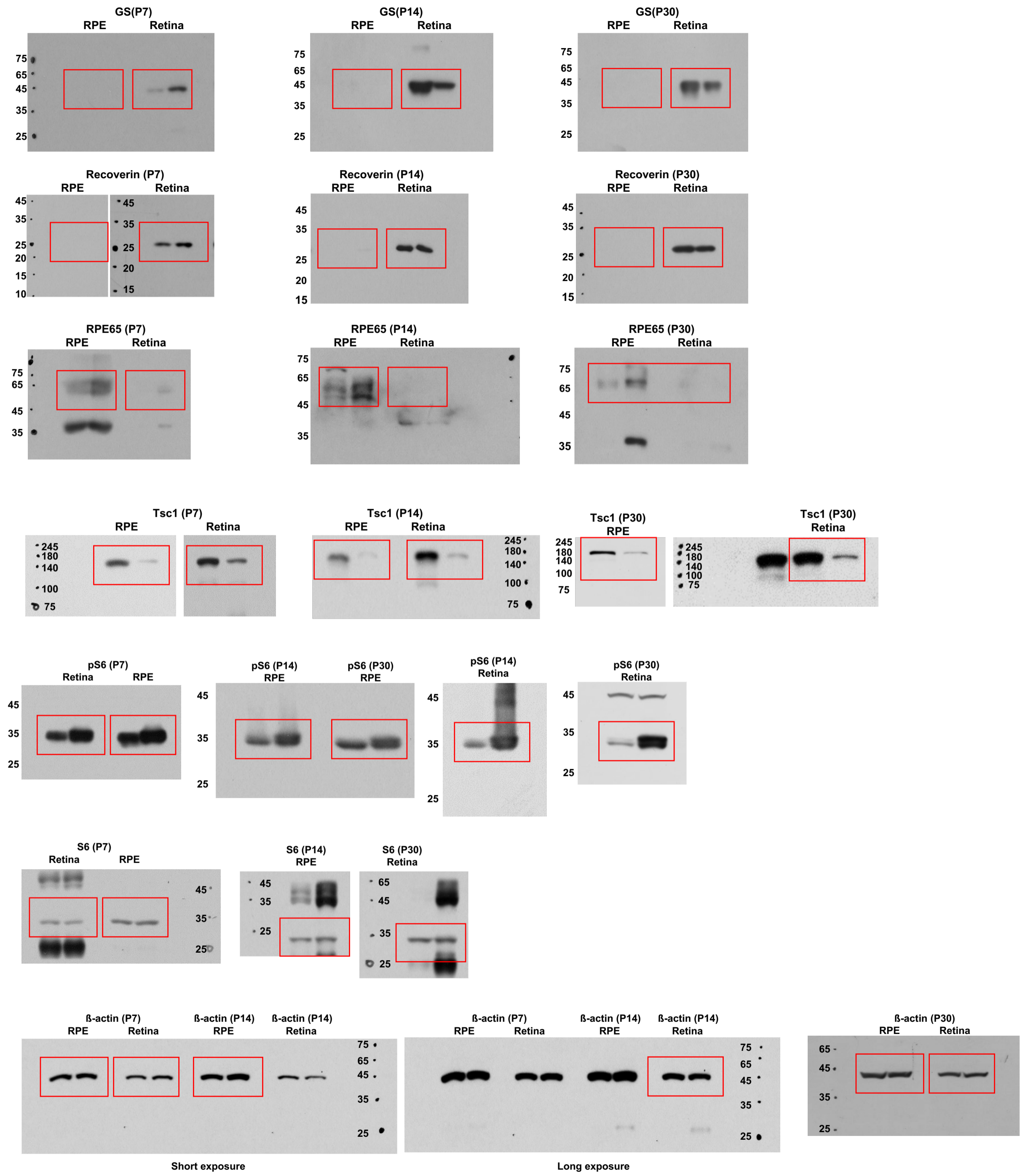

Fig.1 – fig. suppl. 4D

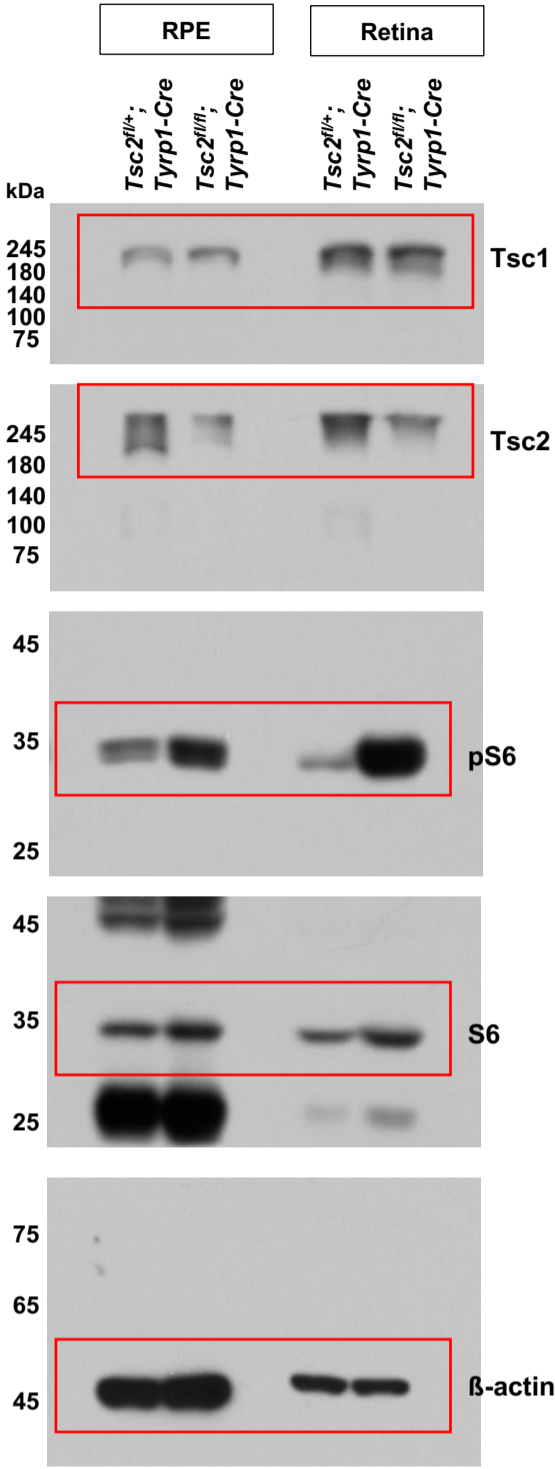

Fig.5 – fig. suppl. 3A

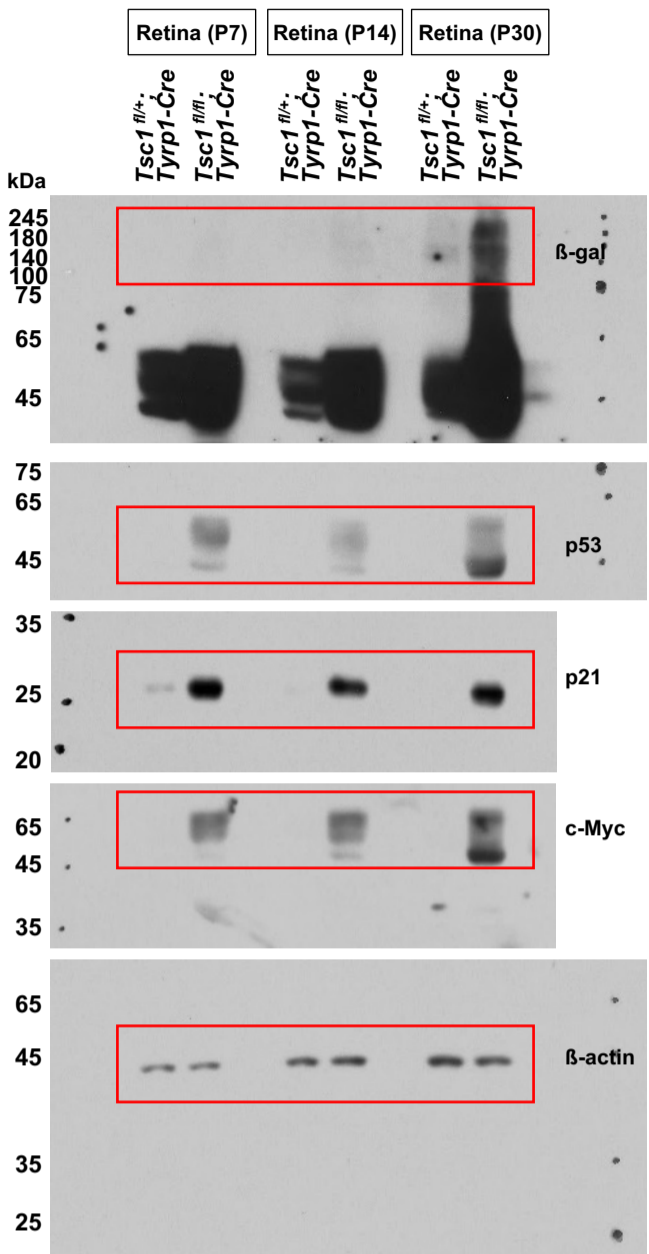

Fig.5 – fig. suppl. 3B

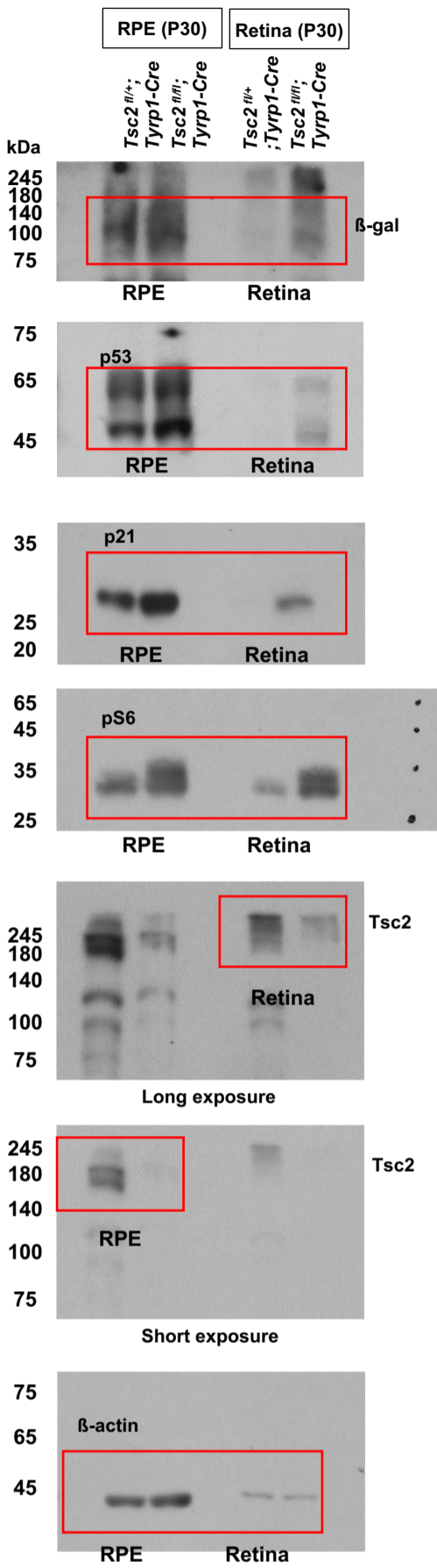

Fig.6 – fig. suppl. 2B

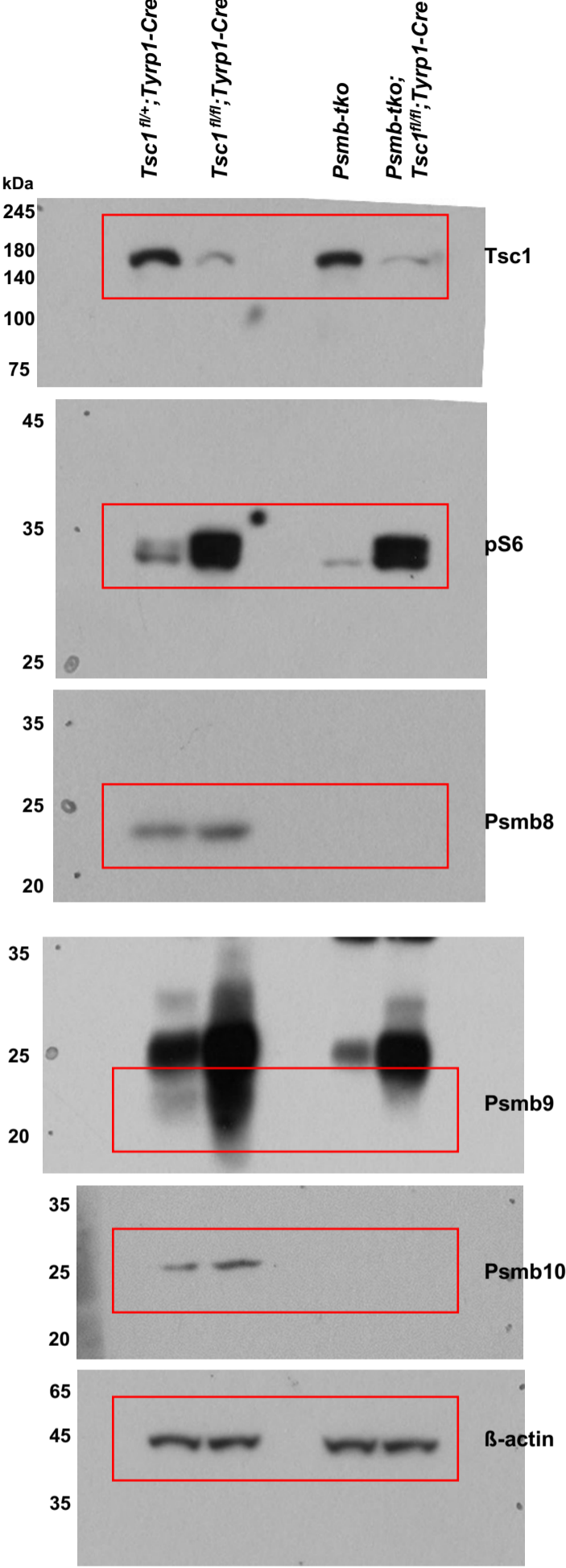

Supplement: Source data 2. [file elife-70079-supp2.pdf]

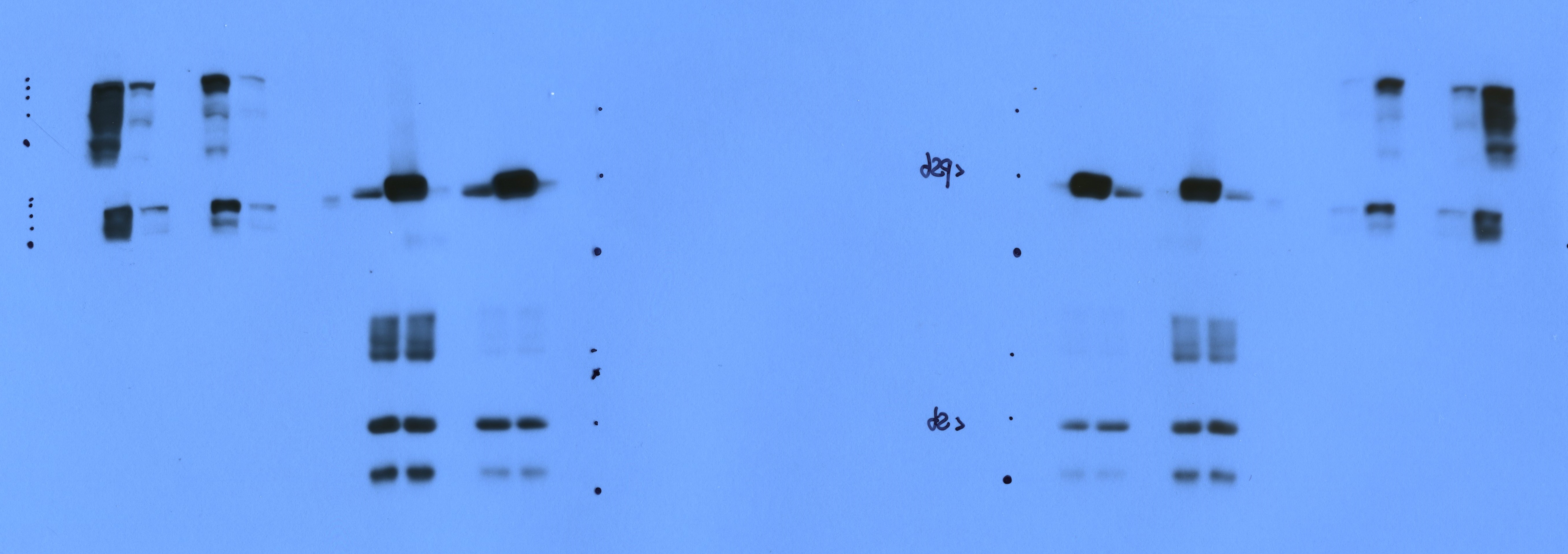

Supplement: Source data 3. [file elife-70079-supp3.zip › Source Data 3_Uncropped WB images for Main Figures/Figure 6A_Tsc1-pS6-S6.jpg]

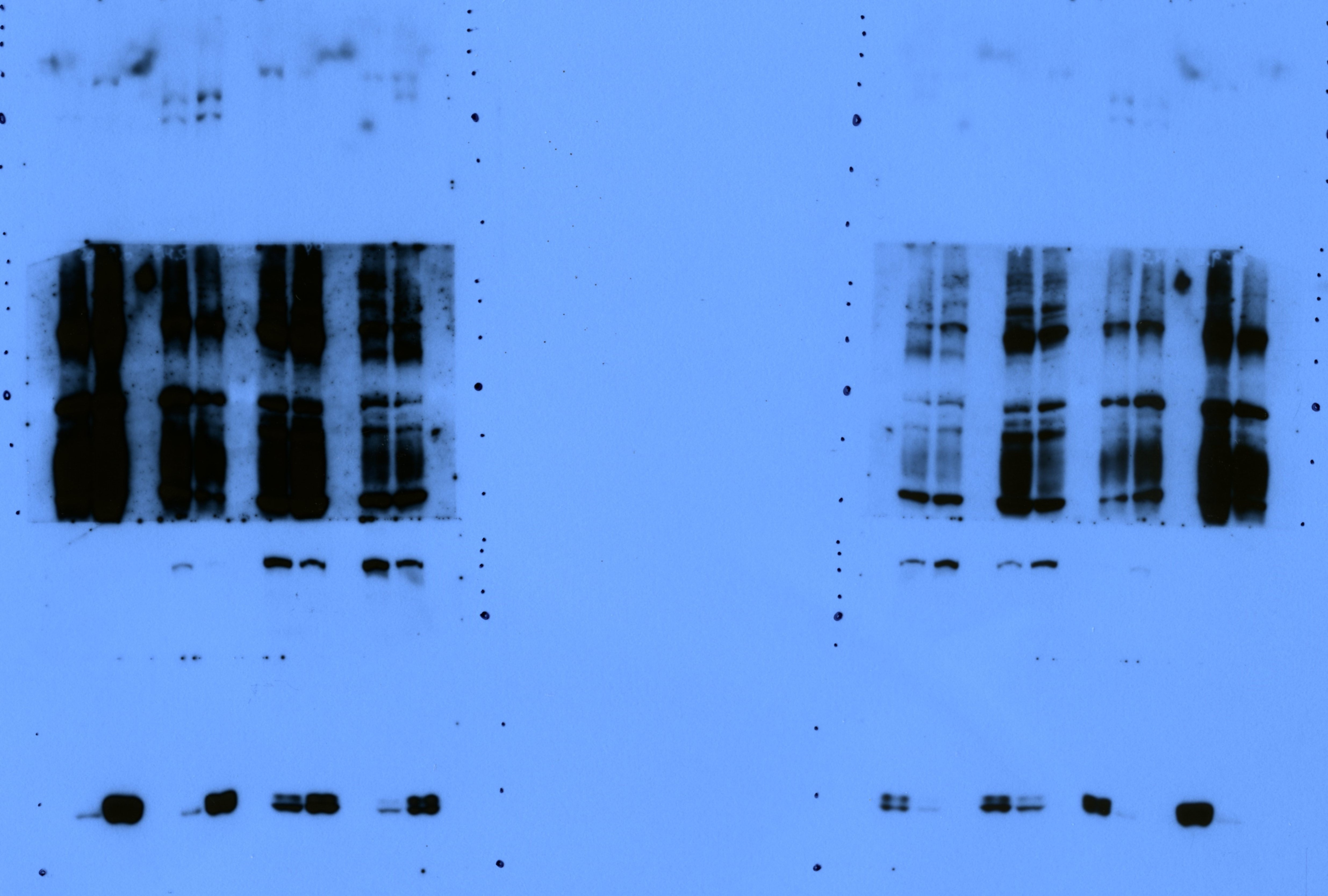

Supplement: Source data 3. [file elife-70079-supp3.zip › Source Data 3_Uncropped WB images for Main Figures/Figure 7E_Tsc1.jpg]

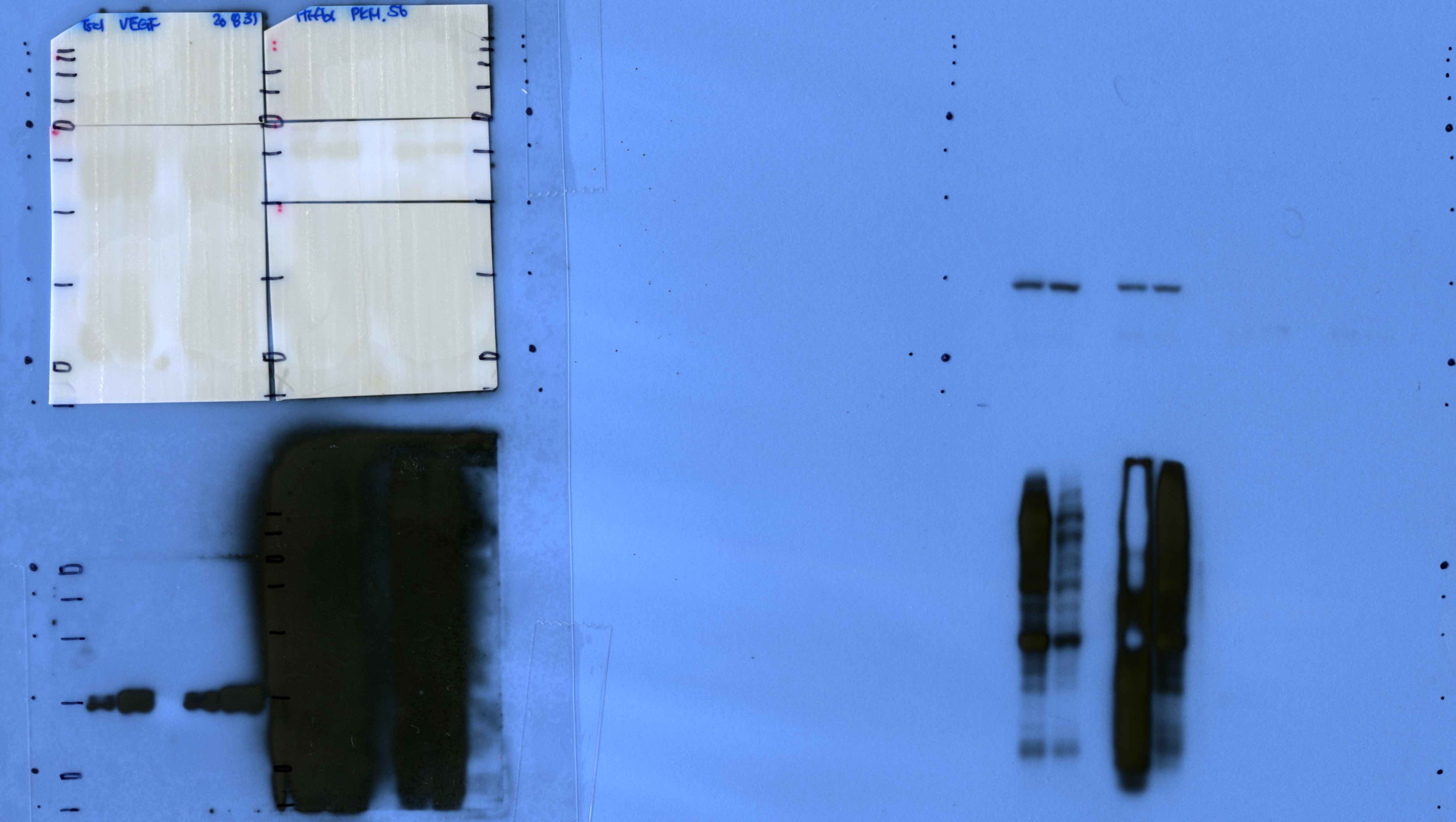

Supplement: Source data 3. [file elife-70079-supp3.zip › Source Data 3_Uncropped WB images for Main Figures/Figure 7E_S6.jpg]

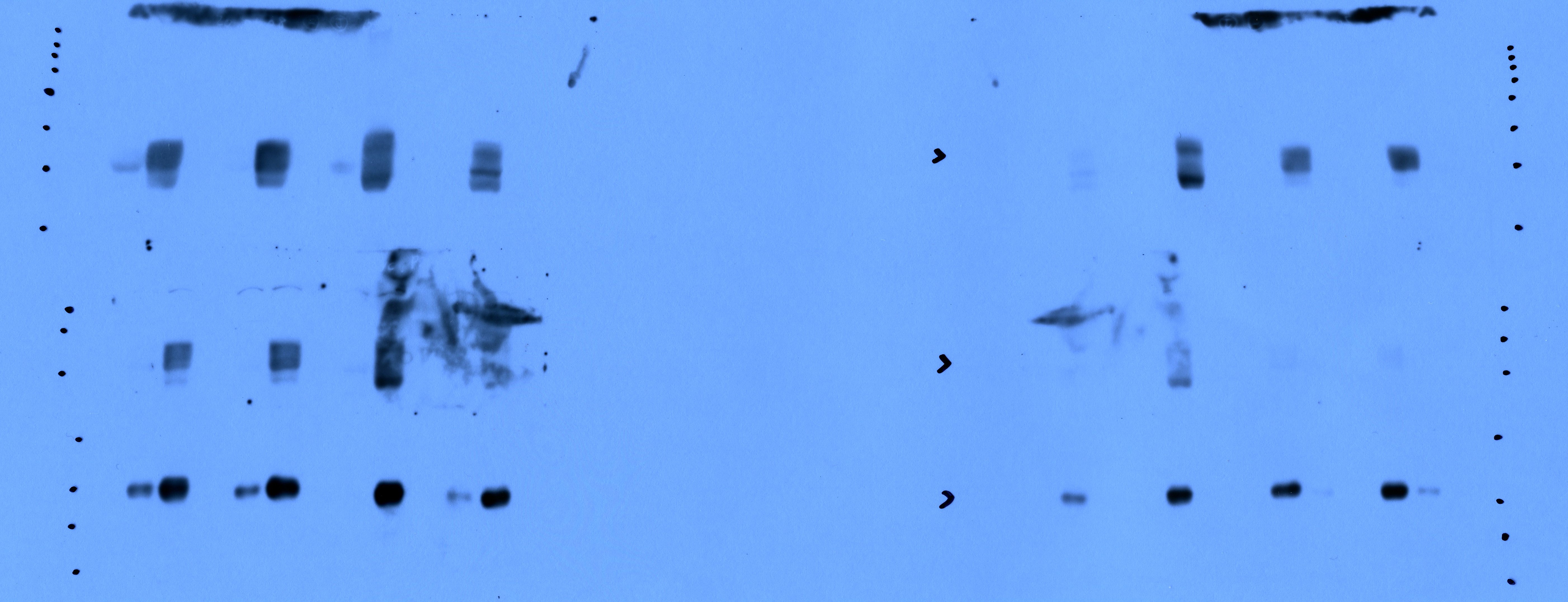

Supplement: Source data 3. [file elife-70079-supp3.zip › Source Data 3_Uncropped WB images for Main Figures/Figure 6A_p53,p21.jpg]

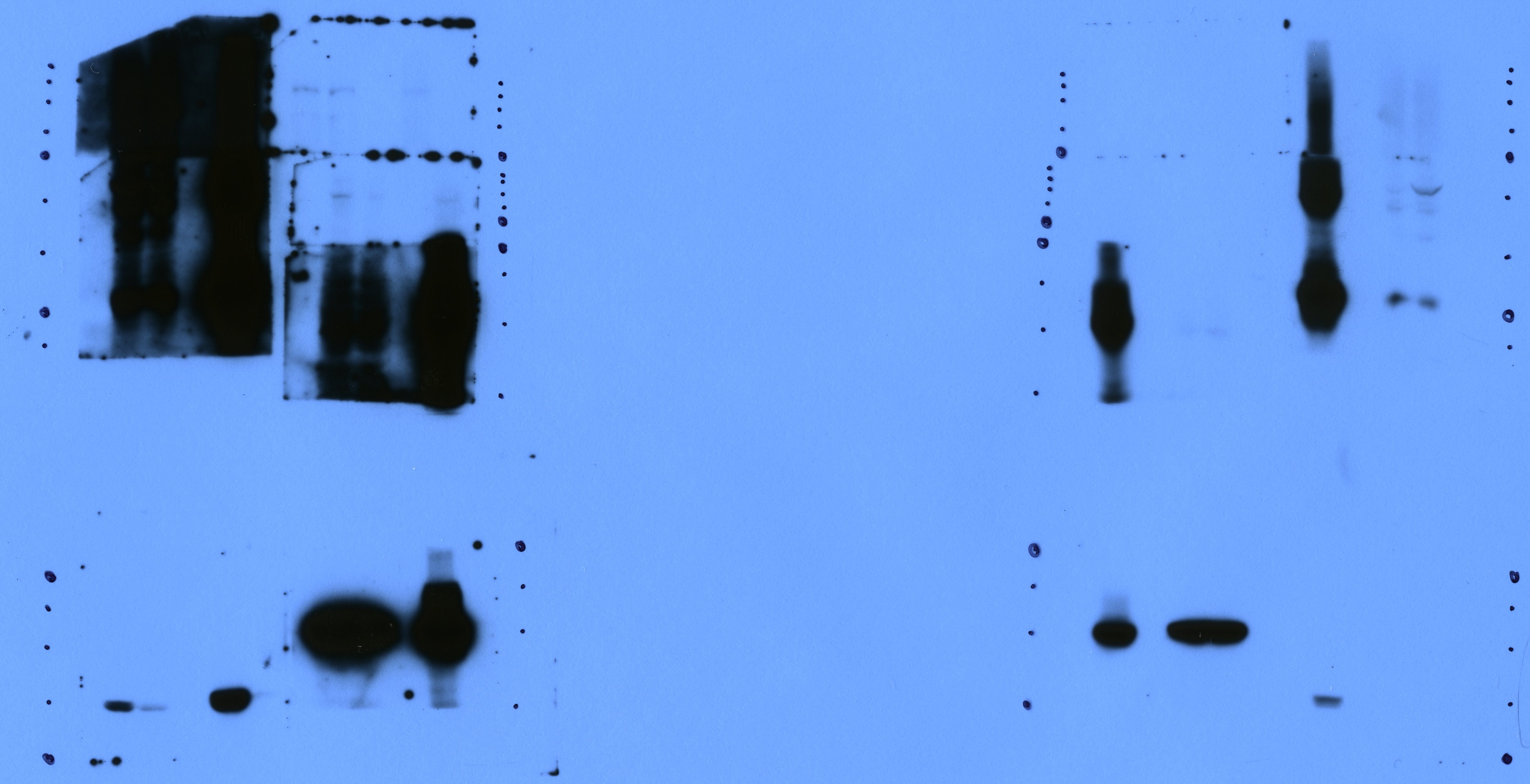

Supplement: Source data 3. [file elife-70079-supp3.zip › Source Data 3_Uncropped WB images for Main Figures/Figure 5I_bataGal-pS6.jpg]

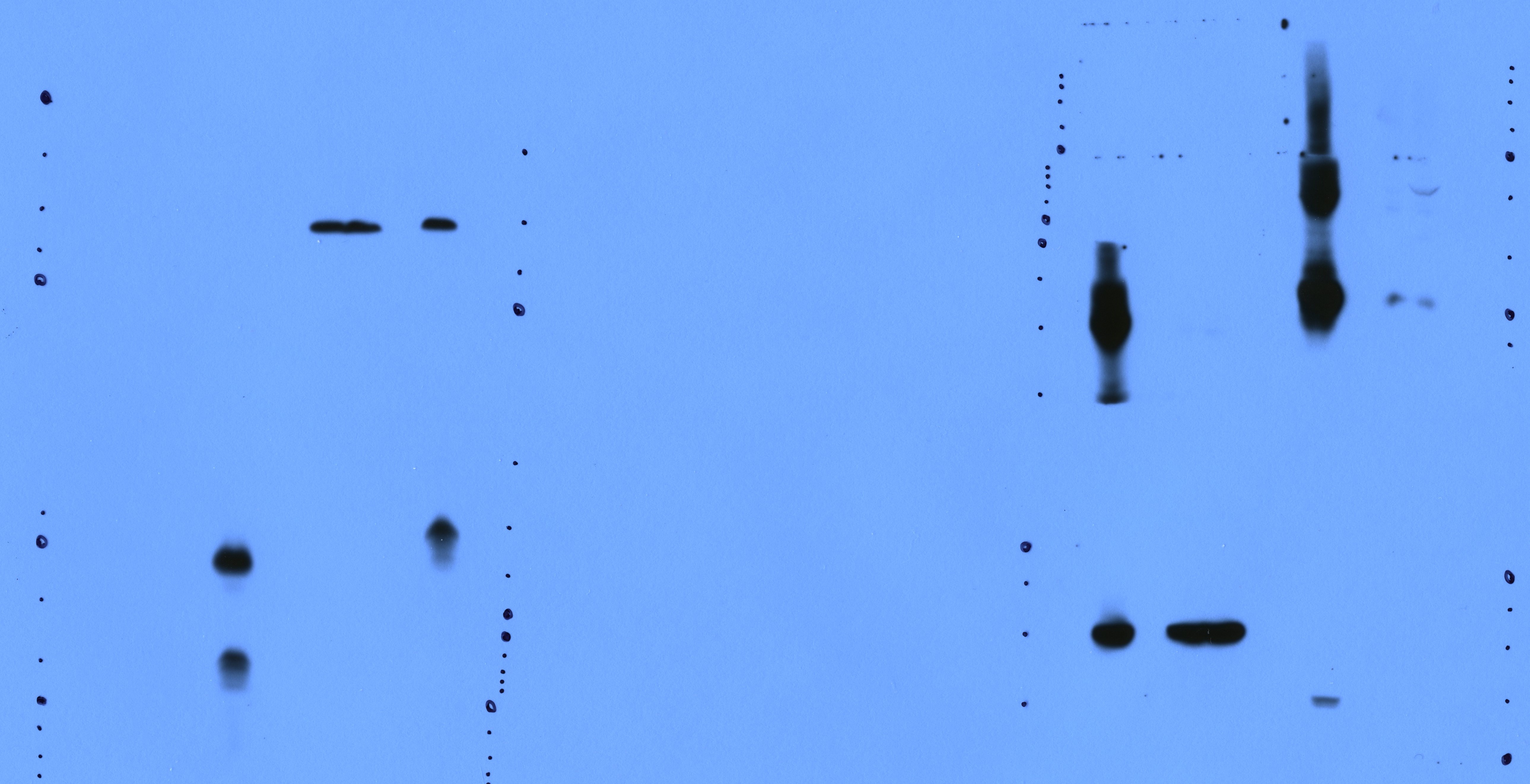

Supplement: Source data 3. [file elife-70079-supp3.zip › Source Data 3_Uncropped WB images for Main Figures/Figure 5I_actin-p21-p53.jpg]

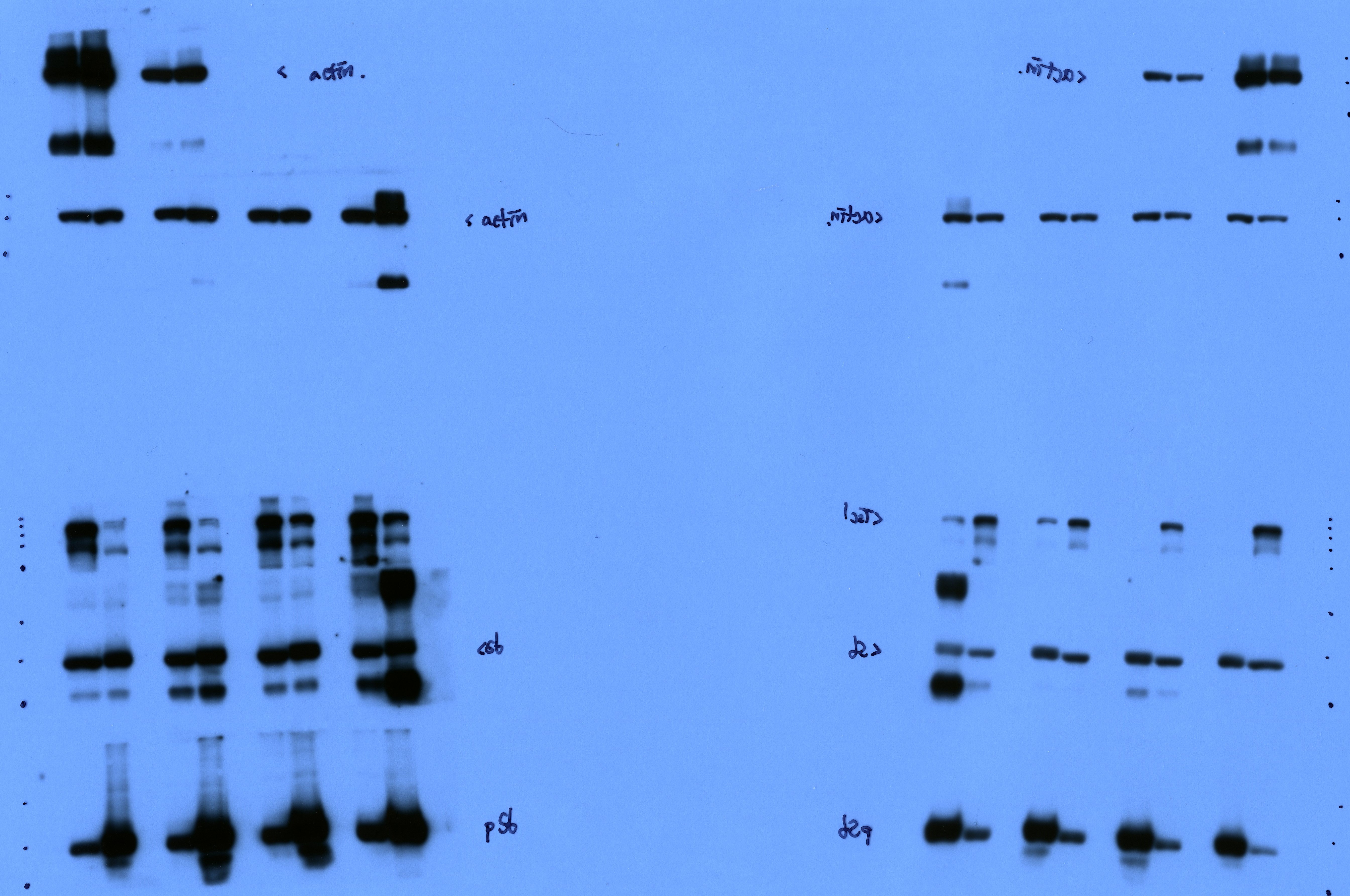

Supplement: Source data 3. [file elife-70079-supp3.zip › Source Data 3_Uncropped WB images for Main Figures/Figure 5C_actin-Tsc1-pS6-2.jpg]

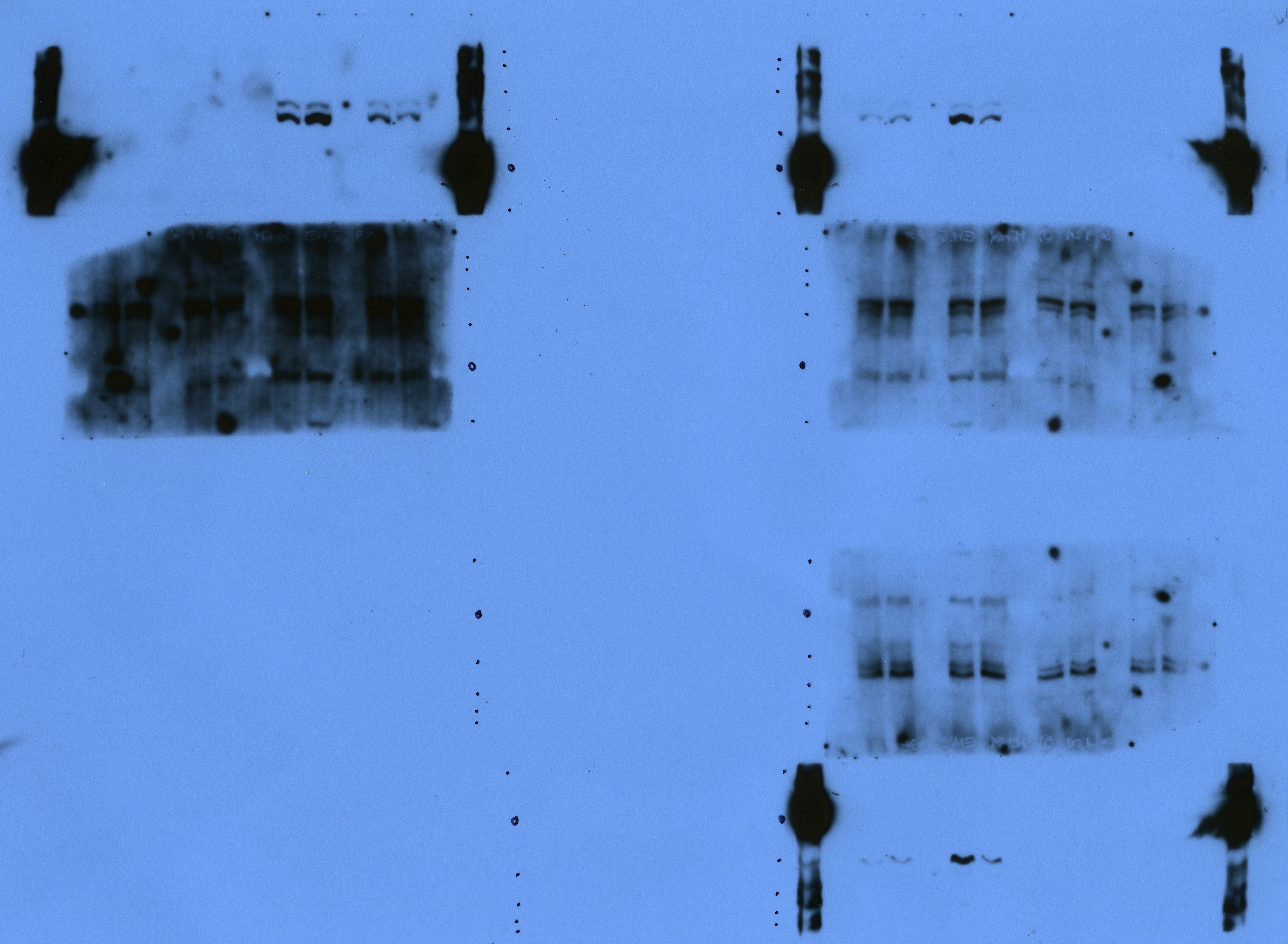

Supplement: Source data 3. [file elife-70079-supp3.zip › Source Data 3_Uncropped WB images for Main Figures/Figure 7E_HK2.jpg]

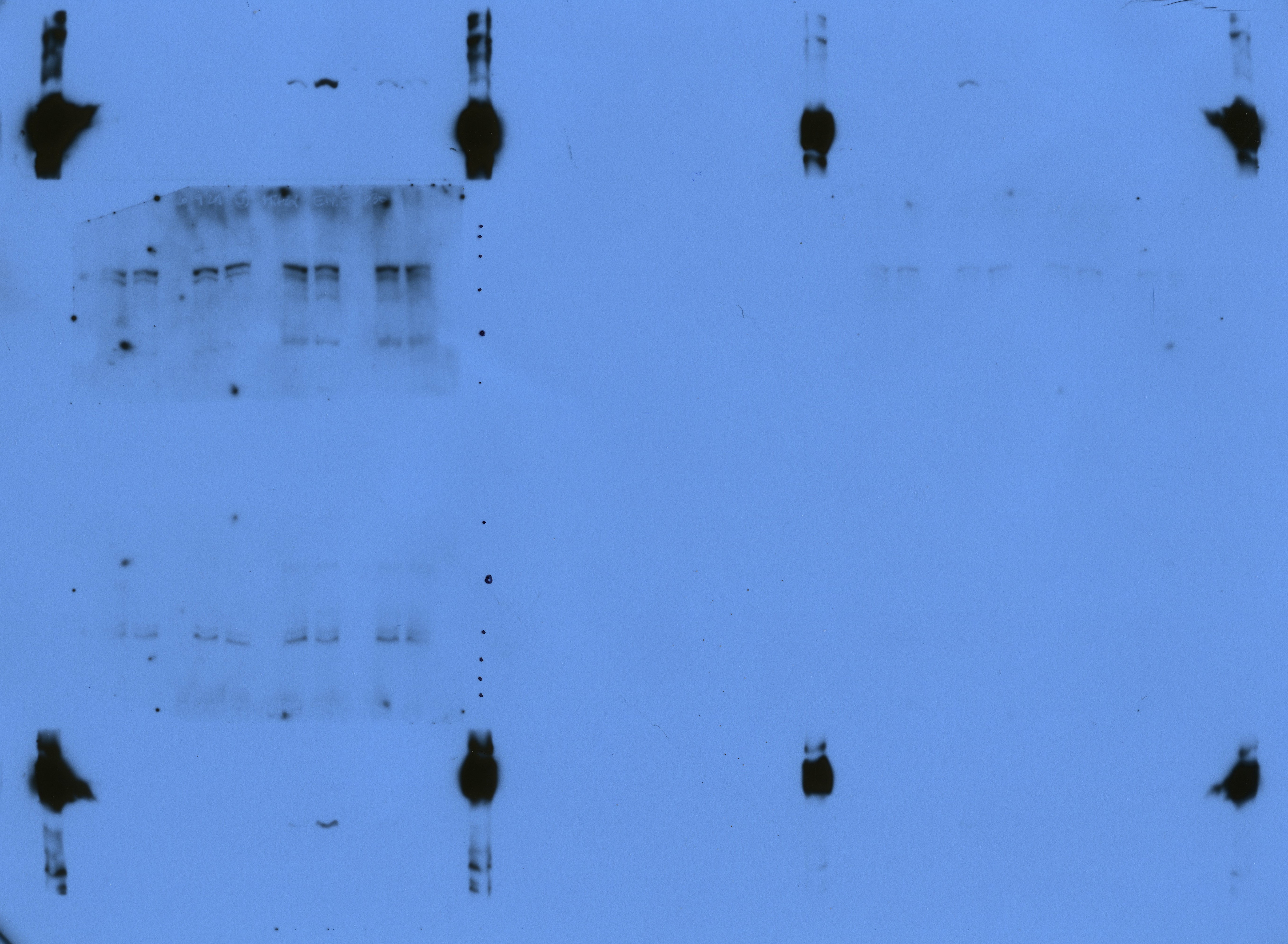

Supplement: Source data 3. [file elife-70079-supp3.zip › Source Data 3_Uncropped WB images for Main Figures/Figure 6A_Hif2a.jpg]

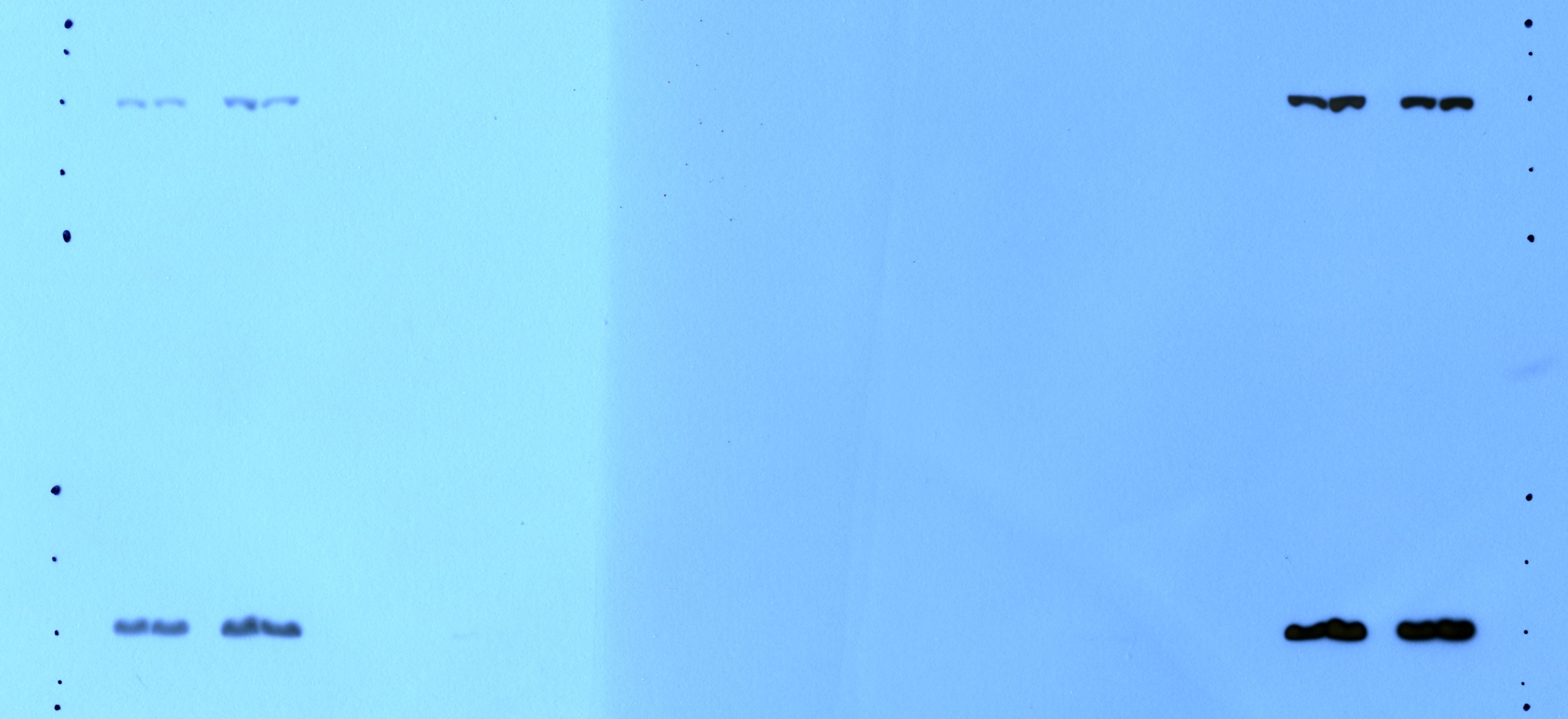

Supplement: Source data 3. [file elife-70079-supp3.zip › Source Data 3_Uncropped WB images for Main Figures/Figure 7E_actin.jpg]

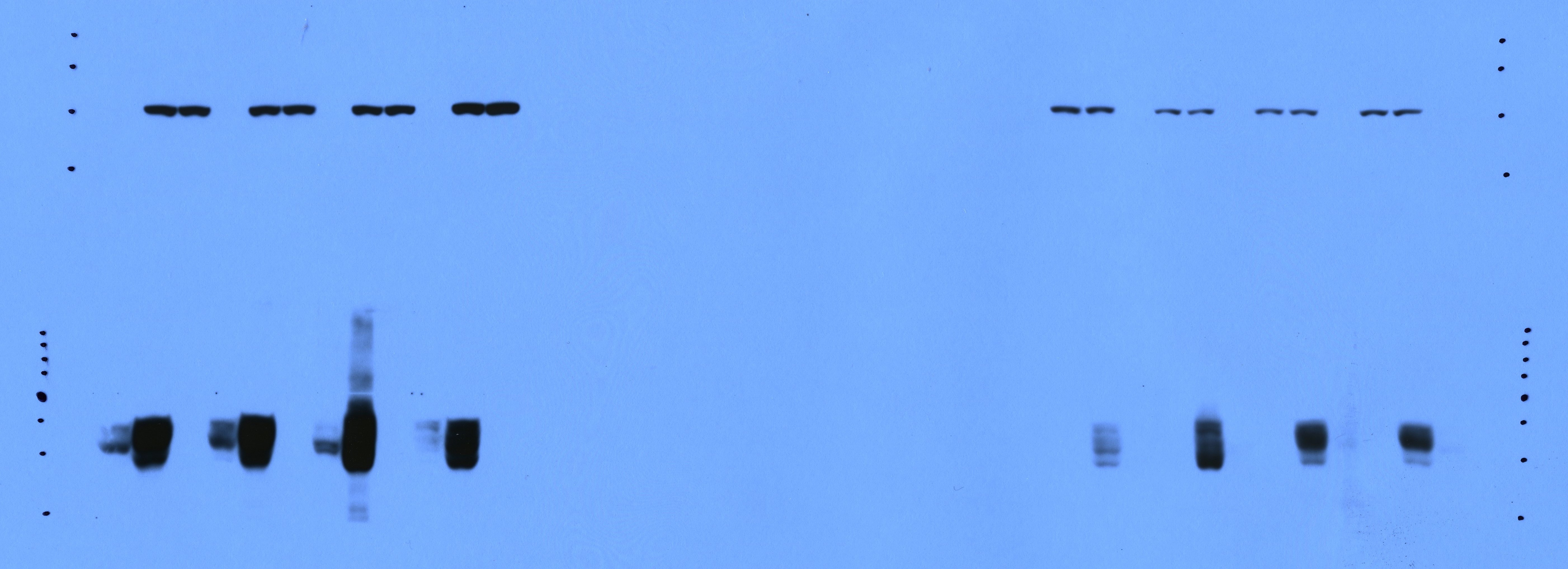

Supplement: Source data 3. [file elife-70079-supp3.zip › Source Data 3_Uncropped WB images for Main Figures/Figure 6A_actin.jpg]

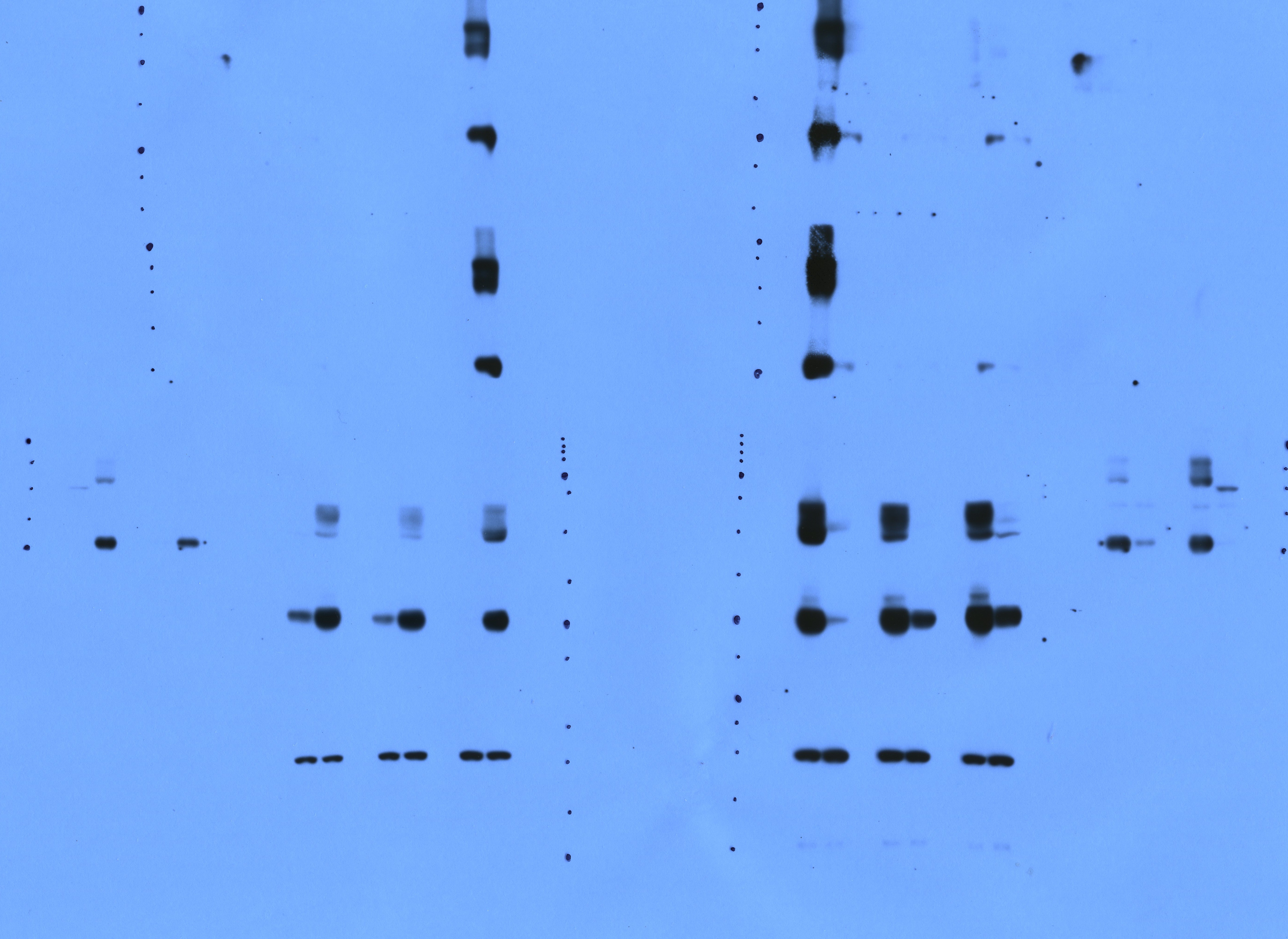

Supplement: Source data 3. [file elife-70079-supp3.zip › Source Data 3_Uncropped WB images for Main Figures/Figure 6A_cMyc.jpg]

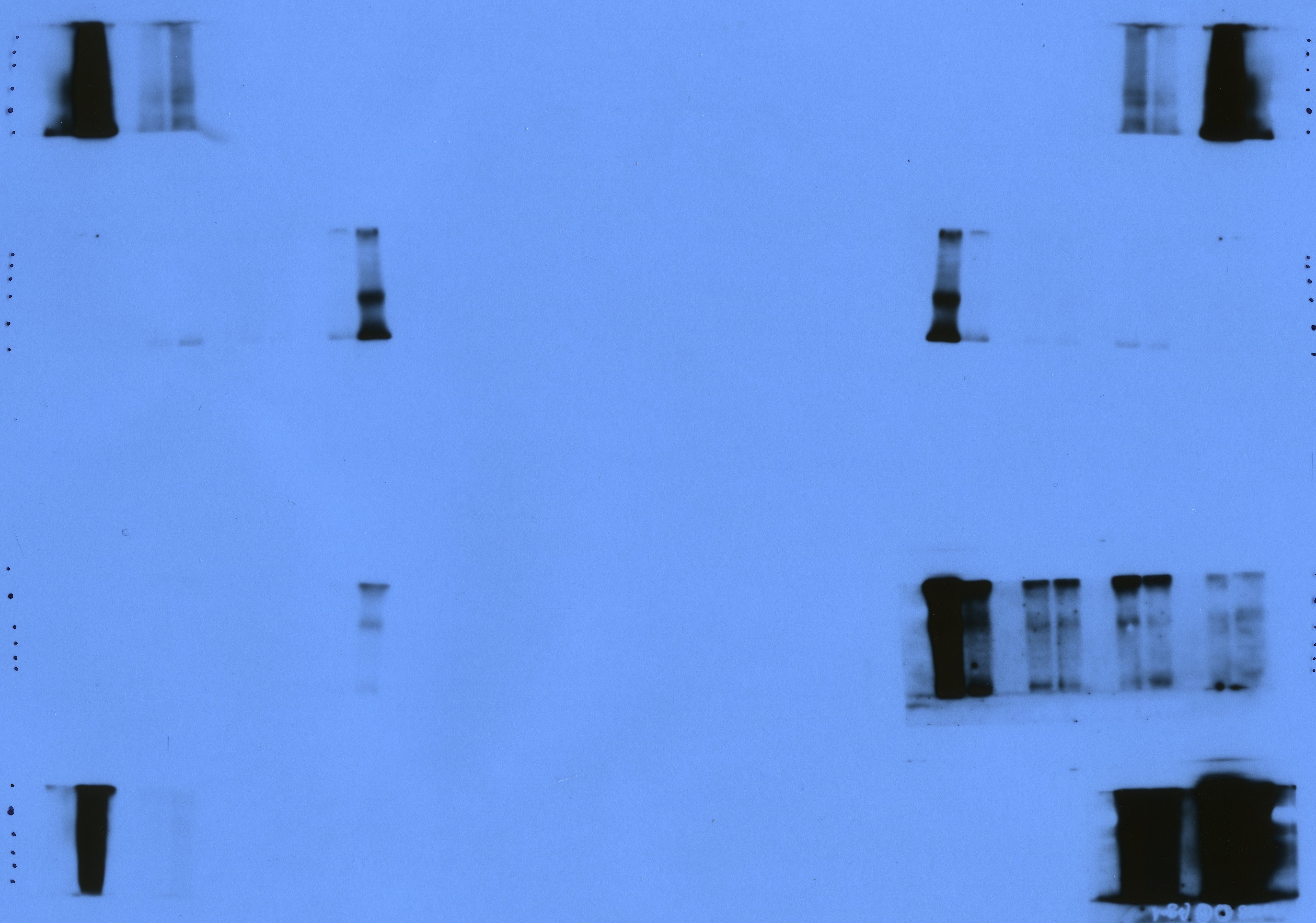

Supplement: Source data 3. [file elife-70079-supp3.zip › Source Data 3_Uncropped WB images for Main Figures/Figure 5C_betaGal.jpg]

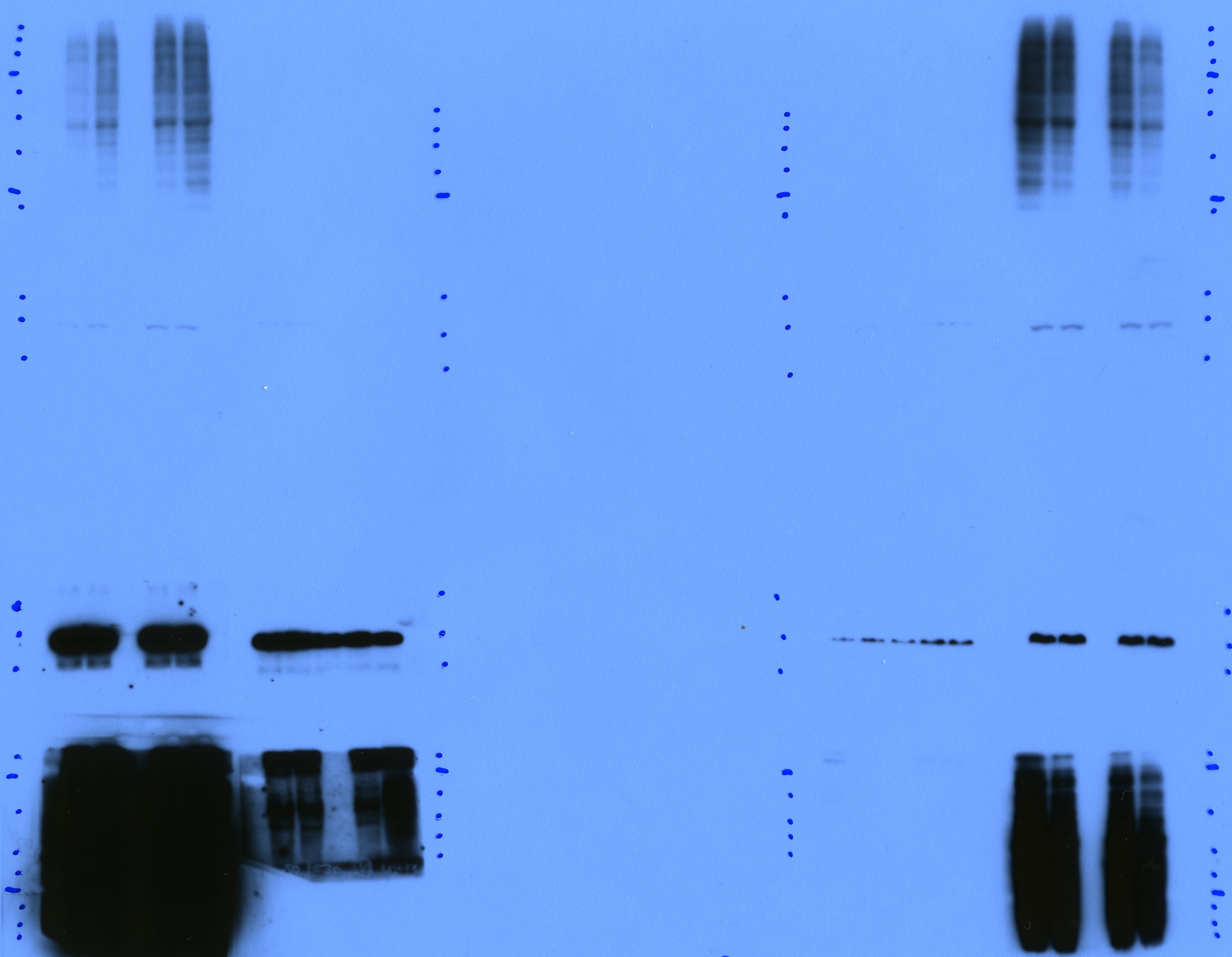

Supplement: Source data 3. [file elife-70079-supp3.zip › Source Data 3_Uncropped WB images for Main Figures/Figure 7E_Hypoxy.jpg]

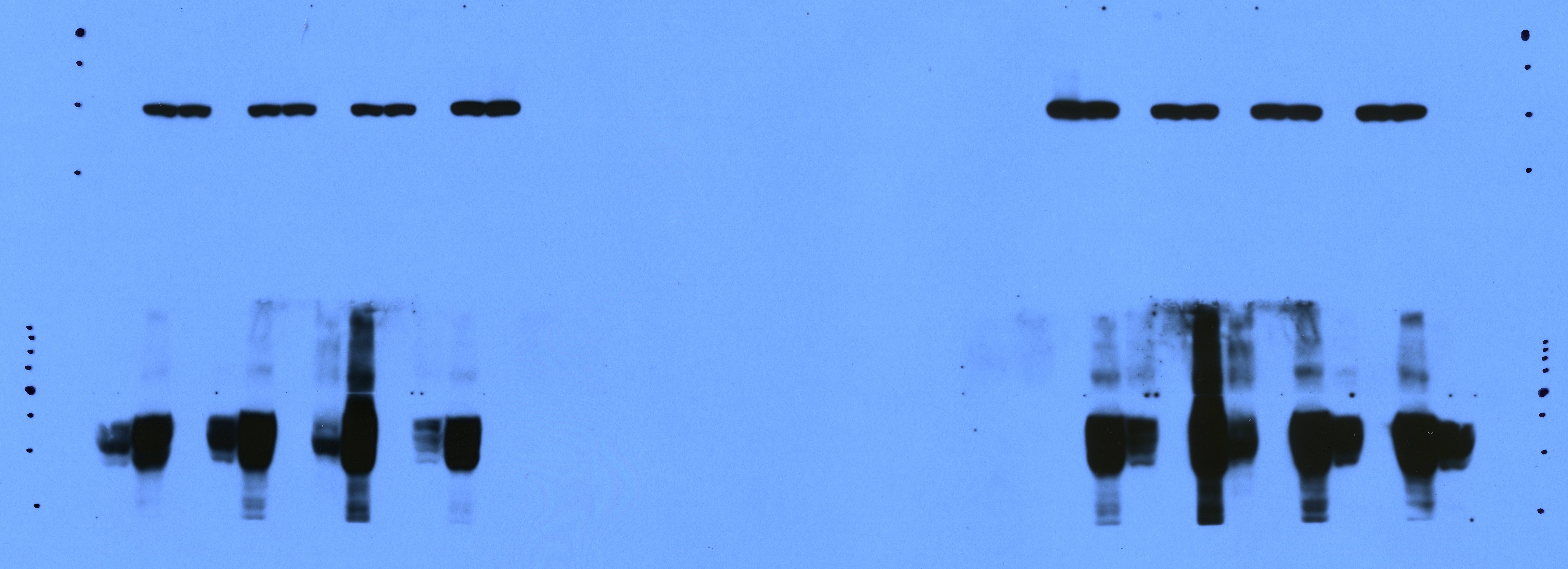

Supplement: Source data 3. [file elife-70079-supp3.zip › Source Data 3_Uncropped WB images for Main Figures/Figure 6A_betaGal.jpg]

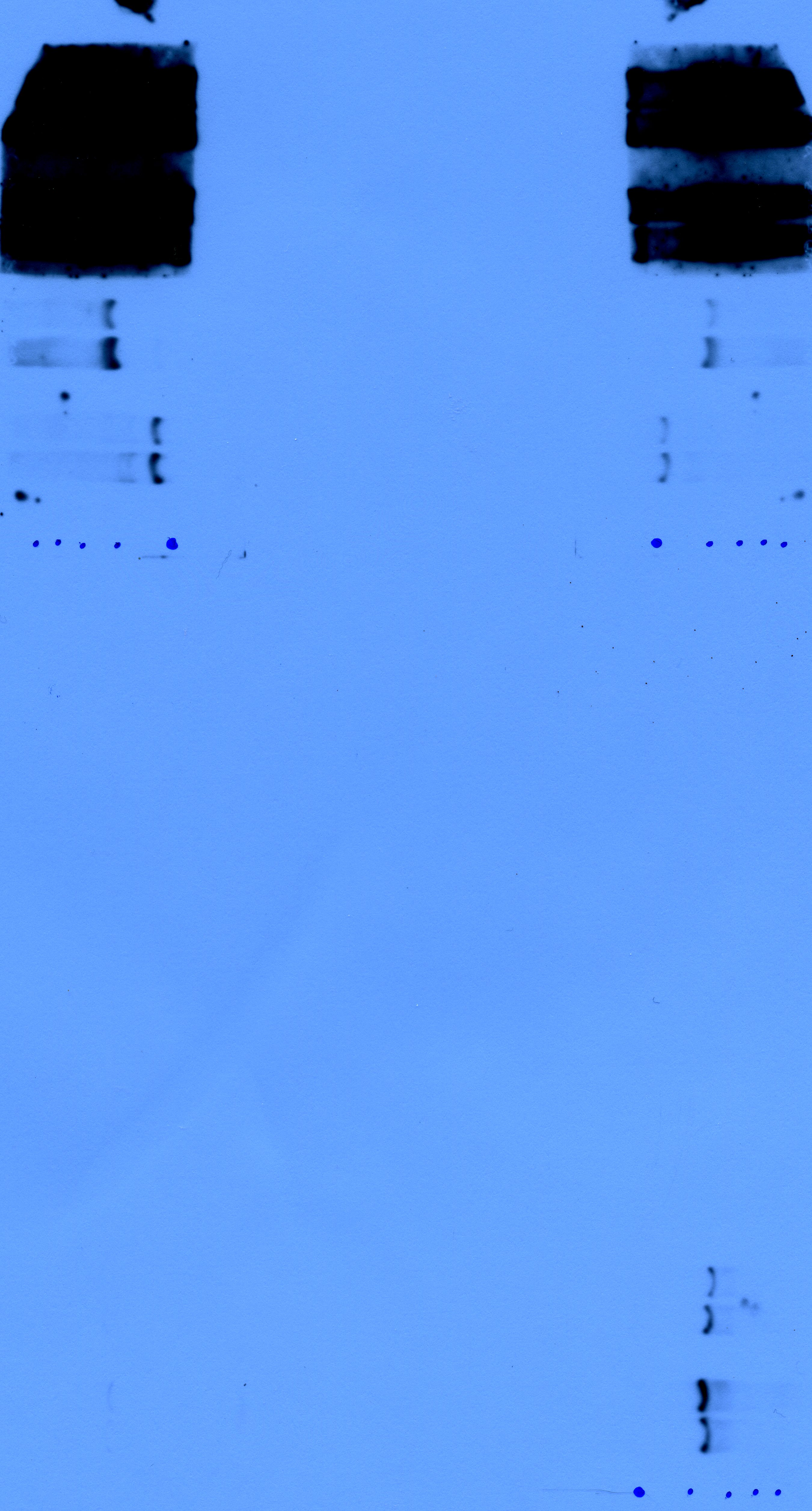

Supplement: Source data 3. [file elife-70079-supp3.zip › Source Data 3_Uncropped WB images for Main Figures/Figure 7E_Hif1a.jpg]

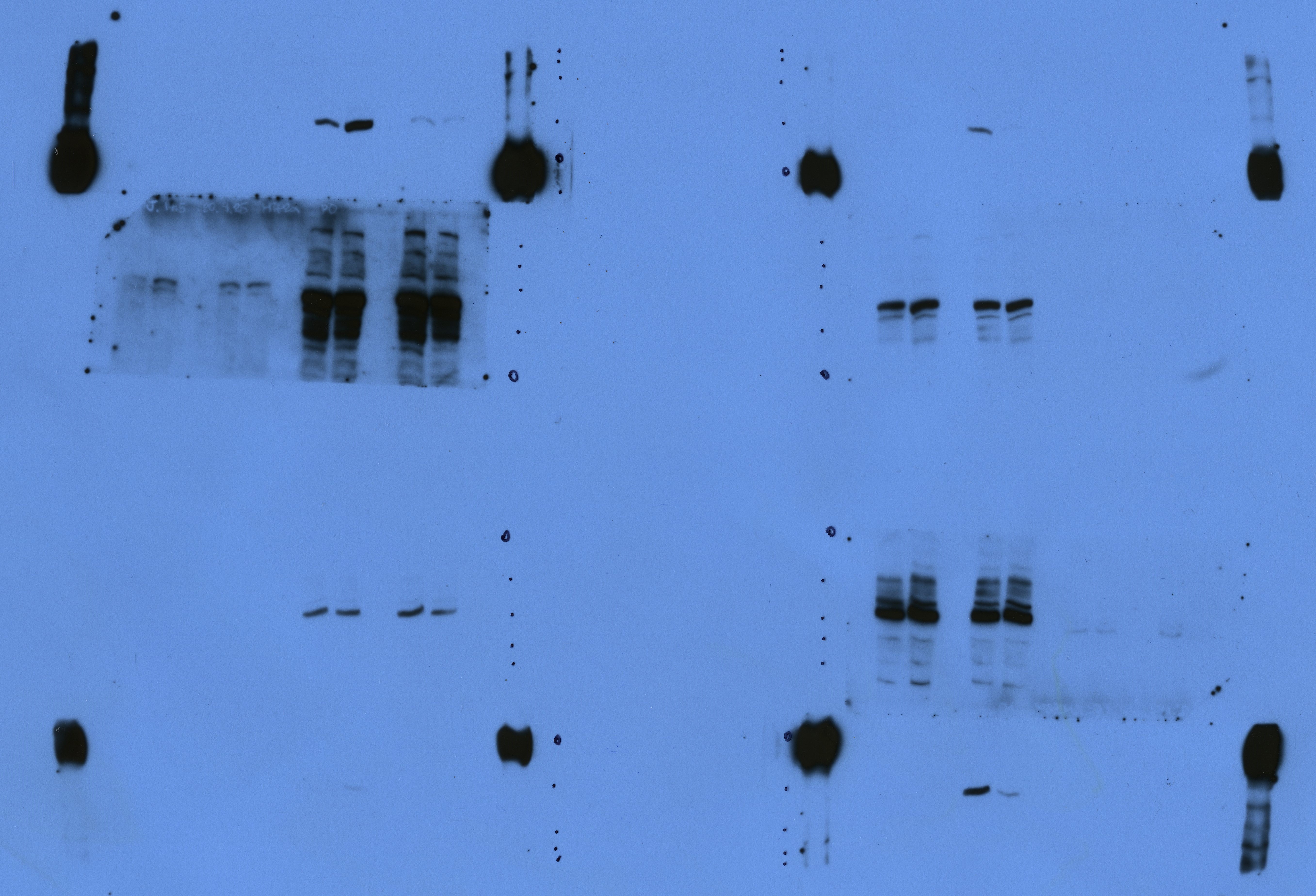

Supplement: Source data 3. [file elife-70079-supp3.zip › Source Data 3_Uncropped WB images for Main Figures/Figure 7E_Hif2a .jpg]

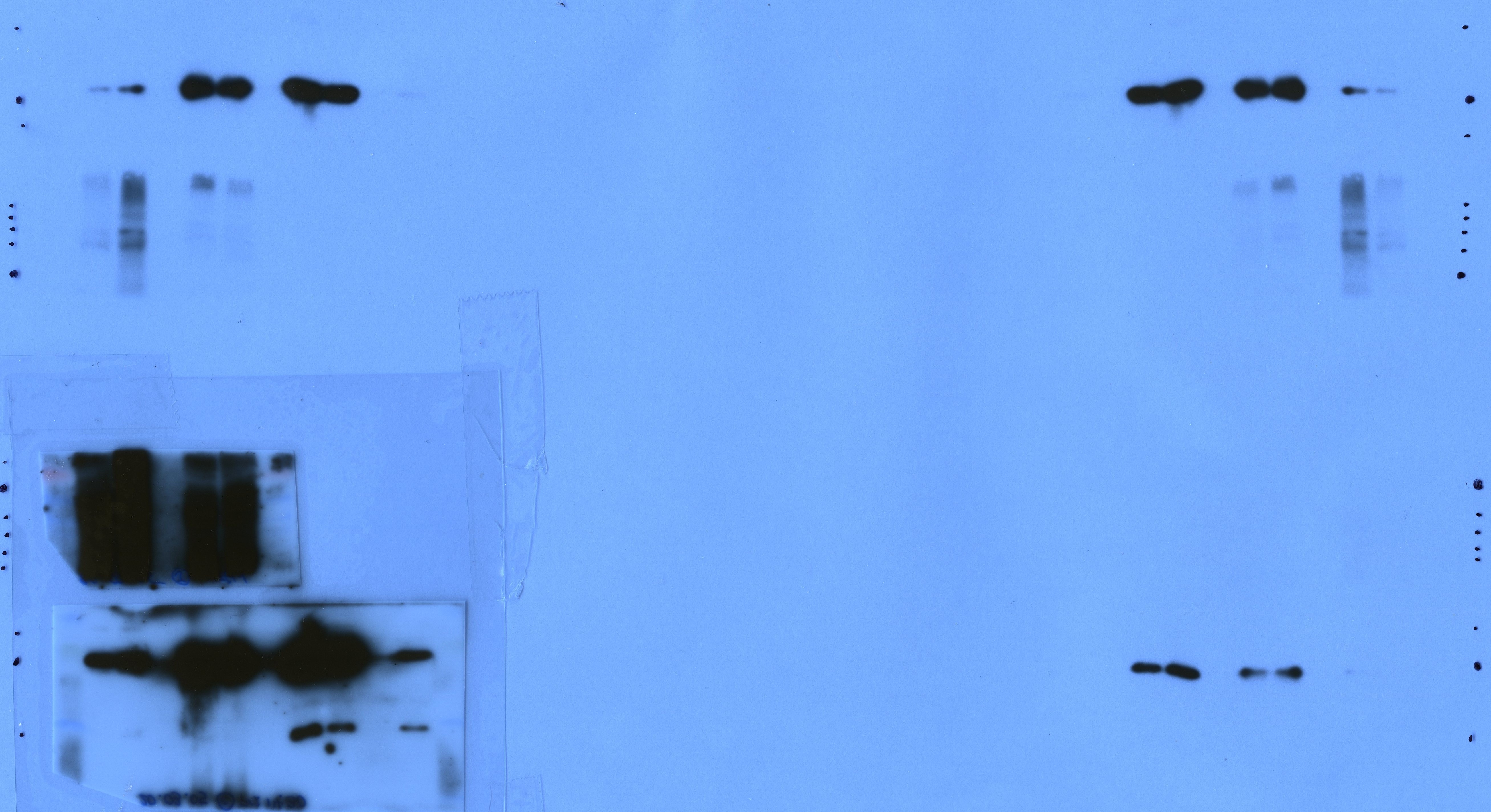

Supplement: Source data 3. [file elife-70079-supp3.zip › Source Data 3_Uncropped WB images for Main Figures/Figure 6A_Hif1a.jpg]

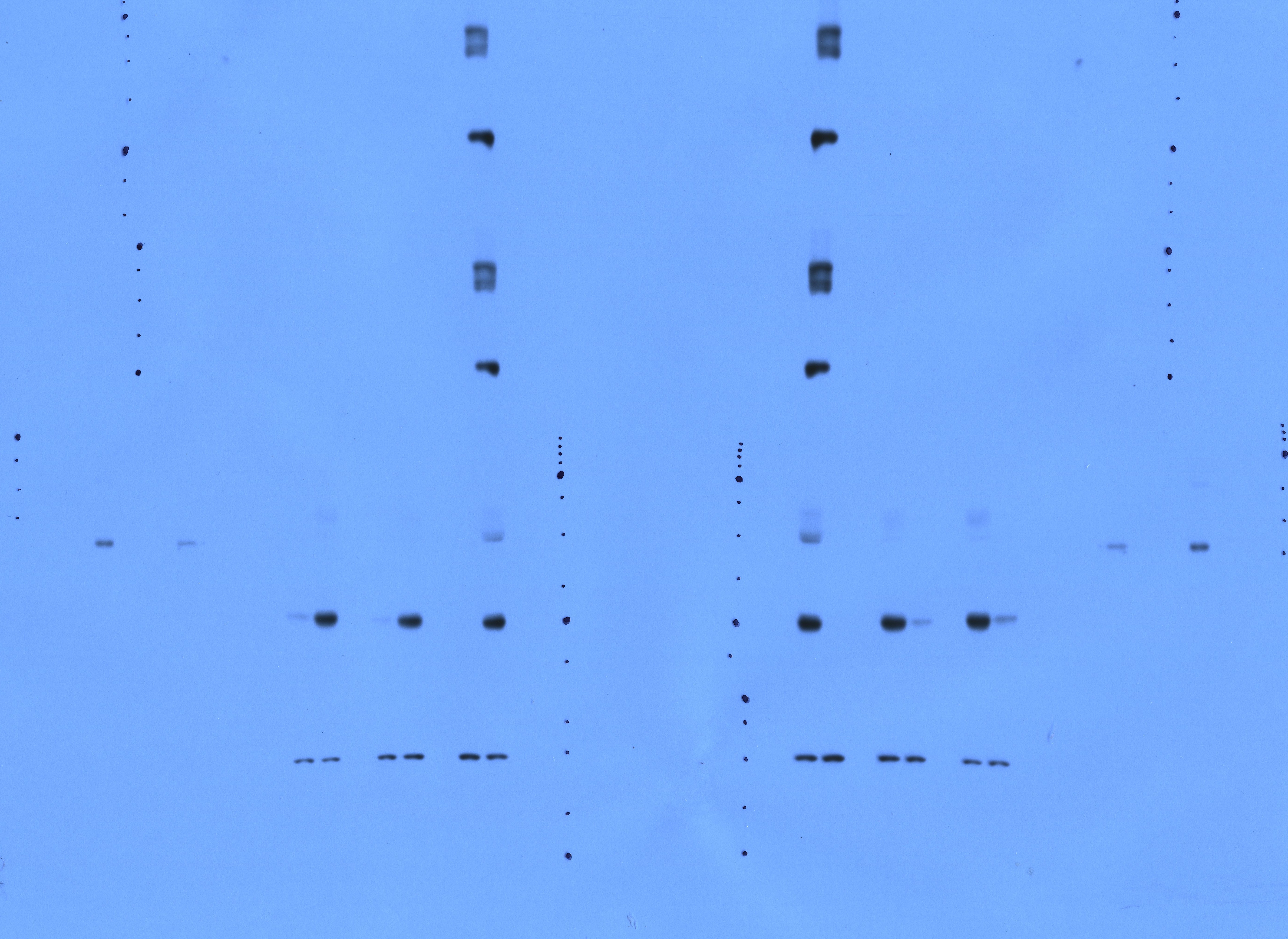

Supplement: Source data 3. [file elife-70079-supp3.zip › Source Data 3_Uncropped WB images for Main Figures/Figure 5C_p53-p21.jpg]

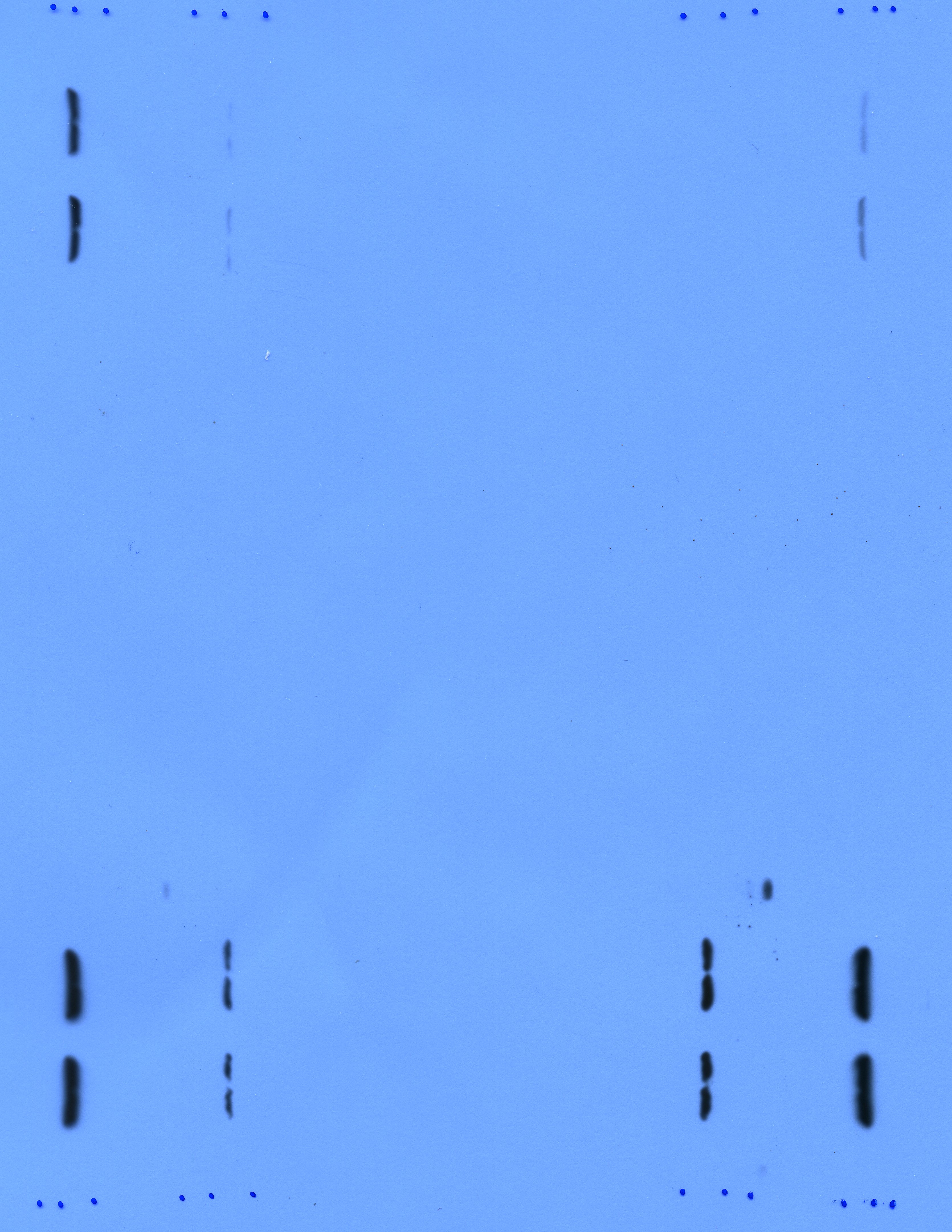

Supplement: Source data 3. [file elife-70079-supp3.zip › Source Data 3_Uncropped WB images for Main Figures/Figure 7E_Tom20.jpg]

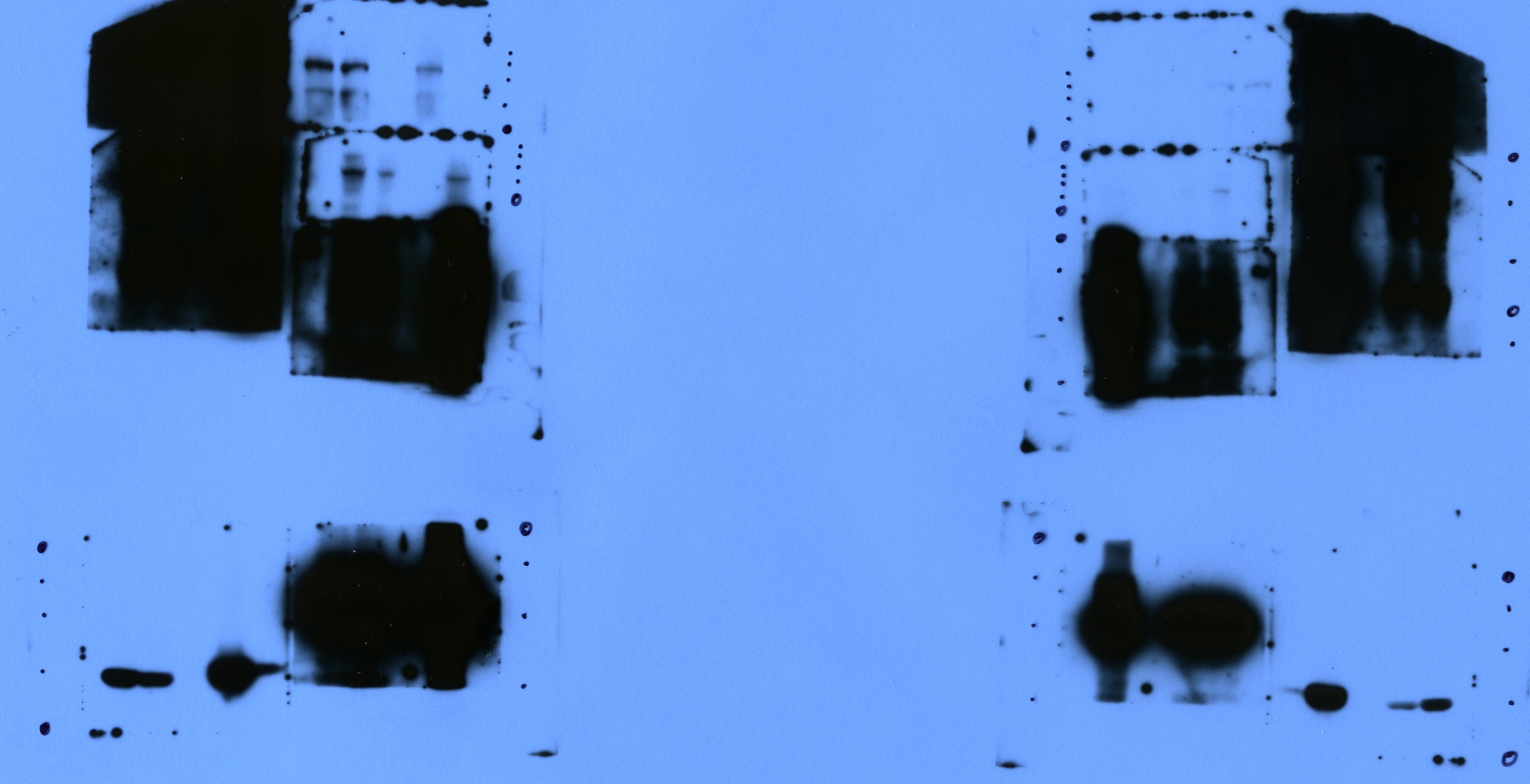

Supplement: Source data 3. [file elife-70079-supp3.zip › Source Data 3_Uncropped WB images for Main Figures/Figure 5I_Raptor.jpg]

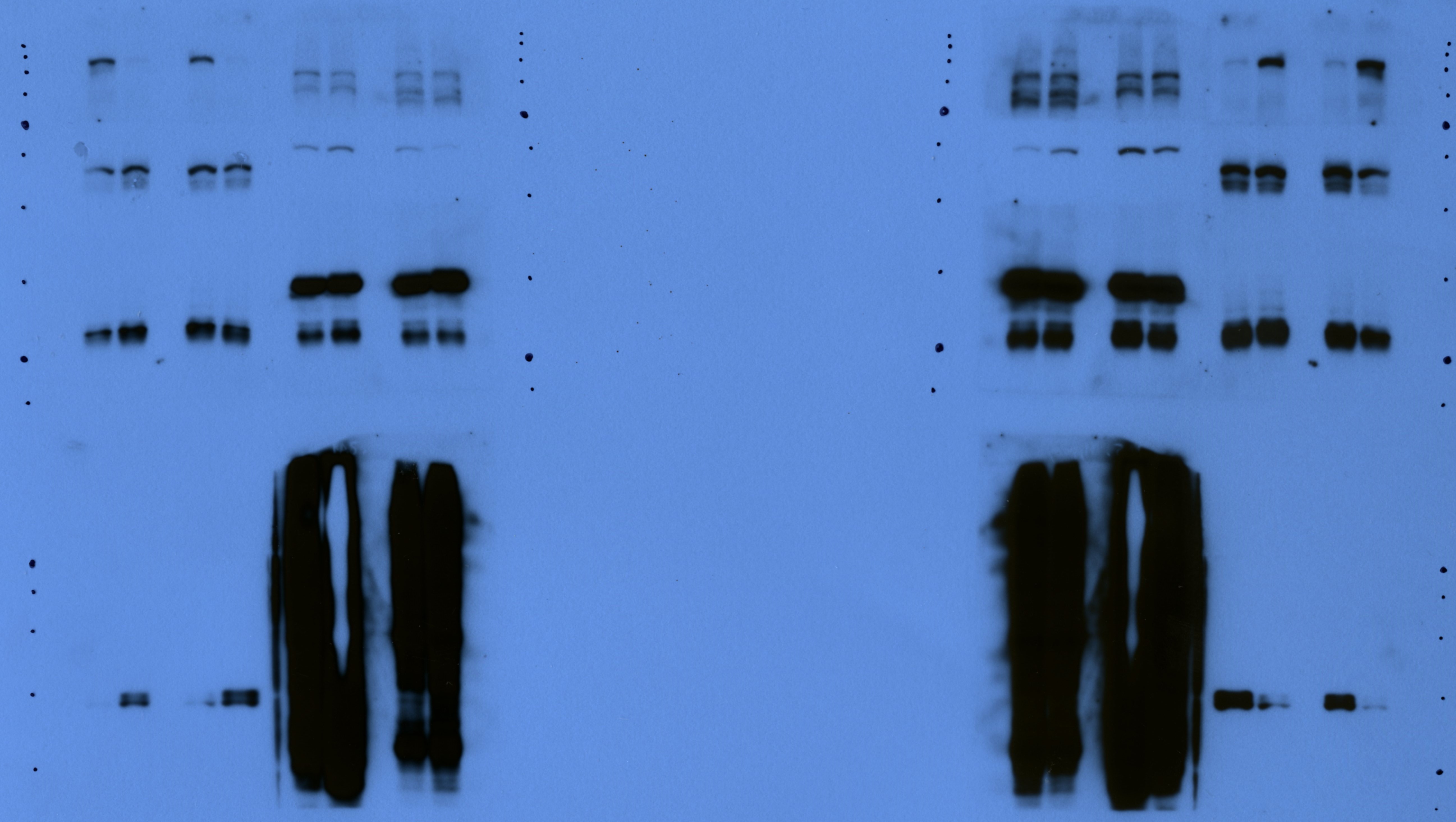

Supplement: Source data 3. [file elife-70079-supp3.zip › Source Data 3_Uncropped WB images for Main Figures/Figure 7E_PKM2-pS6.jpg]

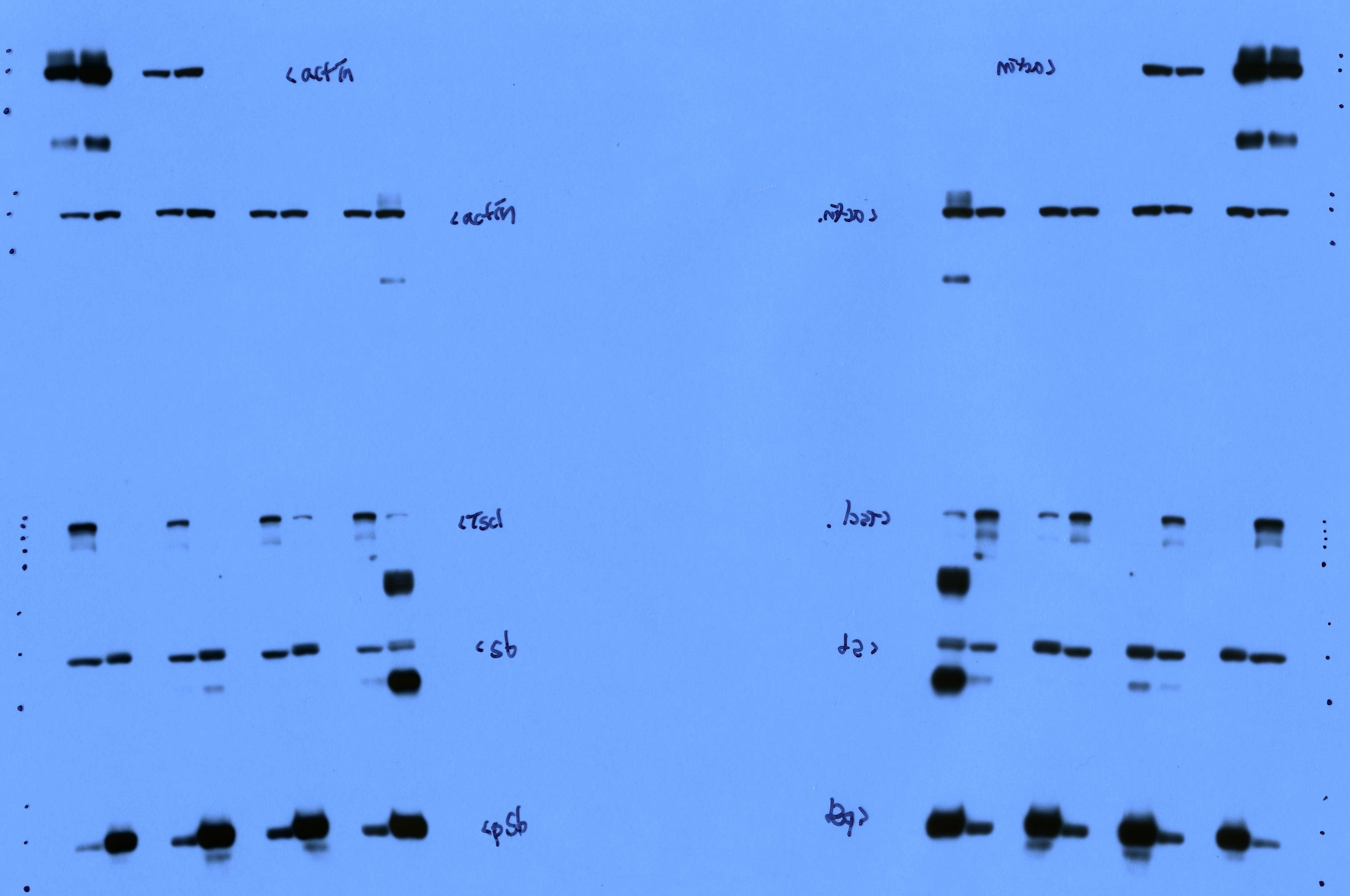

Supplement: Source data 3. [file elife-70079-supp3.zip › Source Data 3_Uncropped WB images for Main Figures/Figure 5C_actin-Tsc1-pS6.jpg]

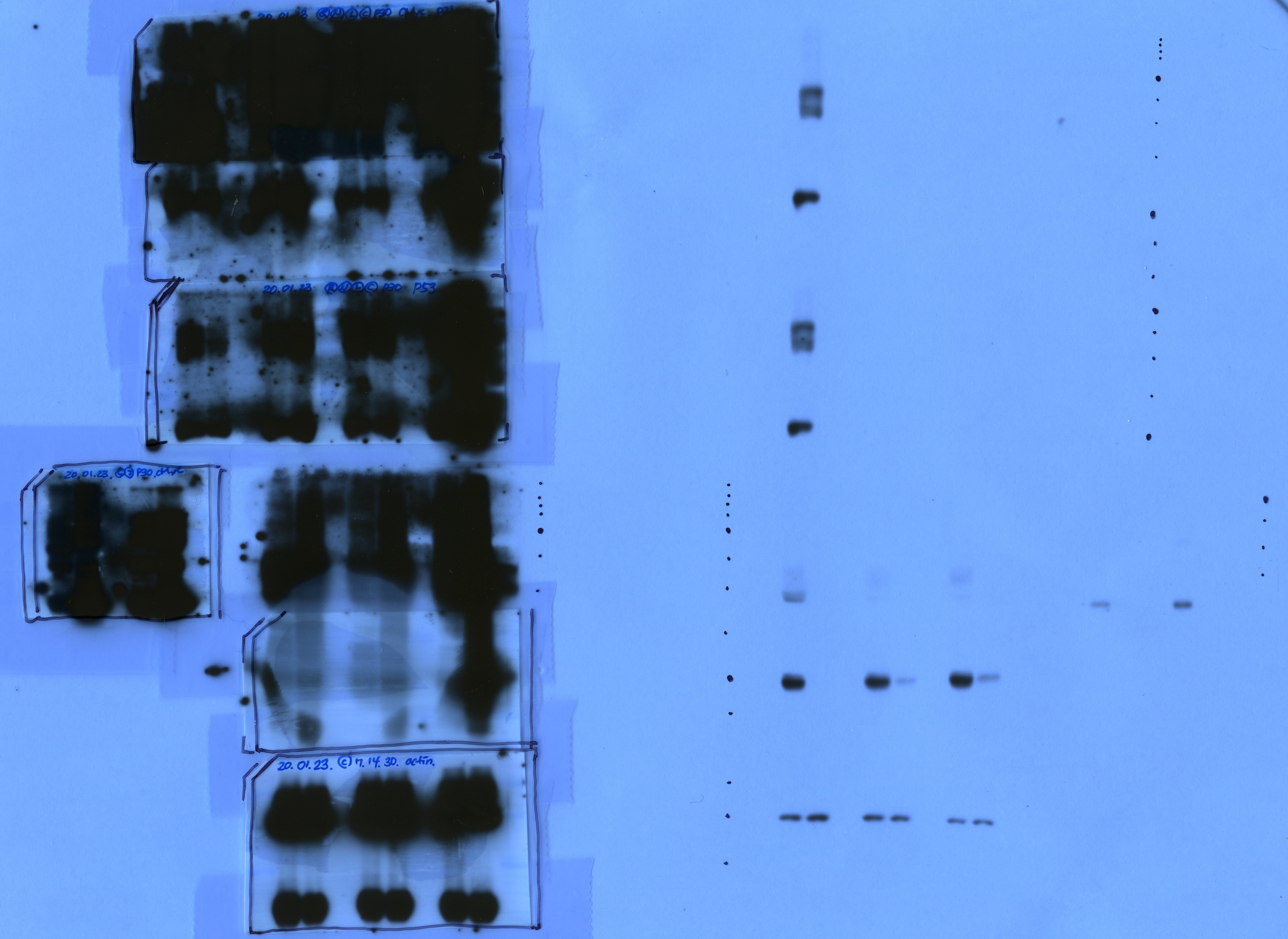

Supplement: Source data 3. [file elife-70079-supp3.zip › Source Data 3_Uncropped WB images for Main Figures/Figure 5C_cMyc.jpg]

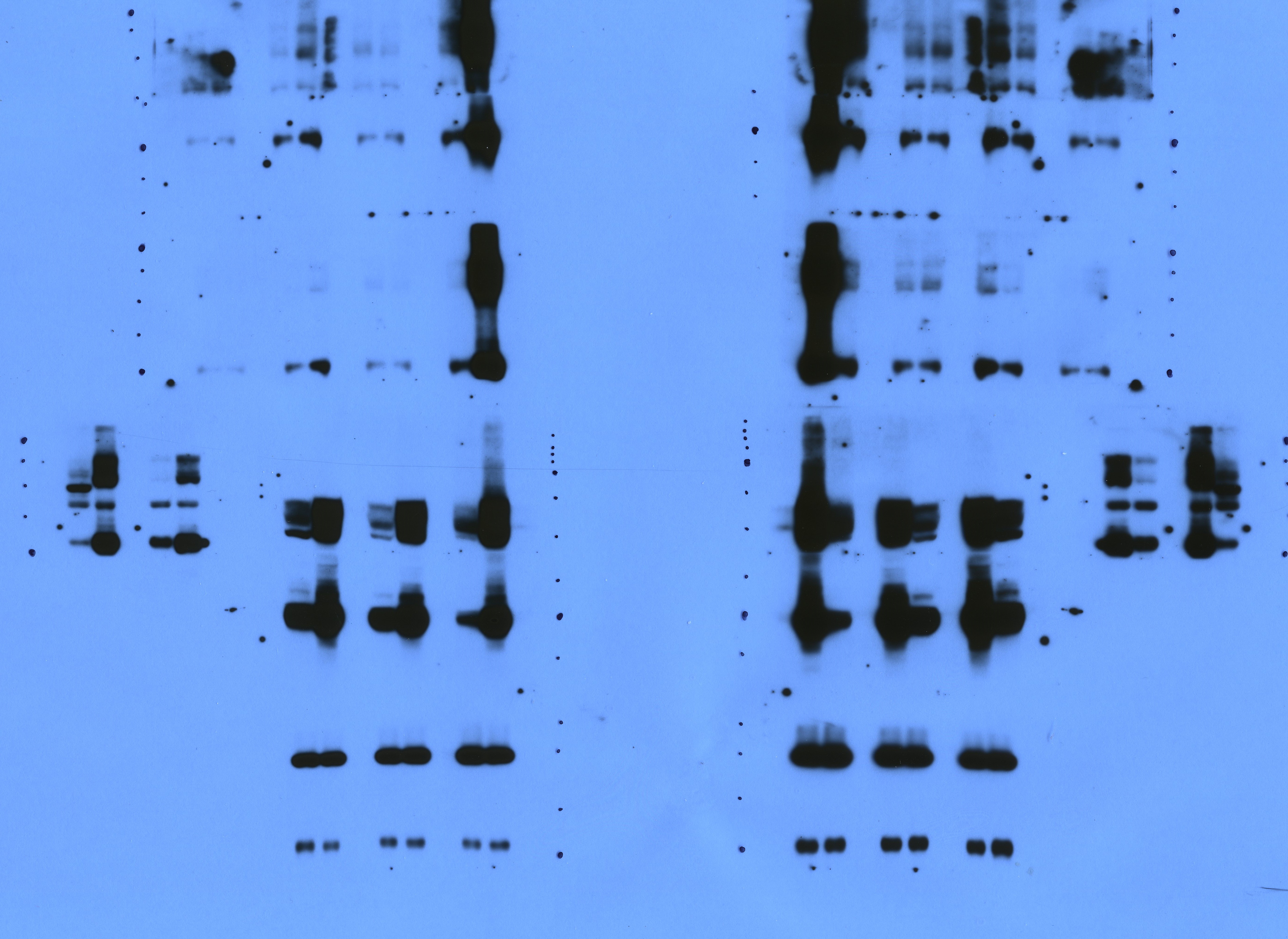

Supplement: Source data 4. [file elife-70079-supp4.zip › Source Data 4_Uncropped images for figure supplements/Figure S8_p7-14-30-betaGal.jpg]

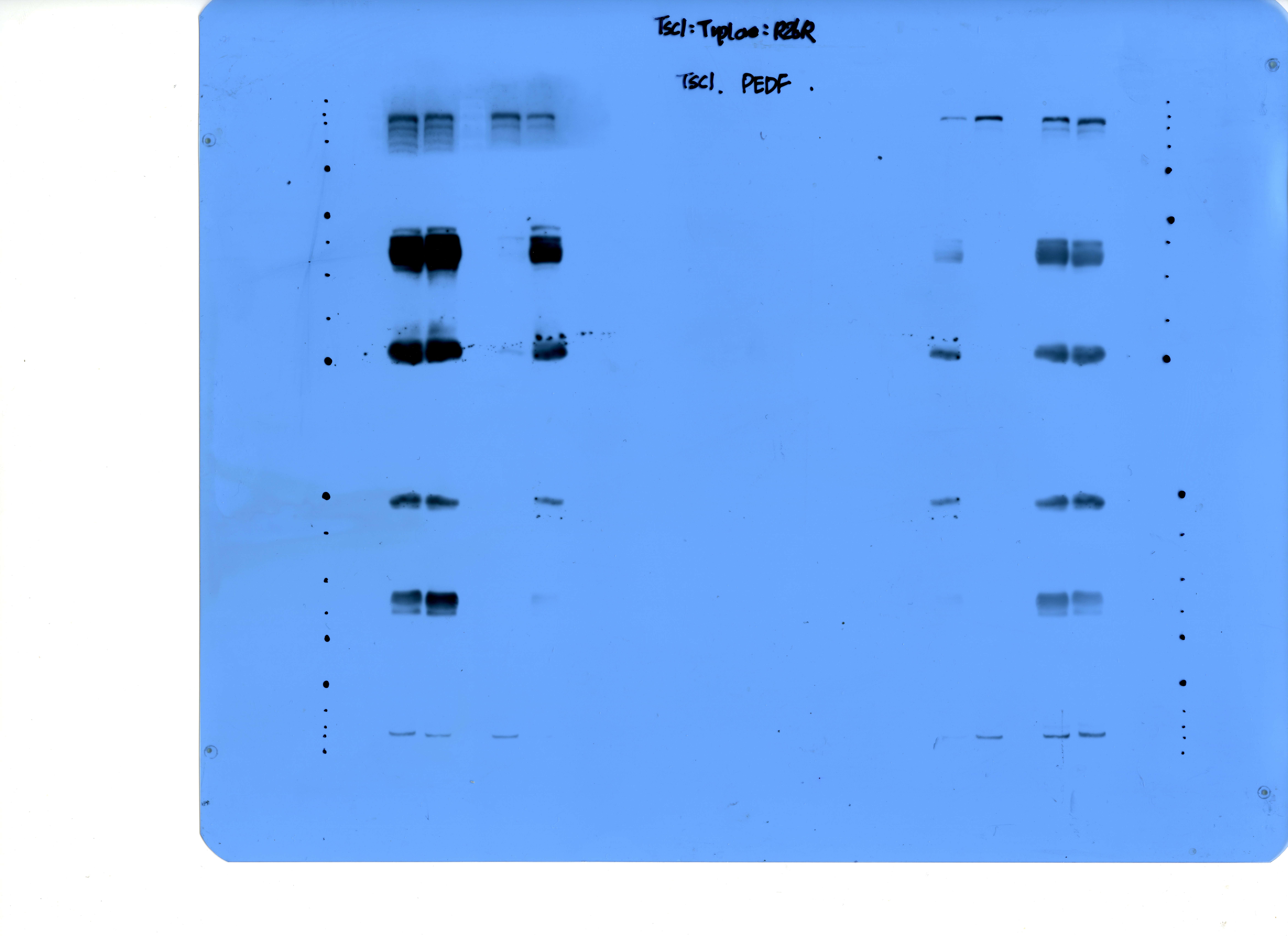

Supplement: Source data 4. [file elife-70079-supp4.zip › Source Data 4_Uncropped images for figure supplements/Figure S2B_TSC1-p30(RPE).jpg]

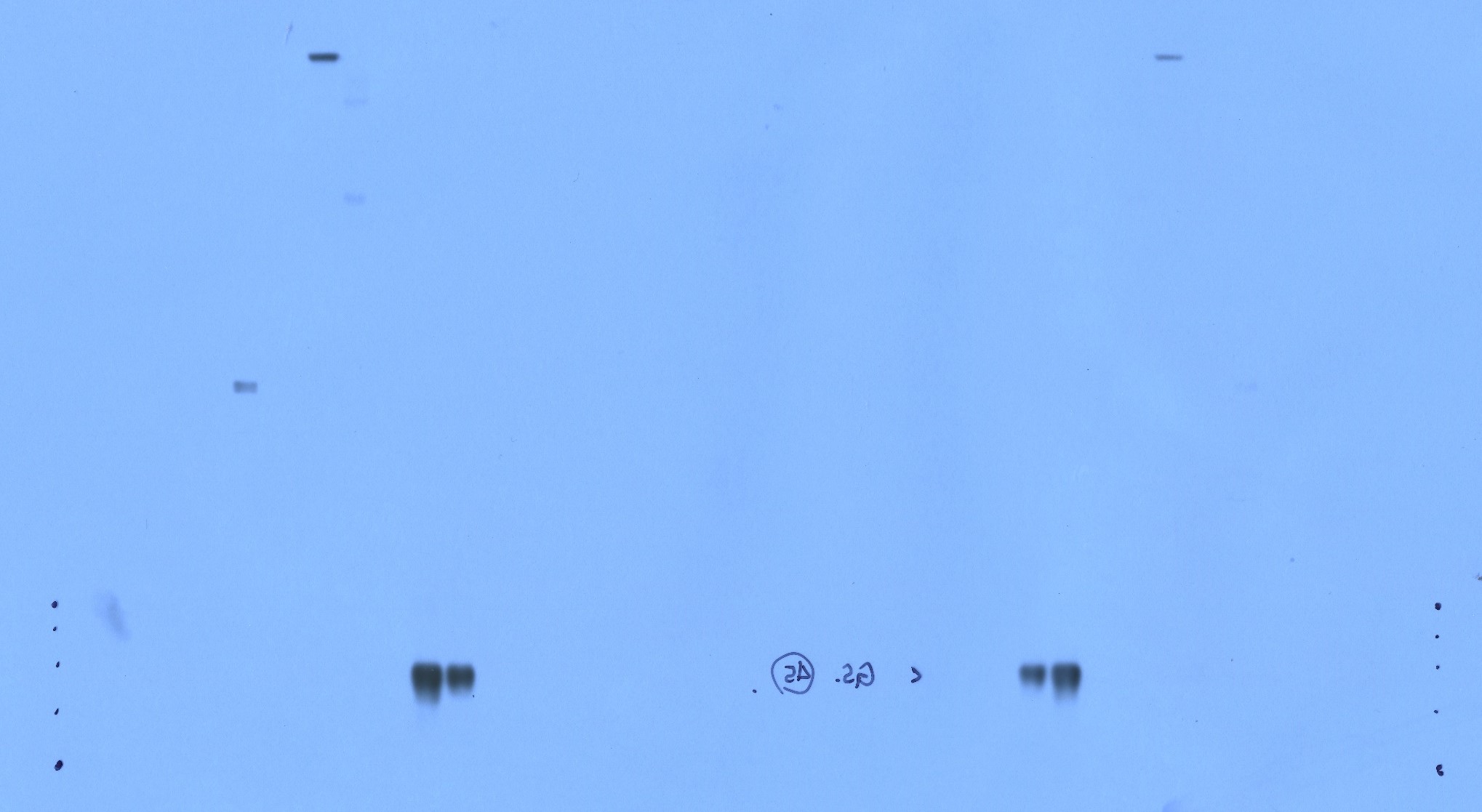

Supplement: Source data 4. [file elife-70079-supp4.zip › Source Data 4_Uncropped images for figure supplements/Figure S2B_GS-p30(RPE, Retina).jpg]

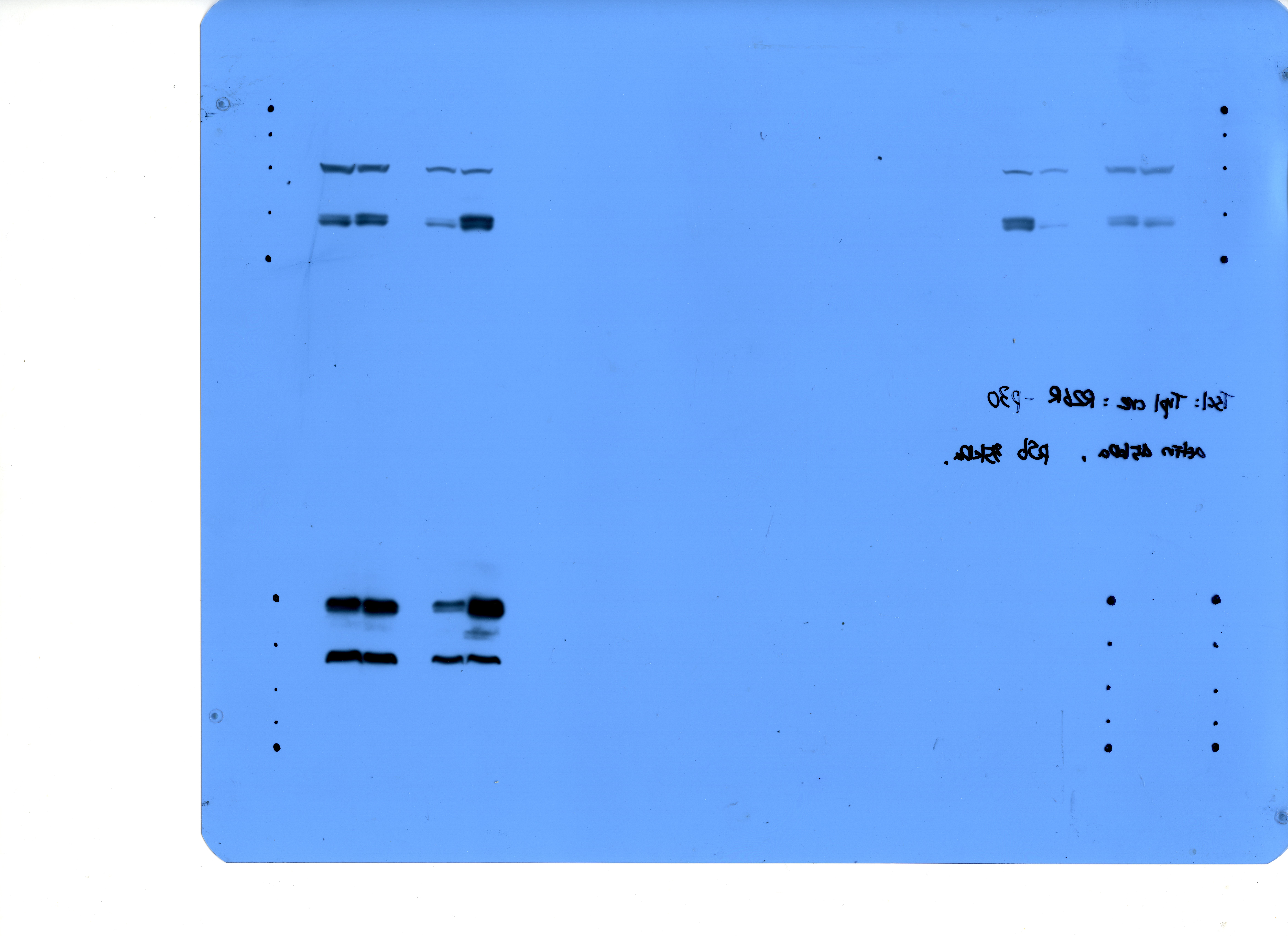

Supplement: Source data 4. [file elife-70079-supp4.zip › Source Data 4_Uncropped images for figure supplements/Figure S2B_pS6-p30(Retina).jpg]

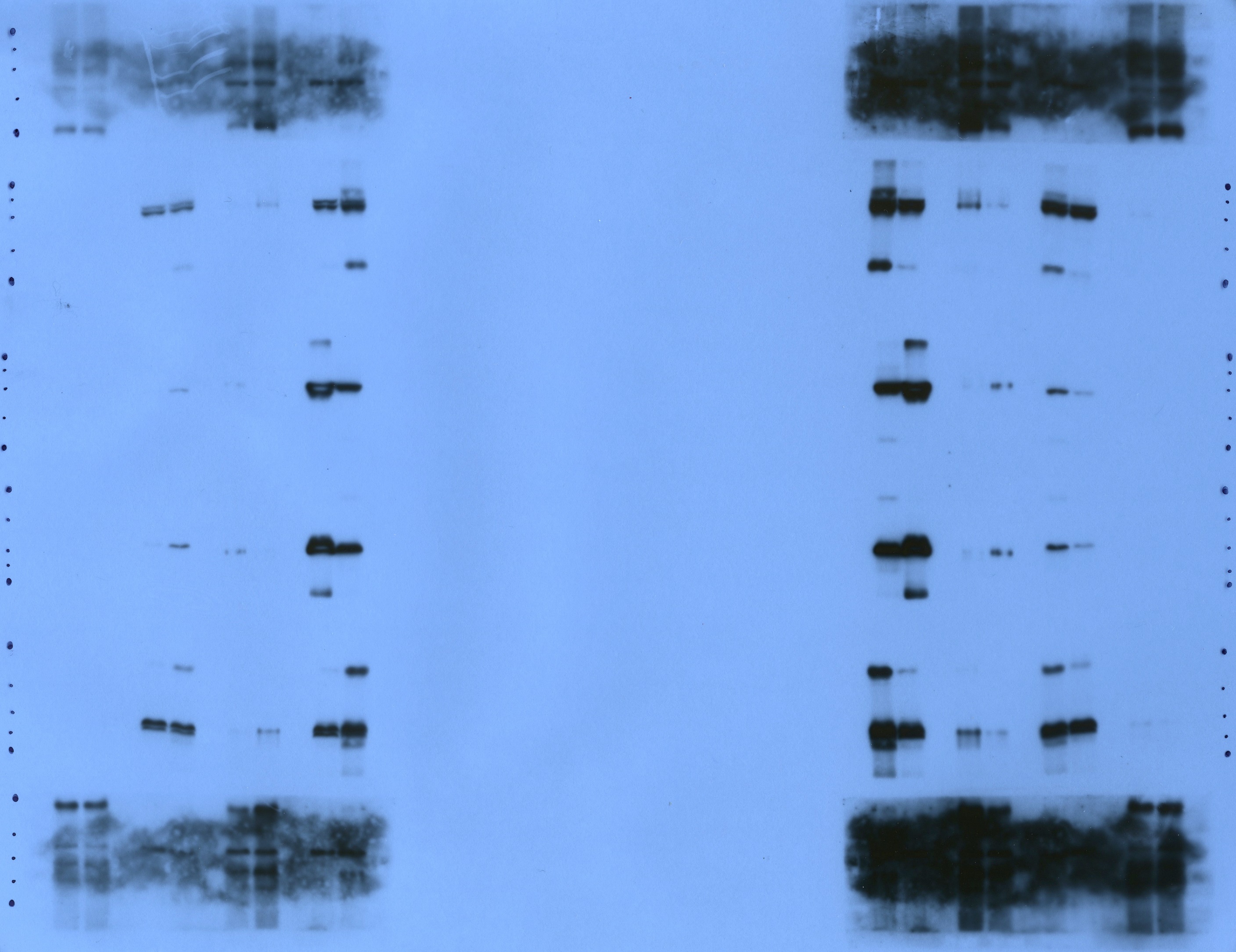

Supplement: Source data 4. [file elife-70079-supp4.zip › Source Data 4_Uncropped images for figure supplements/Figure S2B_GS-p7(RPE, Retina).jpg]

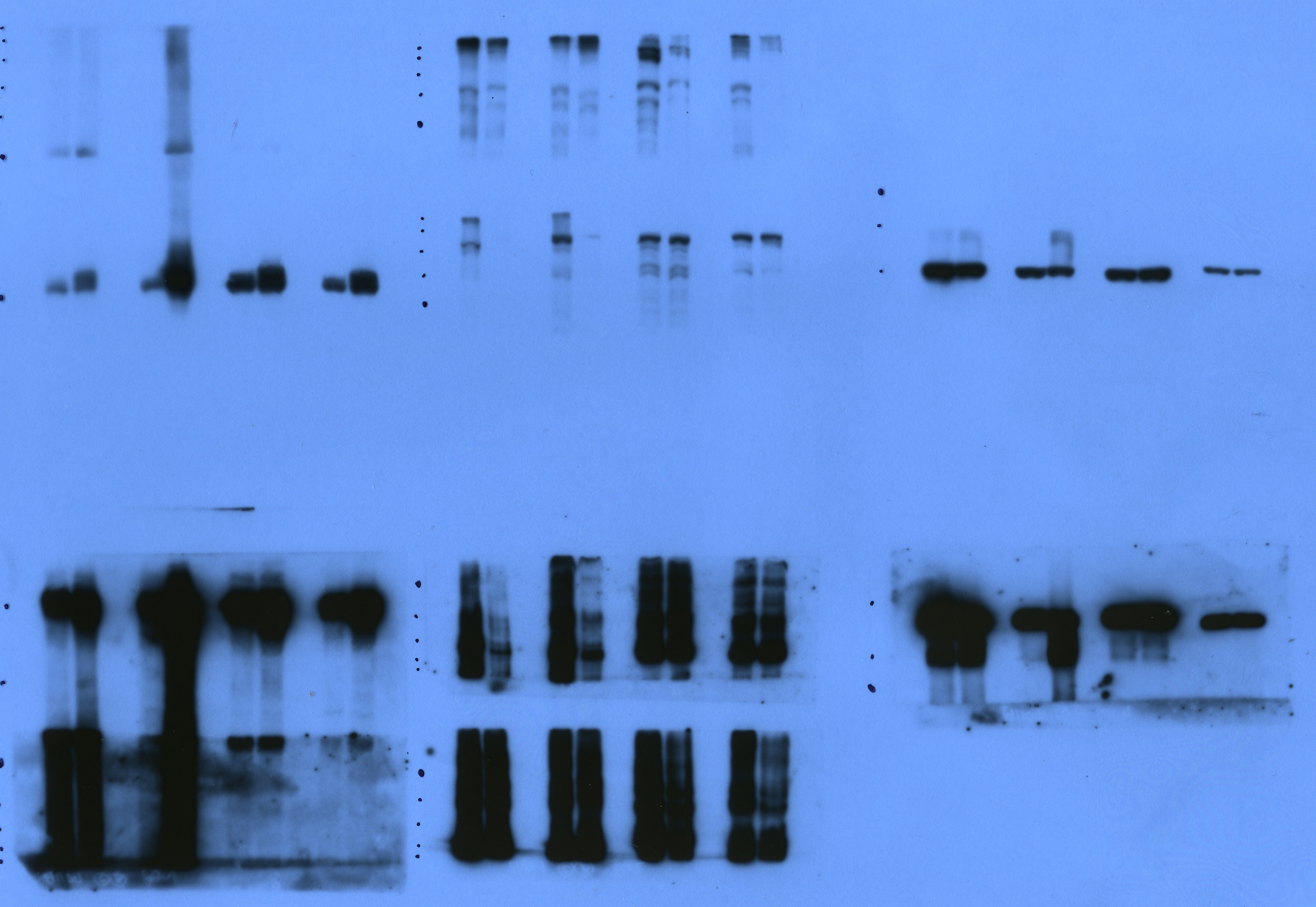

Supplement: Source data 4. [file elife-70079-supp4.zip › Source Data 4_Uncropped images for figure supplements/Figure S8_Tsc2ko-P30-Tsc2.jpg]

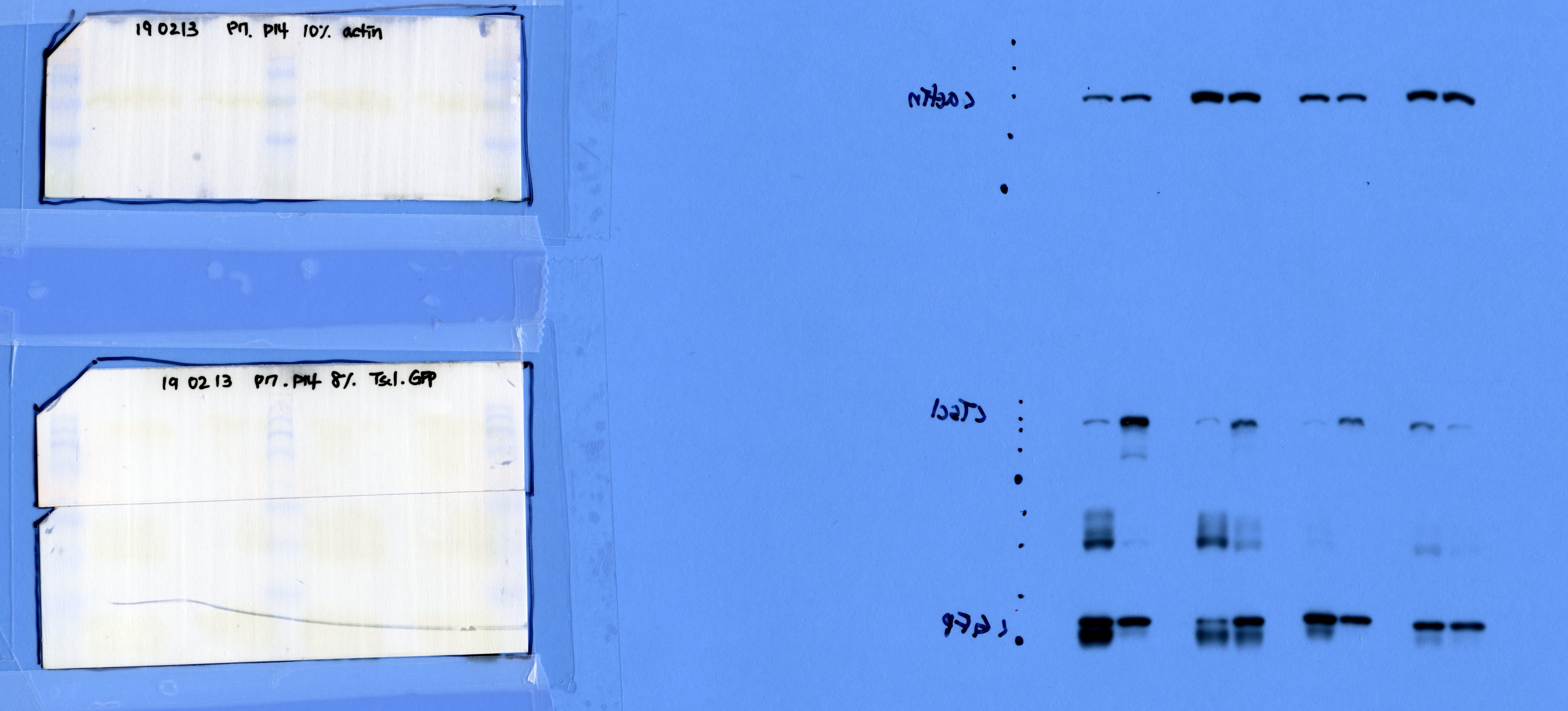

Supplement: Source data 4. [file elife-70079-supp4.zip › Source Data 4_Uncropped images for figure supplements/Figure S2B_actin-1-p7(RPE,Retina)-P14(RPE).jpg]

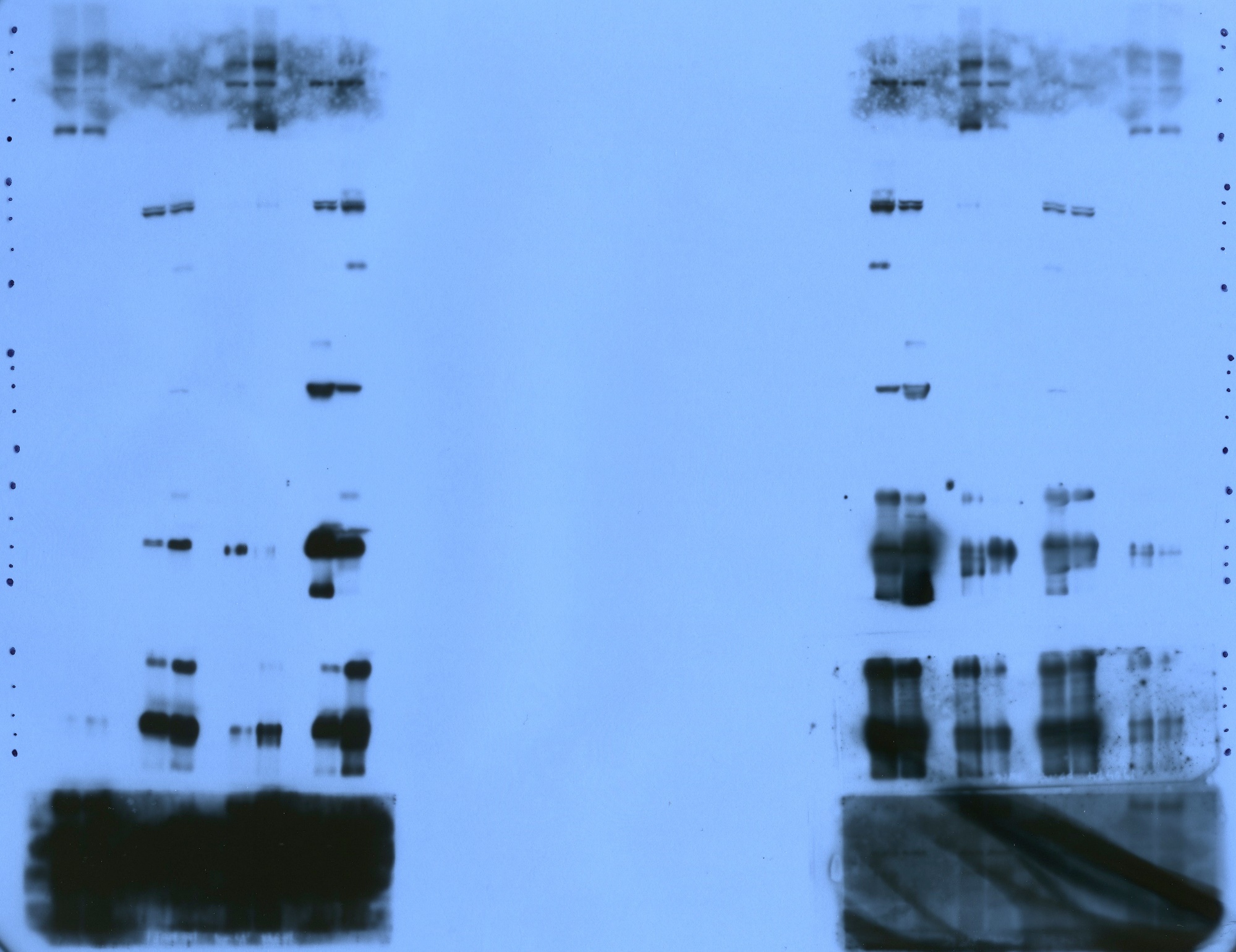

Supplement: Source data 4. [file elife-70079-supp4.zip › Source Data 4_Uncropped images for figure supplements/Figure S2B_GS-p14(RPE, Retina).jpg]

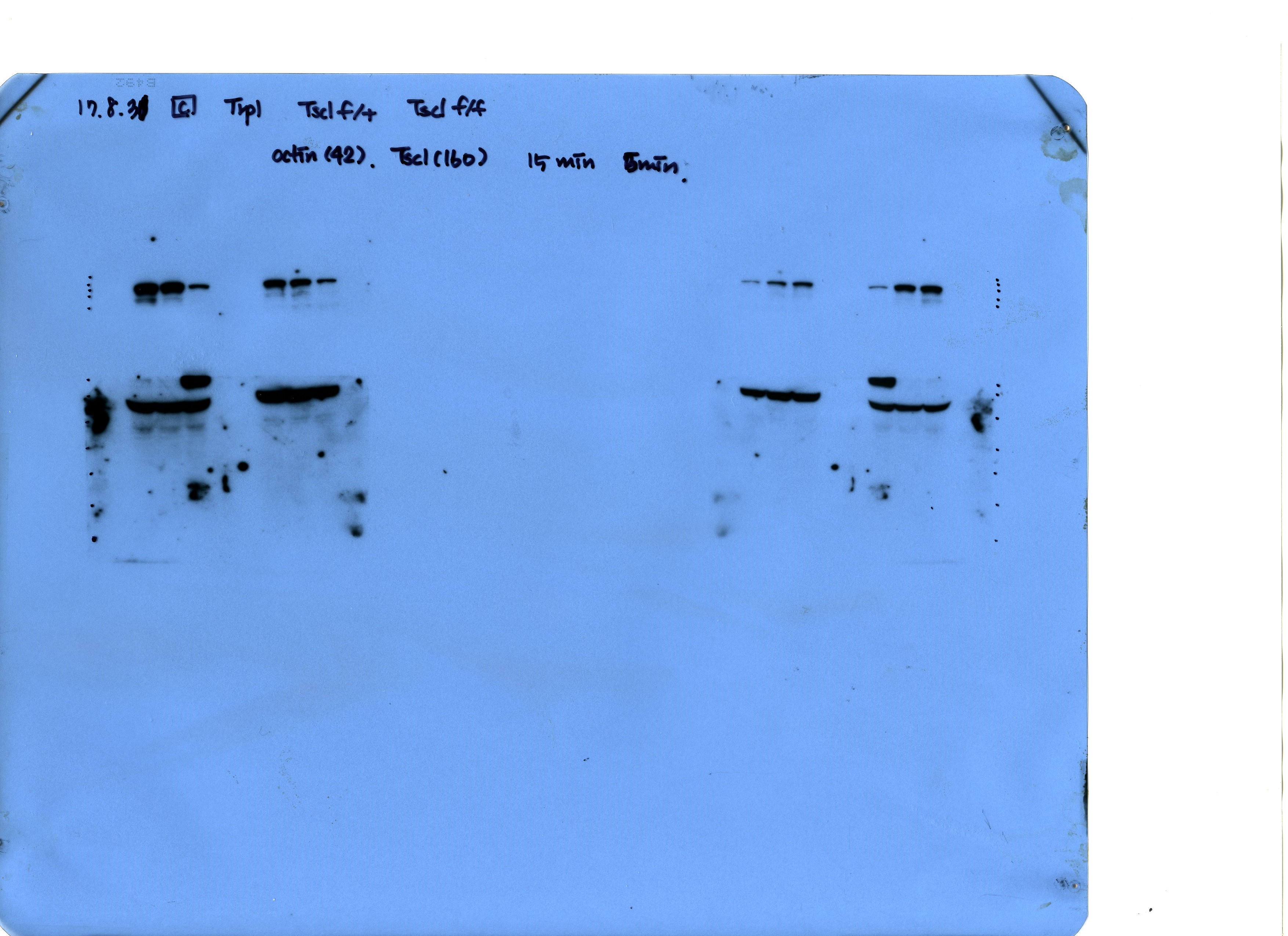

Supplement: Source data 4. [file elife-70079-supp4.zip › Source Data 4_Uncropped images for figure supplements/Figure S2B_TSC1-p30(Retina).jpg]

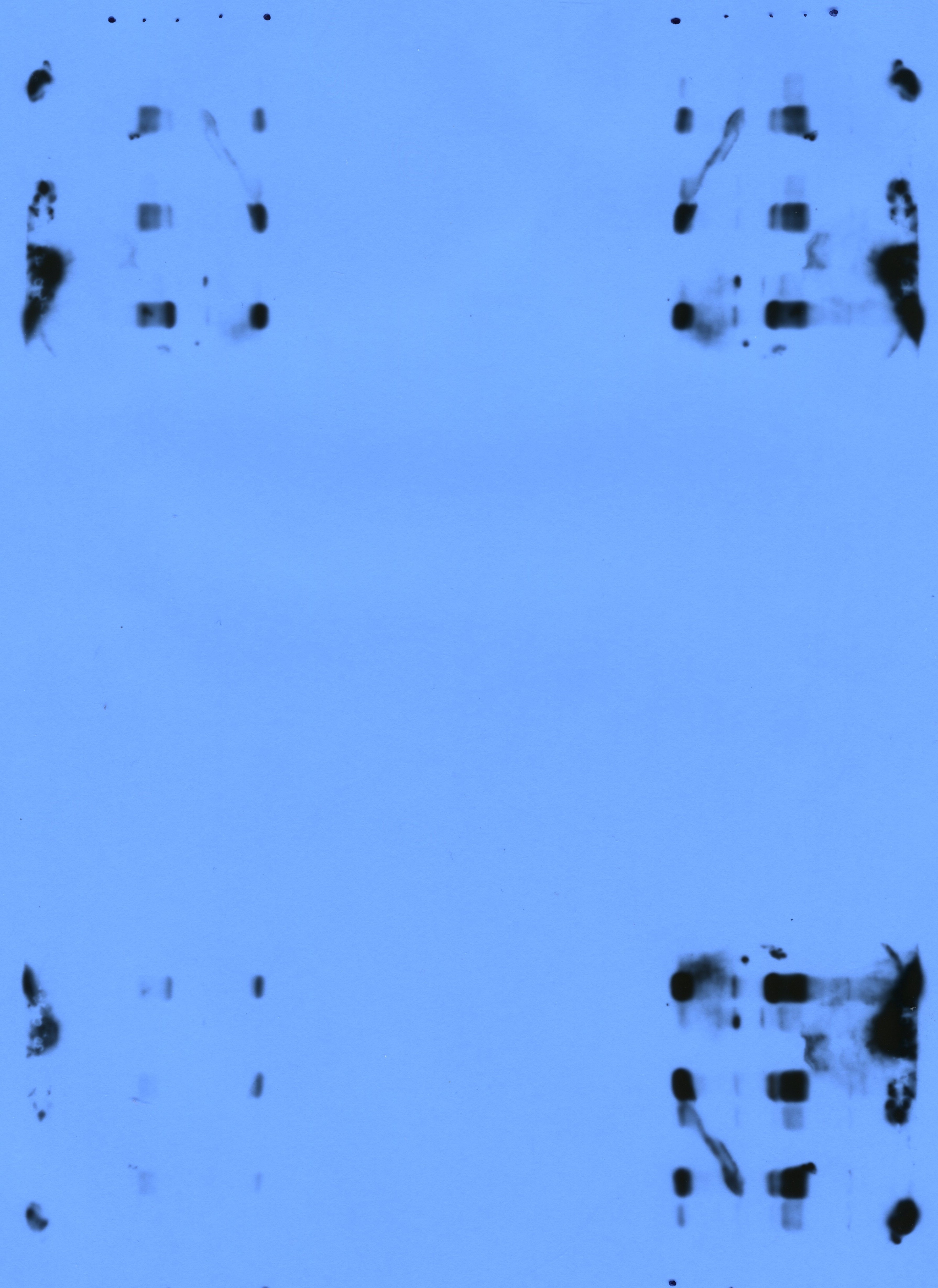

Supplement: Source data 4. [file elife-70079-supp4.zip › Source Data 4_Uncropped images for figure supplements/Figure S8_p7-14-30-cMyc.jpg]

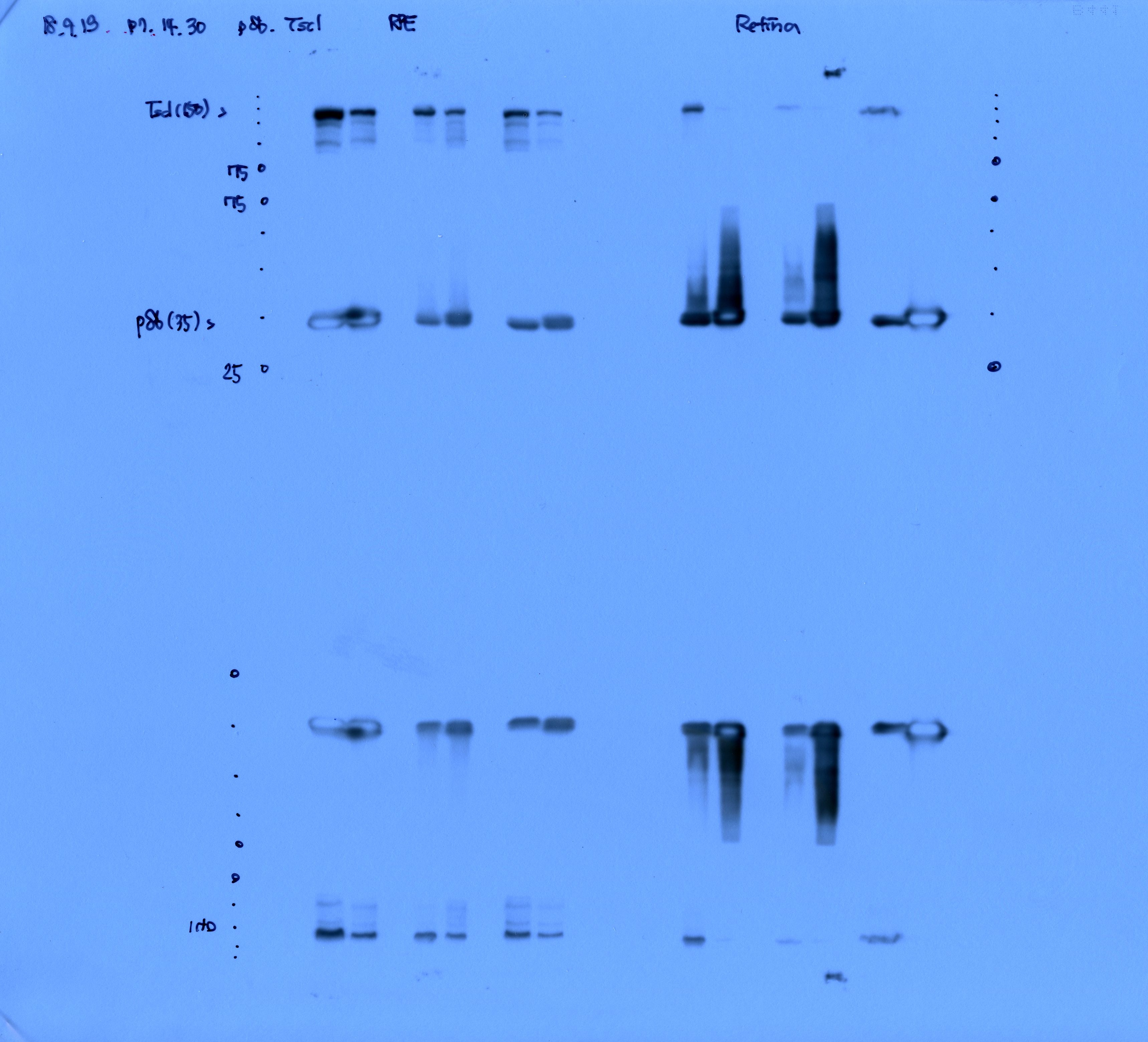

Supplement: Source data 4. [file elife-70079-supp4.zip › Source Data 4_Uncropped images for figure supplements/Figure S2B_pS6-p14(RPE,Retina)-p30(RPE).jpg]

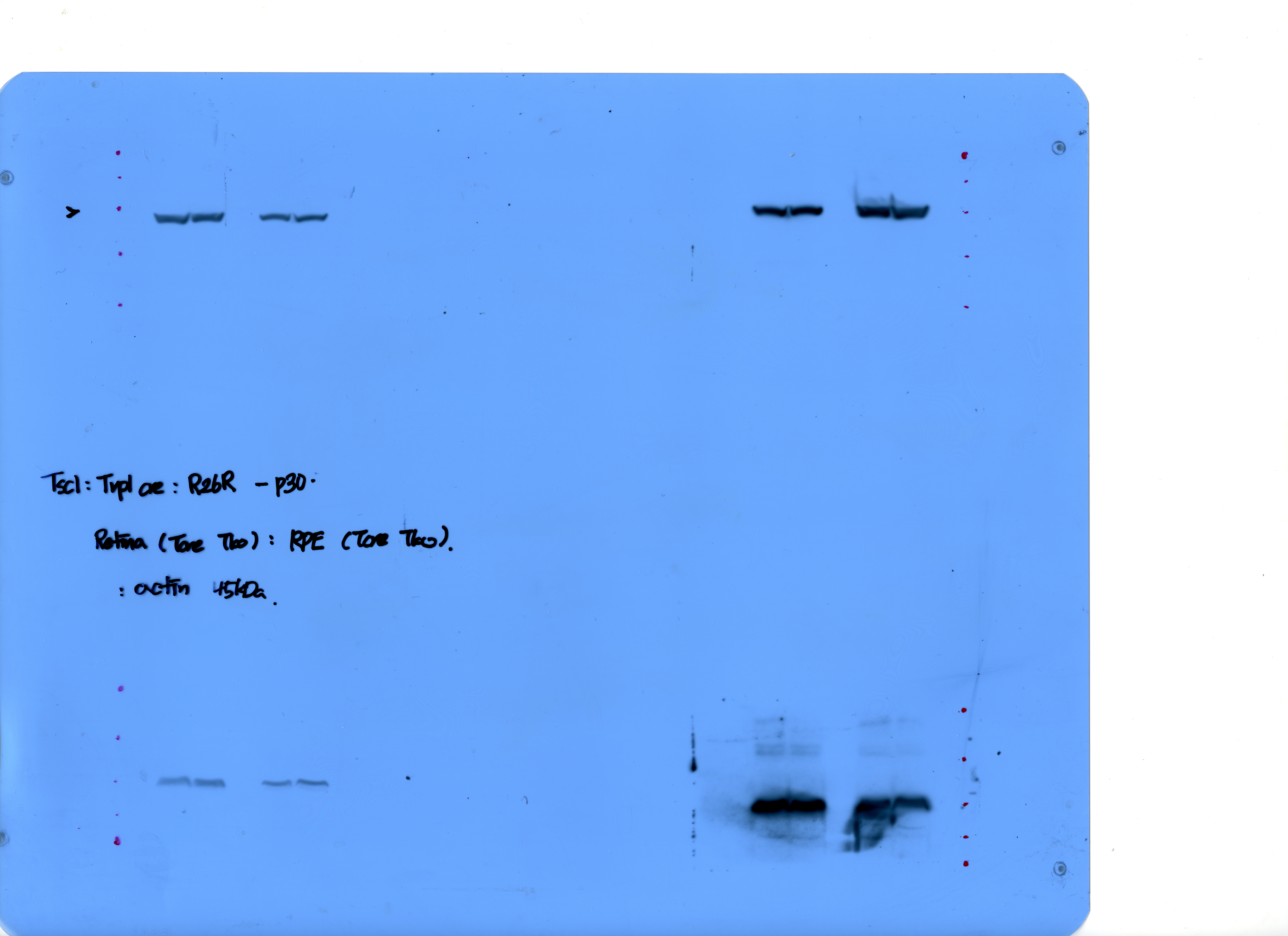

Supplement: Source data 4. [file elife-70079-supp4.zip › Source Data 4_Uncropped images for figure supplements/Figure S2B_actin-p30(RPE, Retina).jpg]

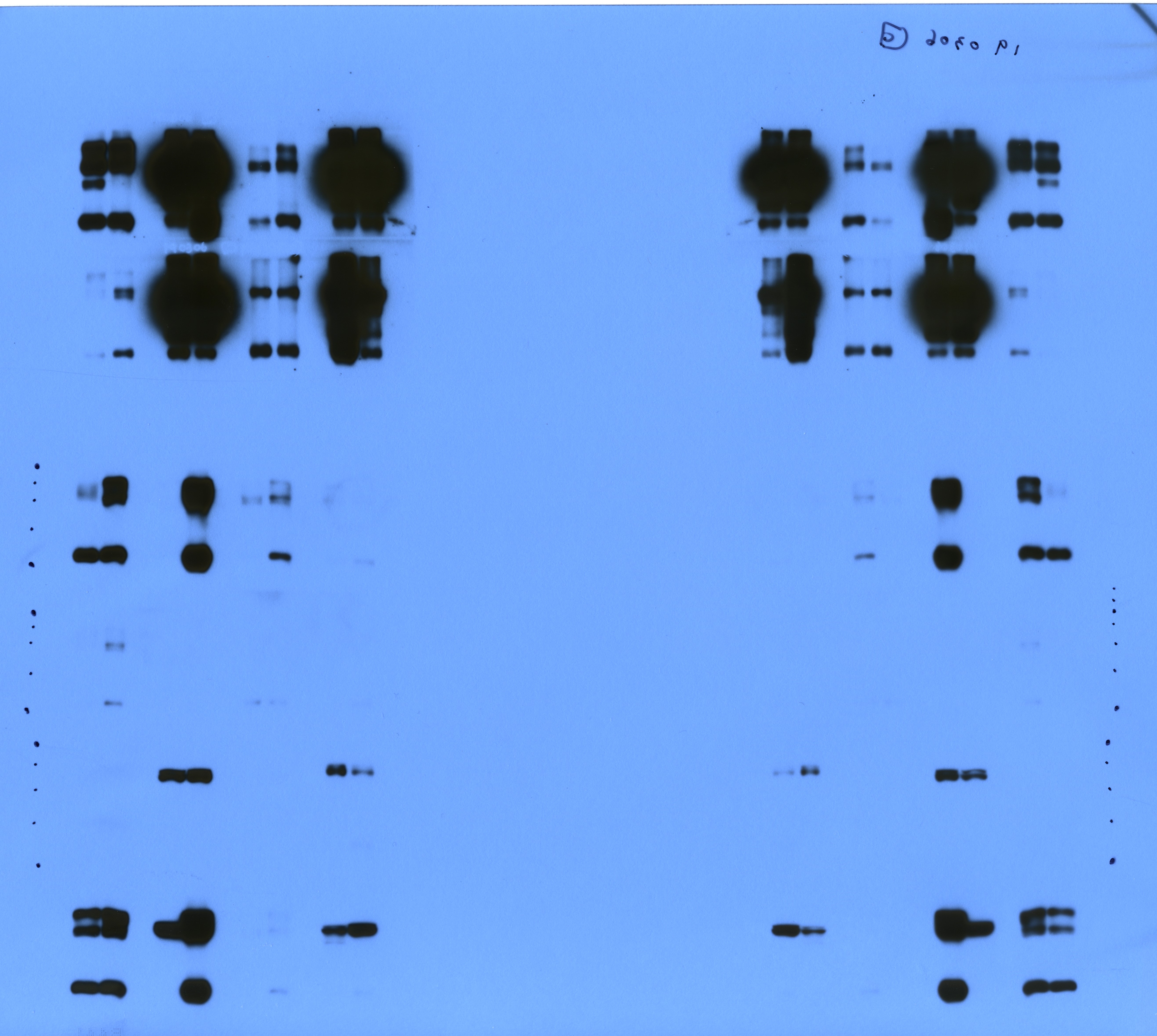

Supplement: Source data 4. [file elife-70079-supp4.zip › Source Data 4_Uncropped images for figure supplements/Figure S2B_RPE65-p30(RPE, Retina).jpg]

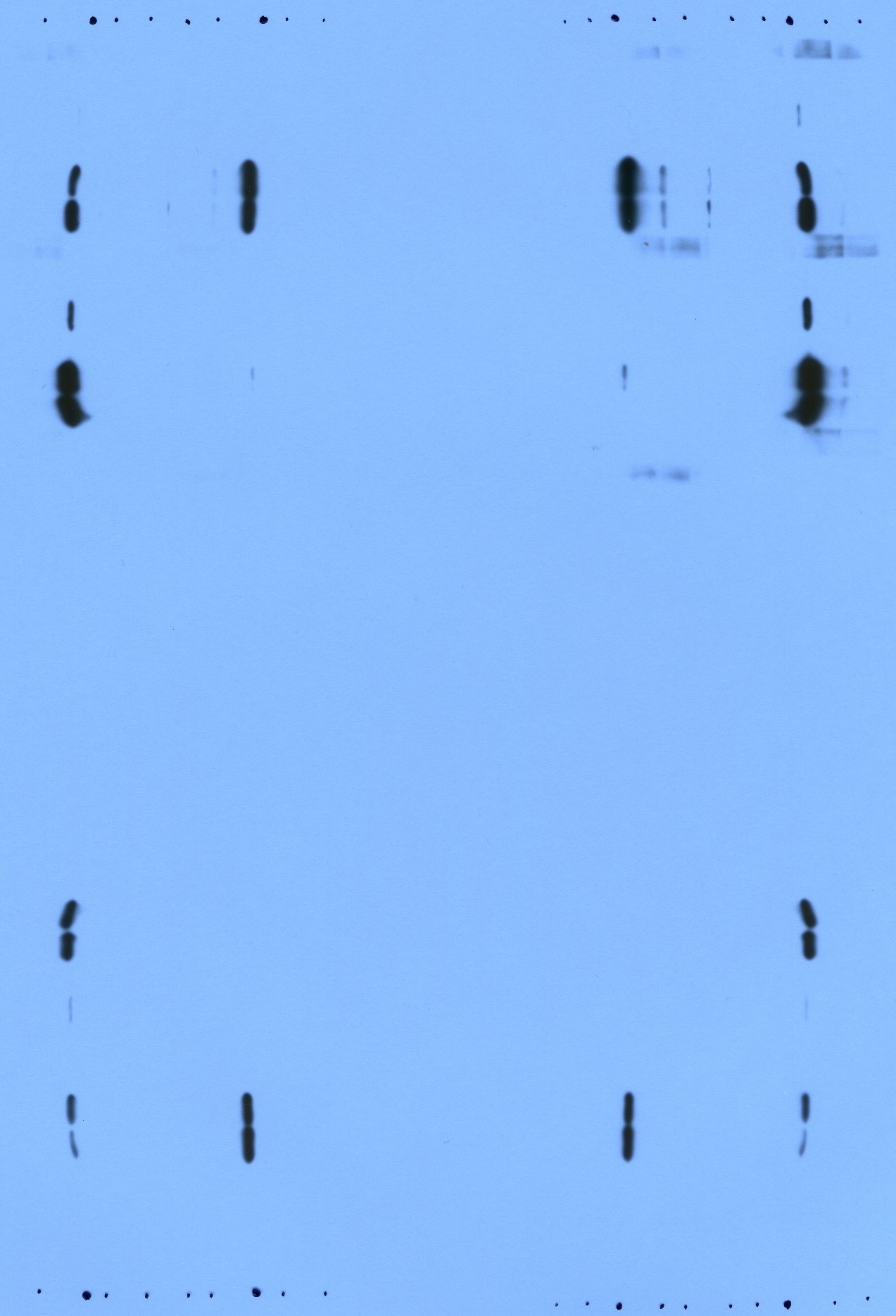

Supplement: Source data 4. [file elife-70079-supp4.zip › Source Data 4_Uncropped images for figure supplements/Figure S2B_Recoverin-p30(RPE, Retina).jpg]

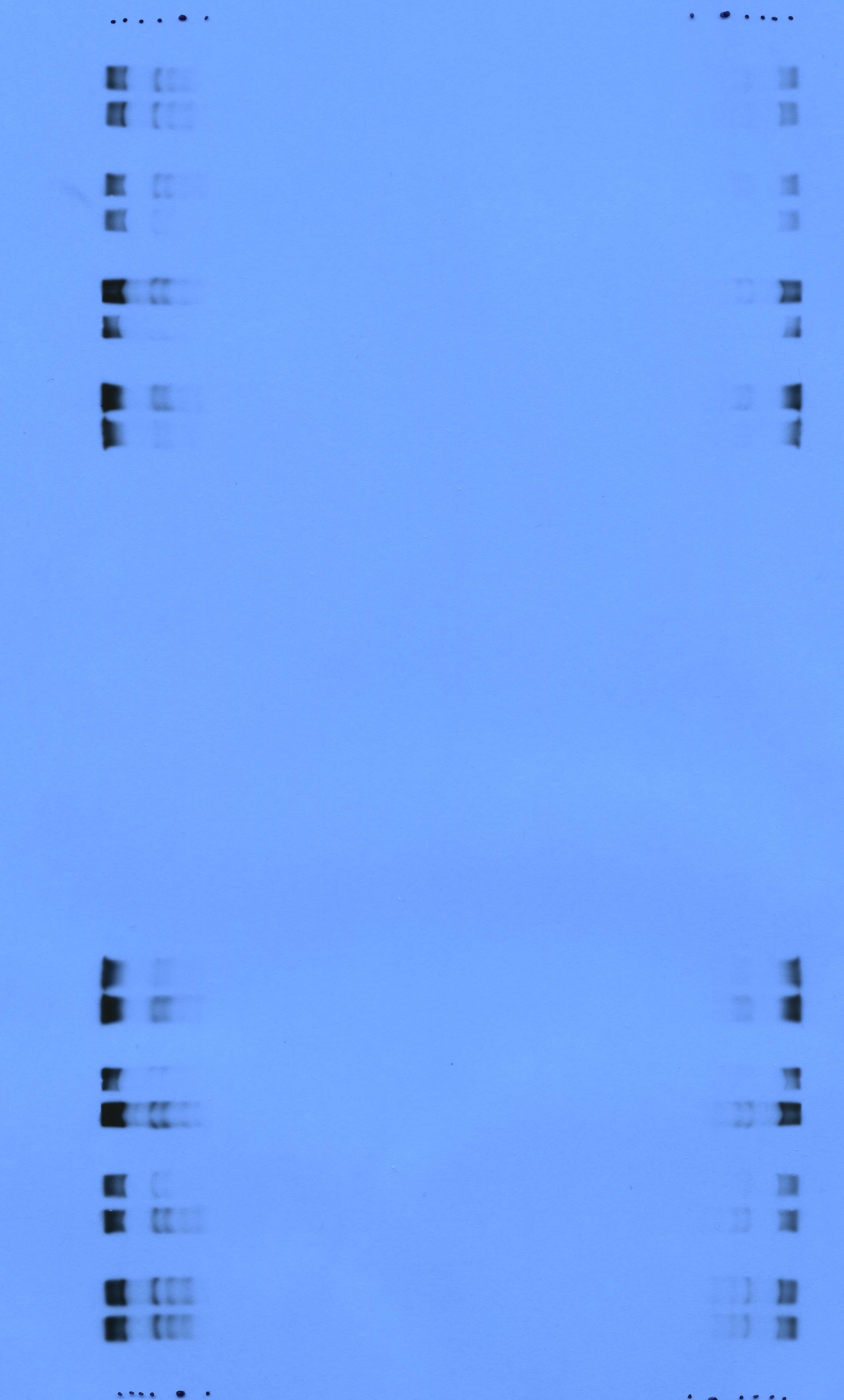

Supplement: Source data 4. [file elife-70079-supp4.zip › Source Data 4_Uncropped images for figure supplements/Figure S4D_Tsc2.jpg]

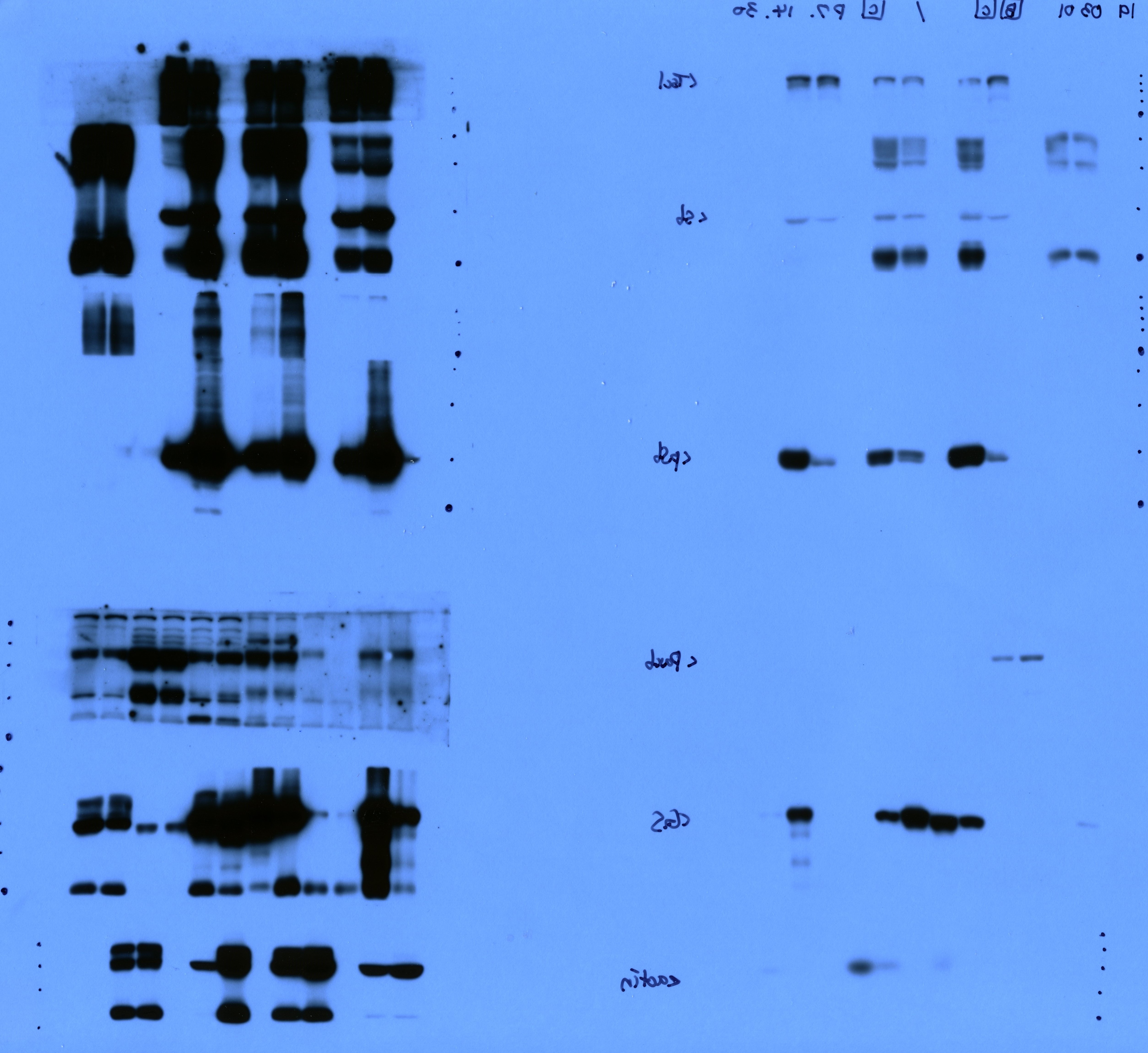

Supplement: Source data 4. [file elife-70079-supp4.zip › Source Data 4_Uncropped images for figure supplements/Figure S4D_pS6-S6-Tsc1.jpg]

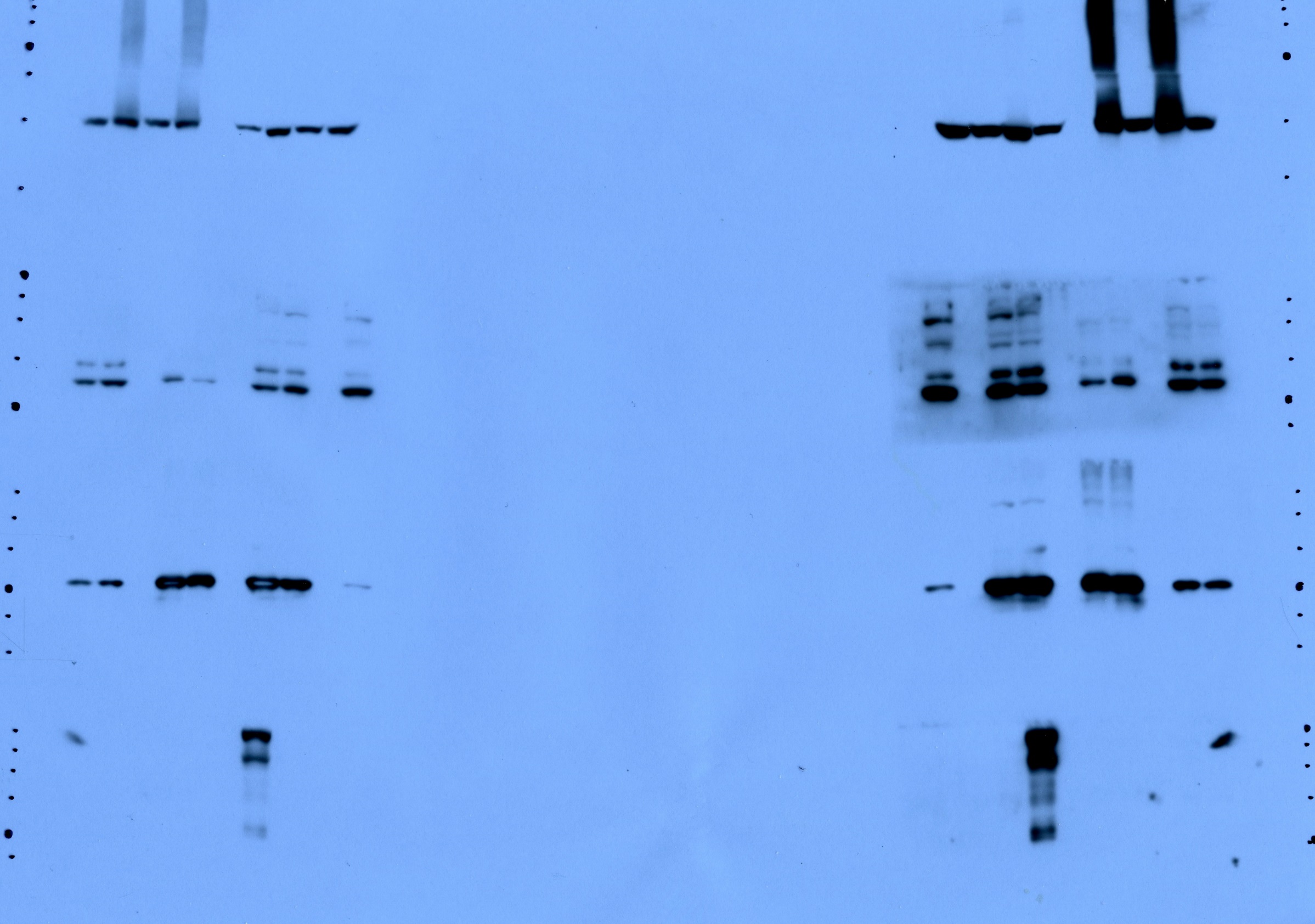

Supplement: Source data 4. [file elife-70079-supp4.zip › Source Data 4_Uncropped images for figure supplements/Figure S2B_Recoverin-p7(Retina).jpg]

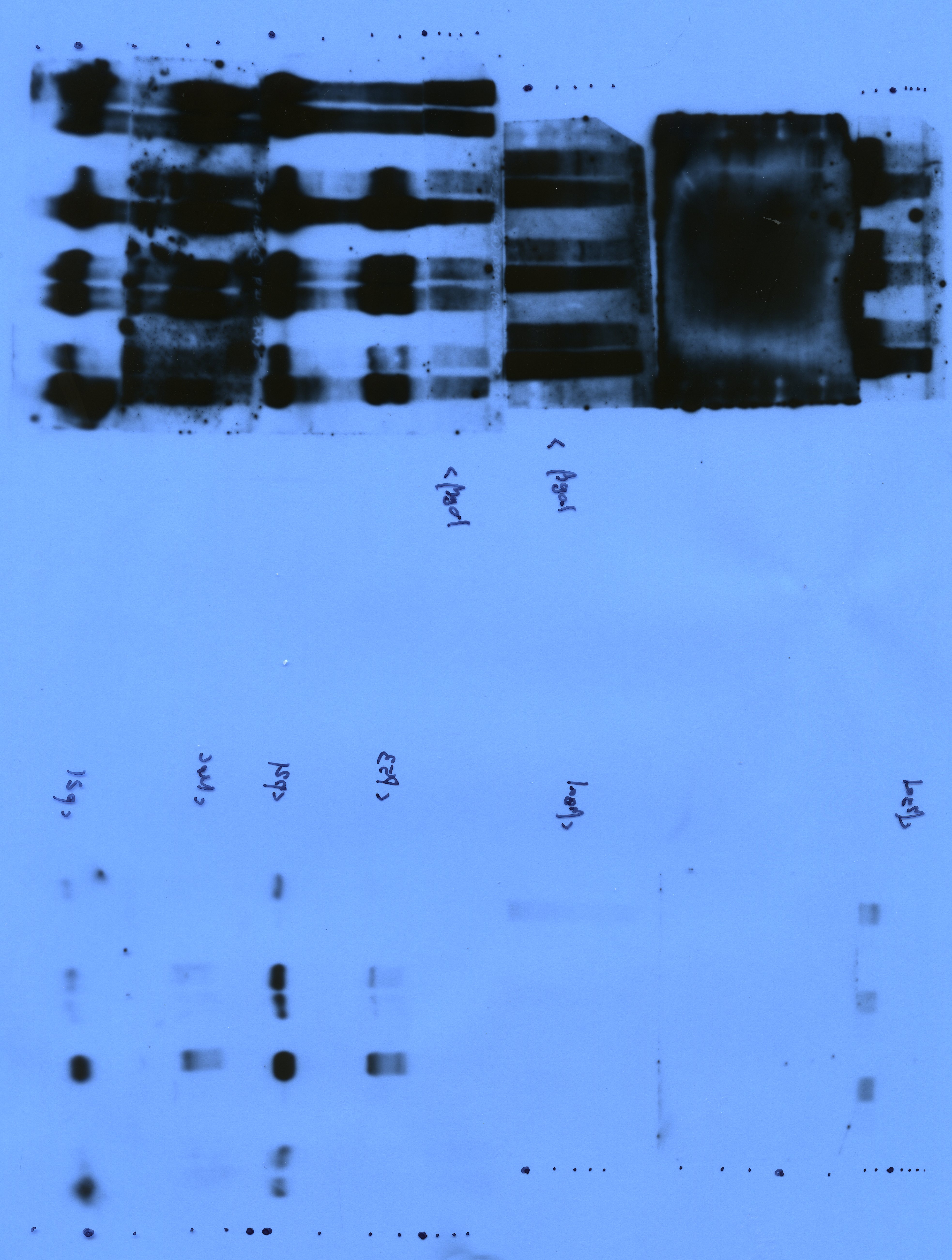

Supplement: Source data 4. [file elife-70079-supp4.zip › Source Data 4_Uncropped images for figure supplements/Figure S8_Tsc2ko-P30-p21-cMyc-.jpg]

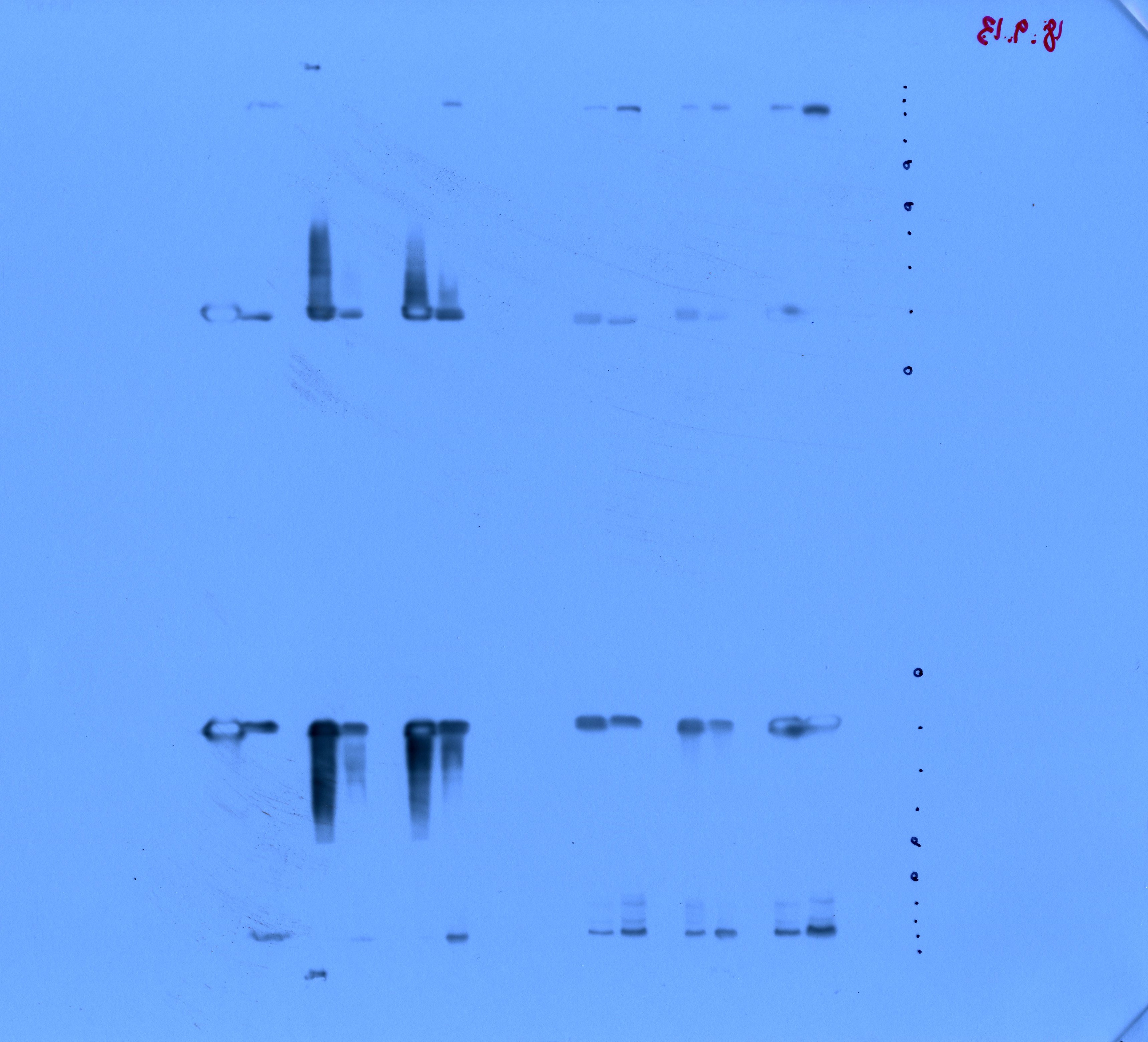

Supplement: Source data 4. [file elife-70079-supp4.zip › Source Data 4_Uncropped images for figure supplements/Figure S2B_TSC1-p7(RPE).jpg]

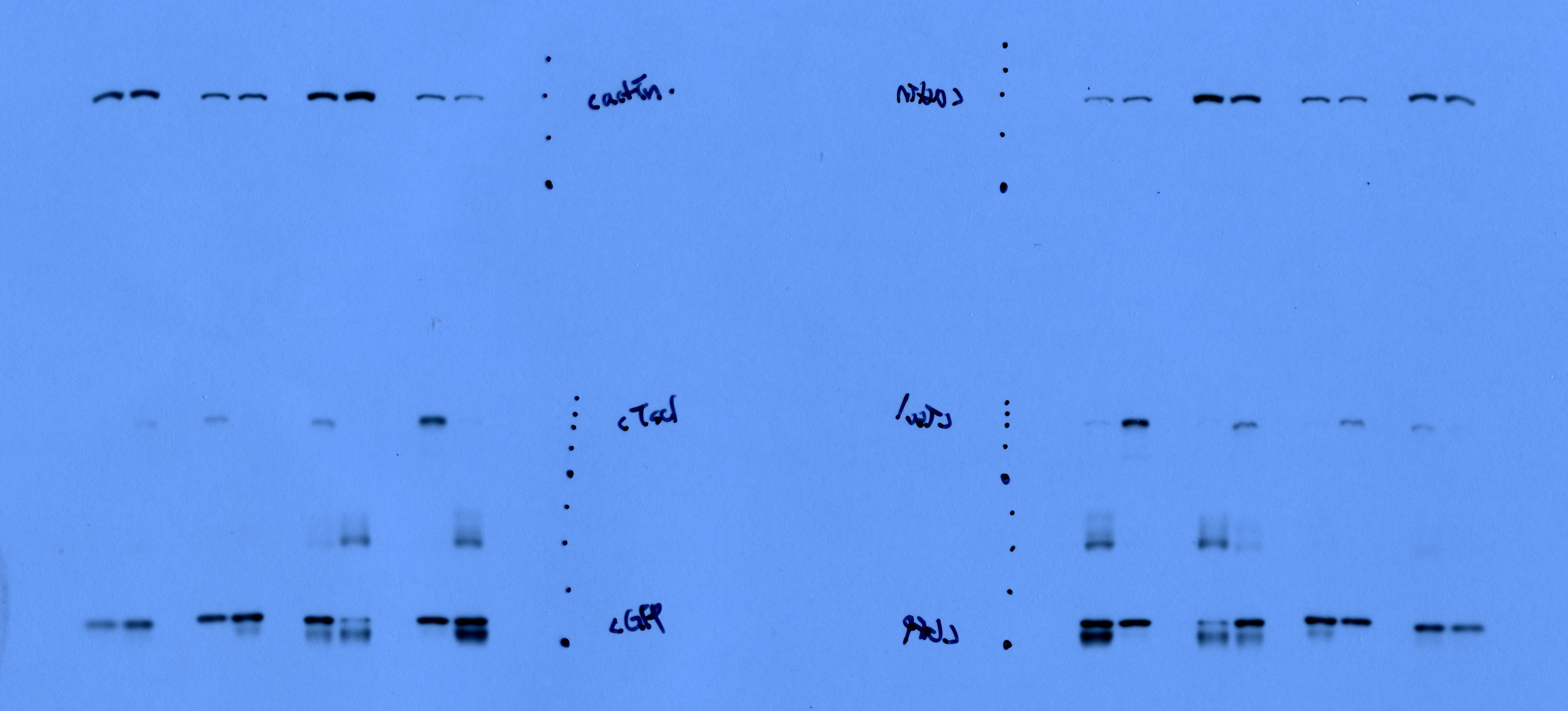

Supplement: Source data 4. [file elife-70079-supp4.zip › Source Data 4_Uncropped images for figure supplements/Figure S2B_TSC1-p14(RPE, Retina).jpg]

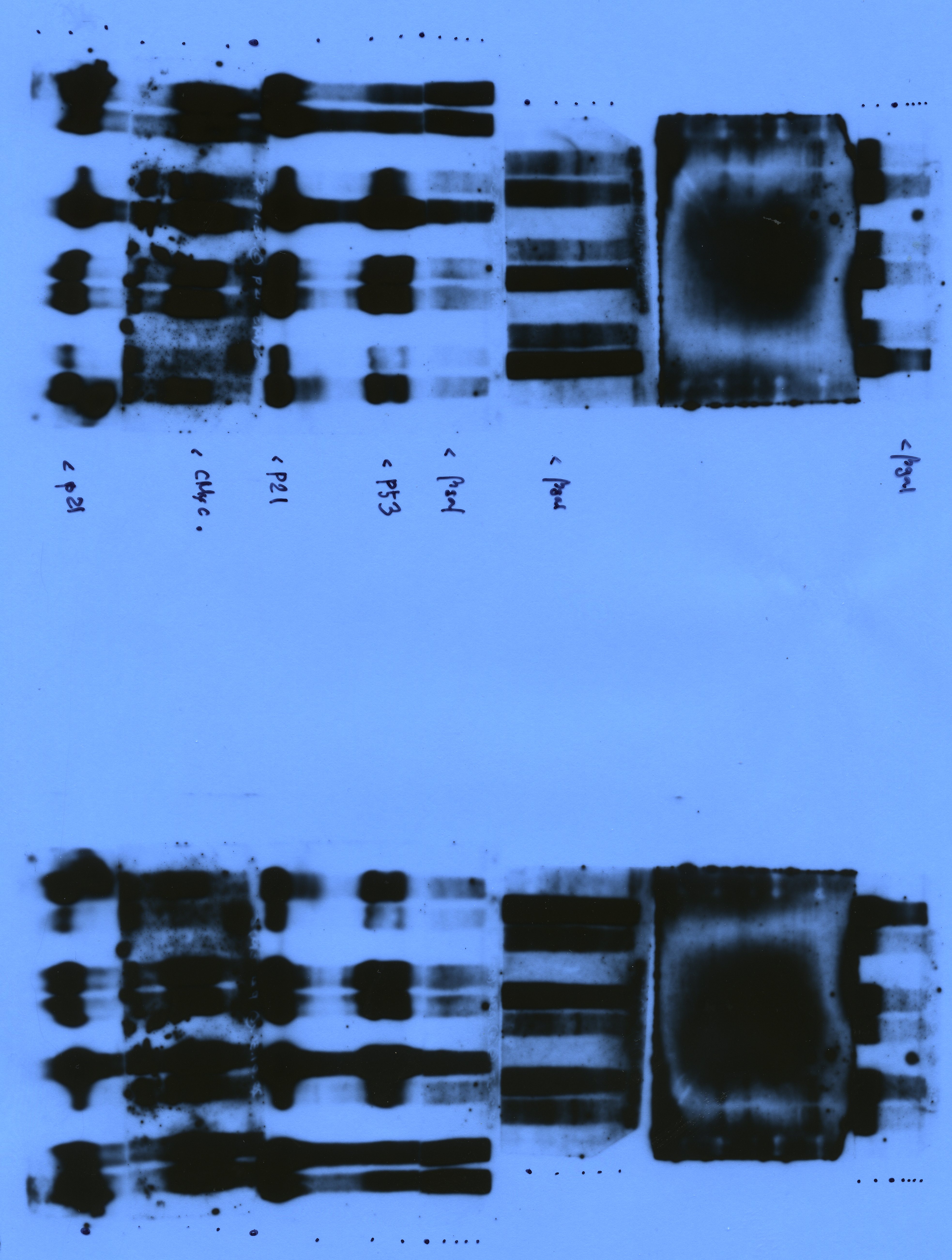

Supplement: Source data 4. [file elife-70079-supp4.zip › Source Data 4_Uncropped images for figure supplements/Figure S8_Tsc2ko-P30-betaGal-.jpg]

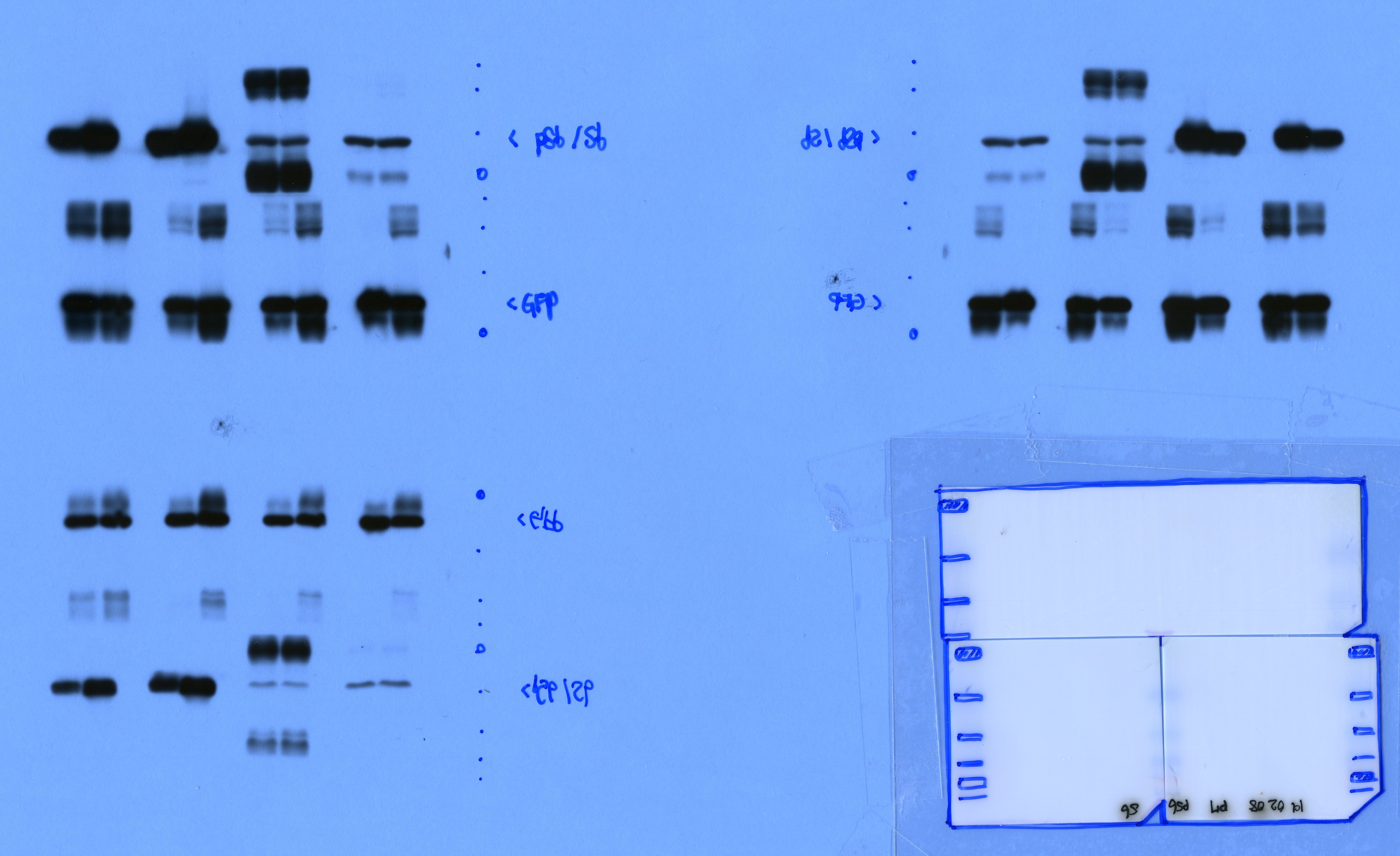

Supplement: Source data 4. [file elife-70079-supp4.zip › Source Data 4_Uncropped images for figure supplements/Figure S2B_pS6-S6-p7(RPE, Retina).jpg]

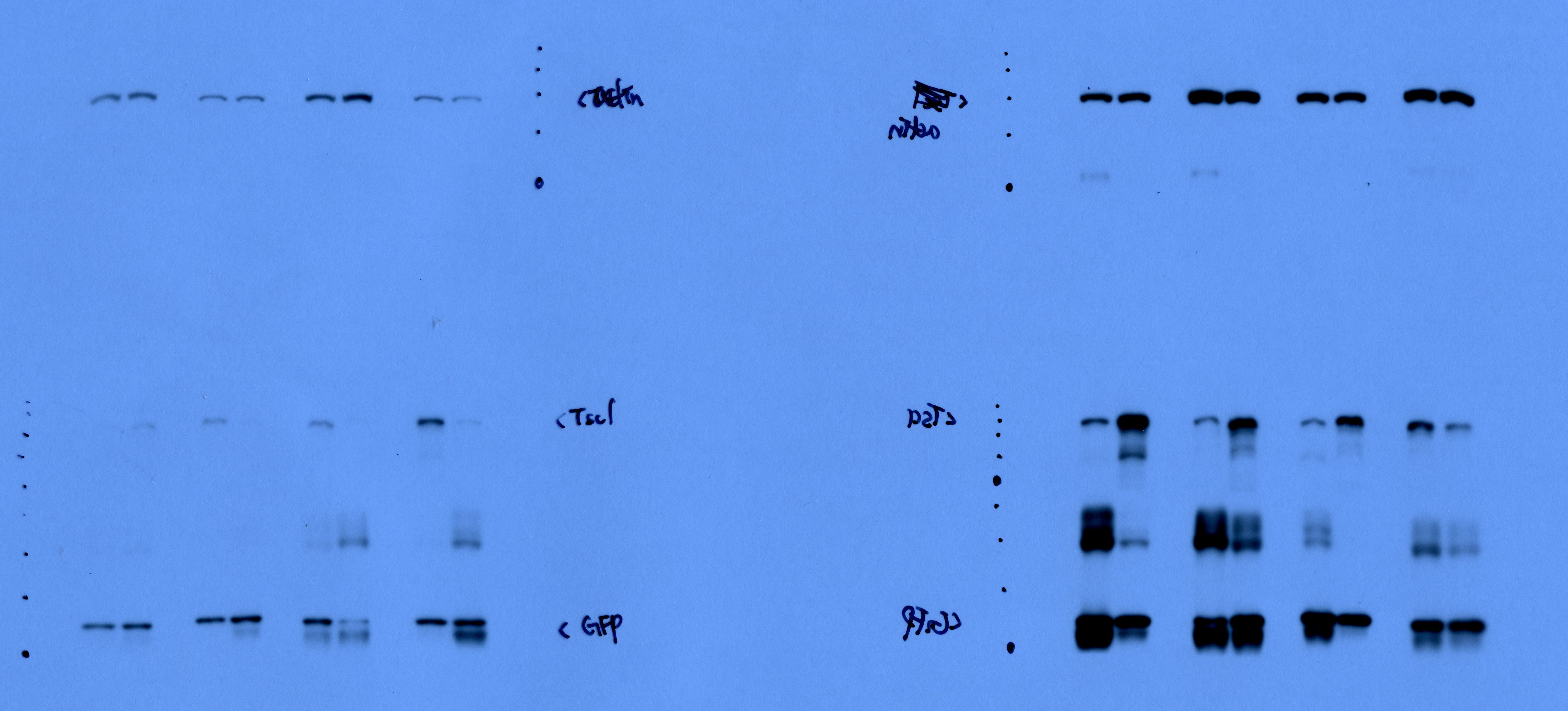

Supplement: Source data 4. [file elife-70079-supp4.zip › Source Data 4_Uncropped images for figure supplements/Figure S2B_actin-2-P14(Retina).jpg]

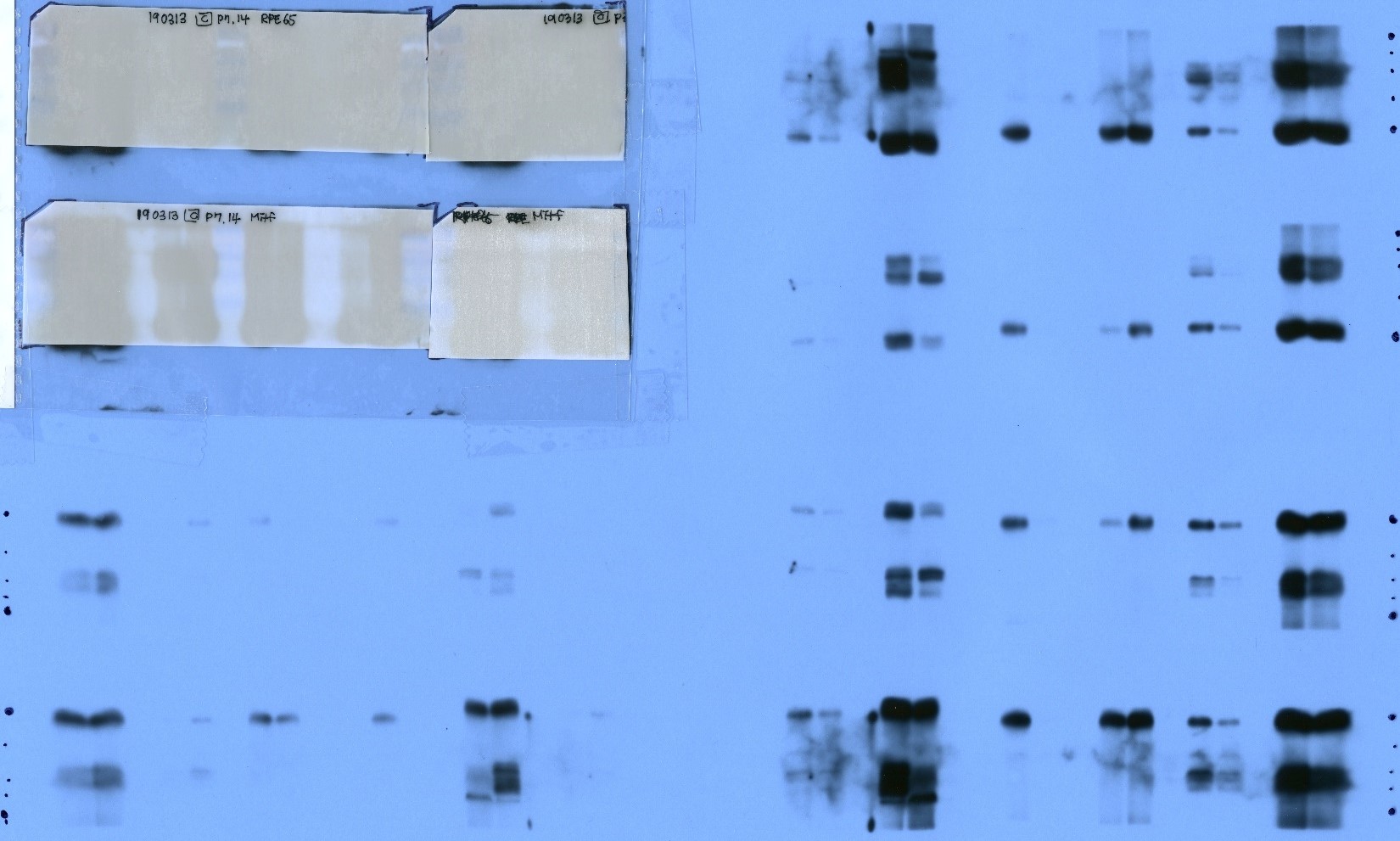

Supplement: Source data 4. [file elife-70079-supp4.zip › Source Data 4_Uncropped images for figure supplements/Figure S2B_RPE65-p7(RPE, Retina).jpg]

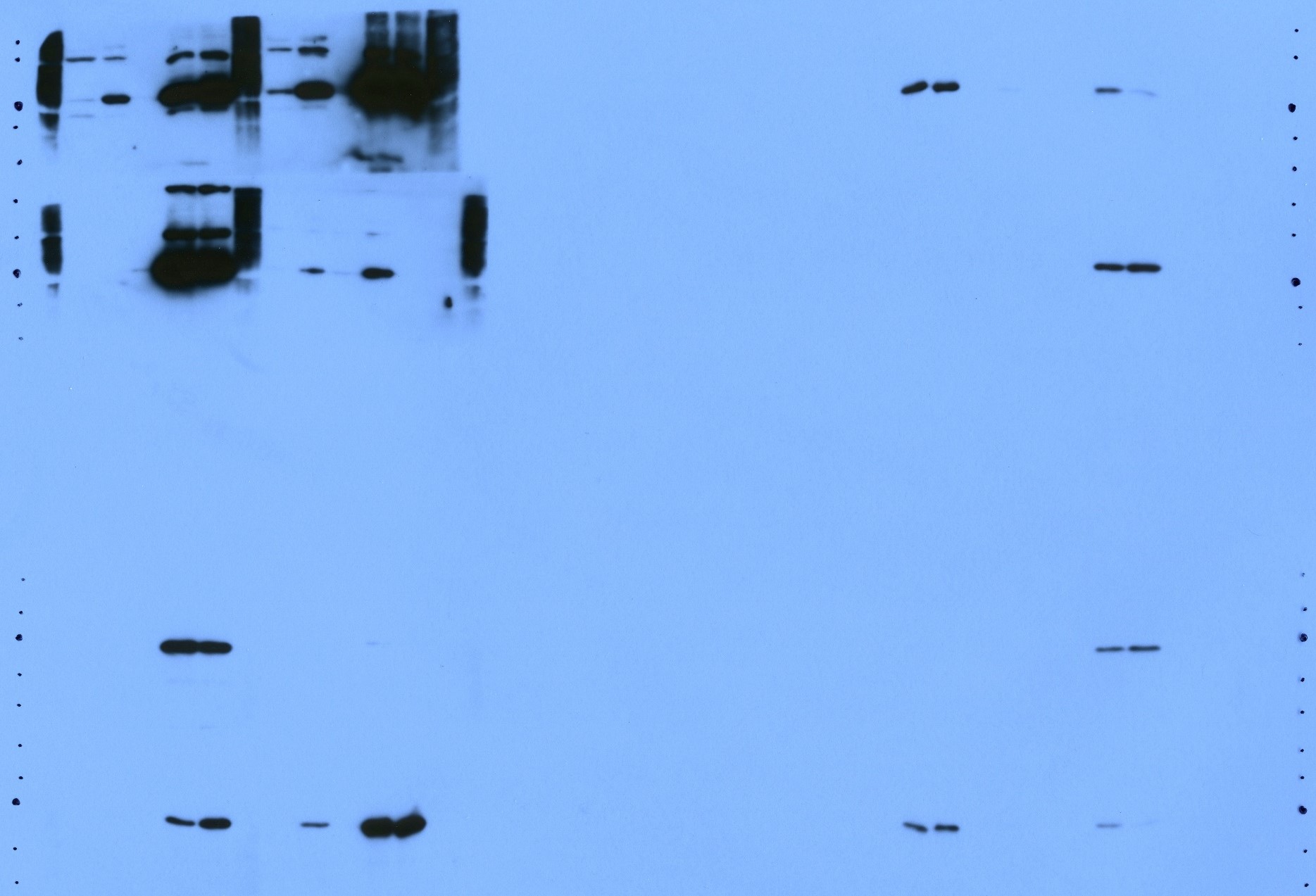

Supplement: Source data 4. [file elife-70079-supp4.zip › Source Data 4_Uncropped images for figure supplements/Figure S2B_Recoverin-p14(RPE, Retina)-P7(REP).jpg]

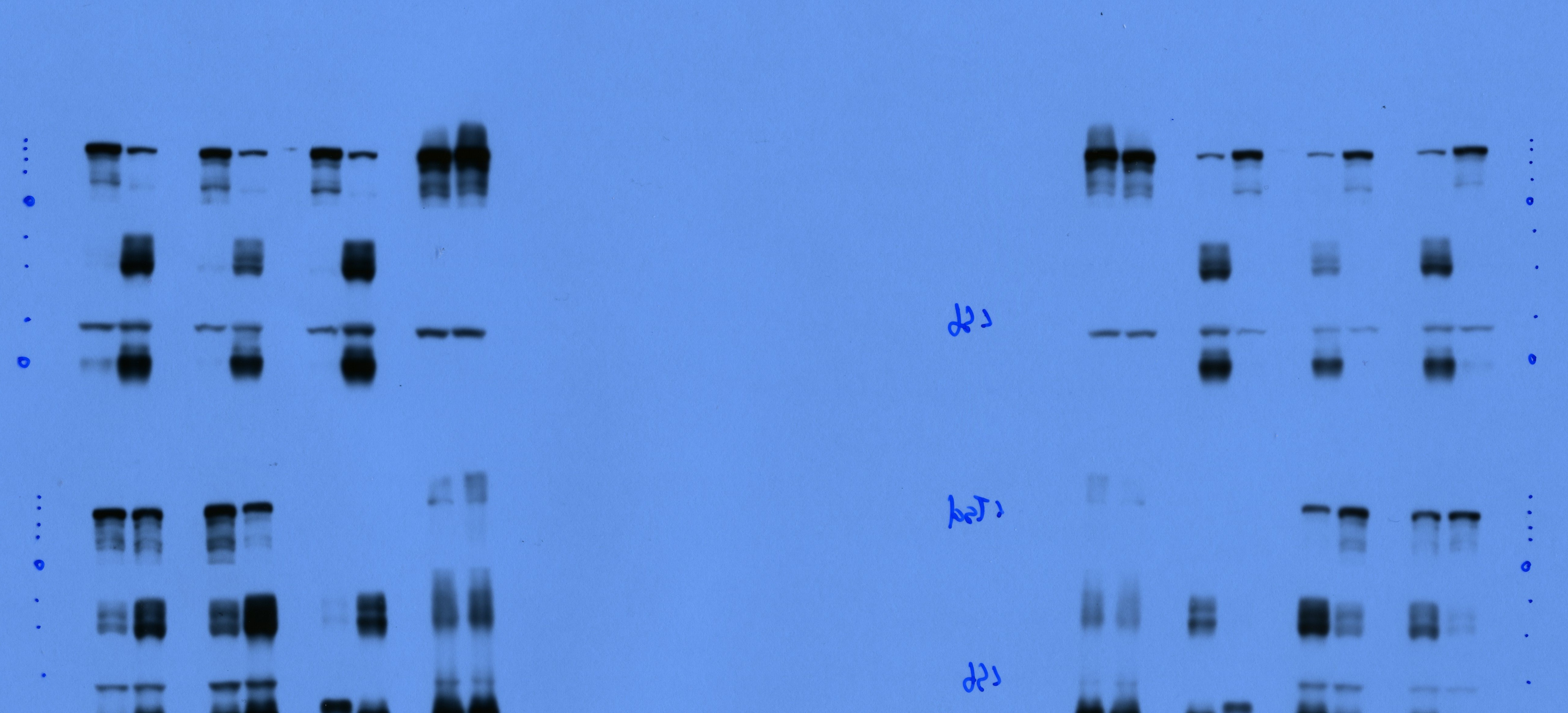

Supplement: Source data 4. [file elife-70079-supp4.zip › Source Data 4_Uncropped images for figure supplements/Figure S2B_S6-p14(RPE, Retina).jpg]

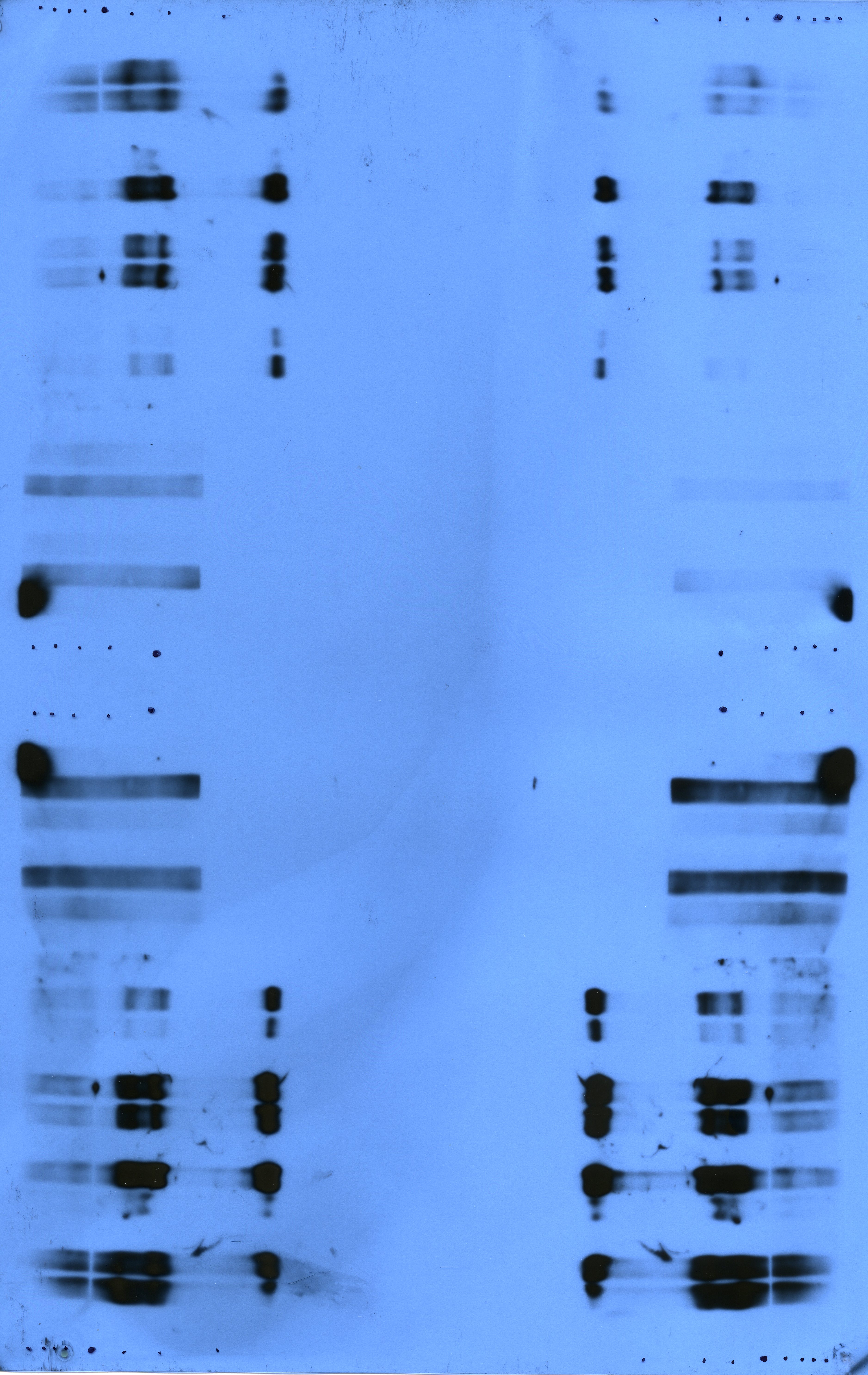

Supplement: Source data 4. [file elife-70079-supp4.zip › Source Data 4_Uncropped images for figure supplements/Figure S8_Tsc2ko-P30-p53.jpg]

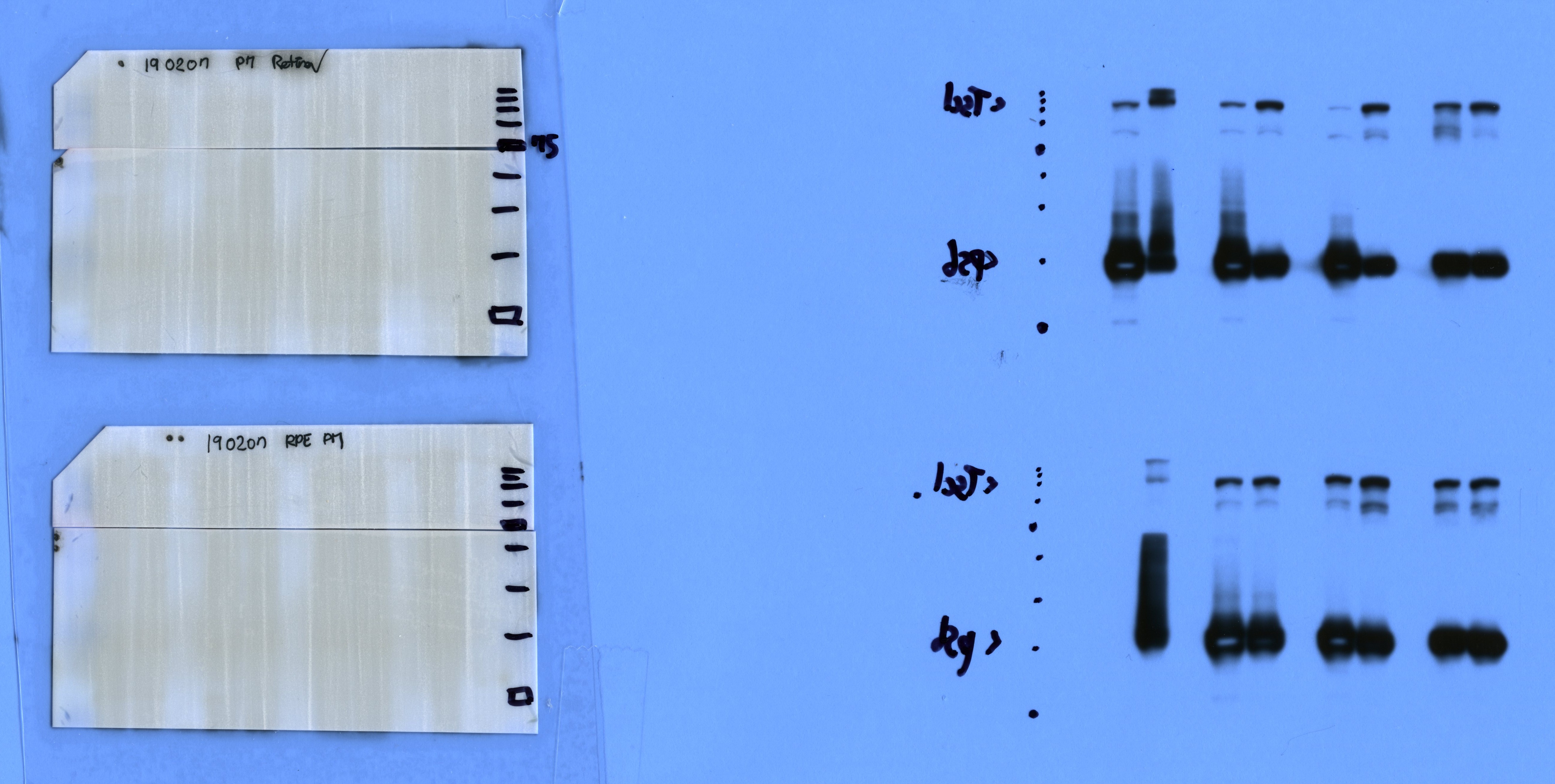

Supplement: Source data 4. [file elife-70079-supp4.zip › Source Data 4_Uncropped images for figure supplements/Figure S2B_TSC1-p7(Retina).jpg]

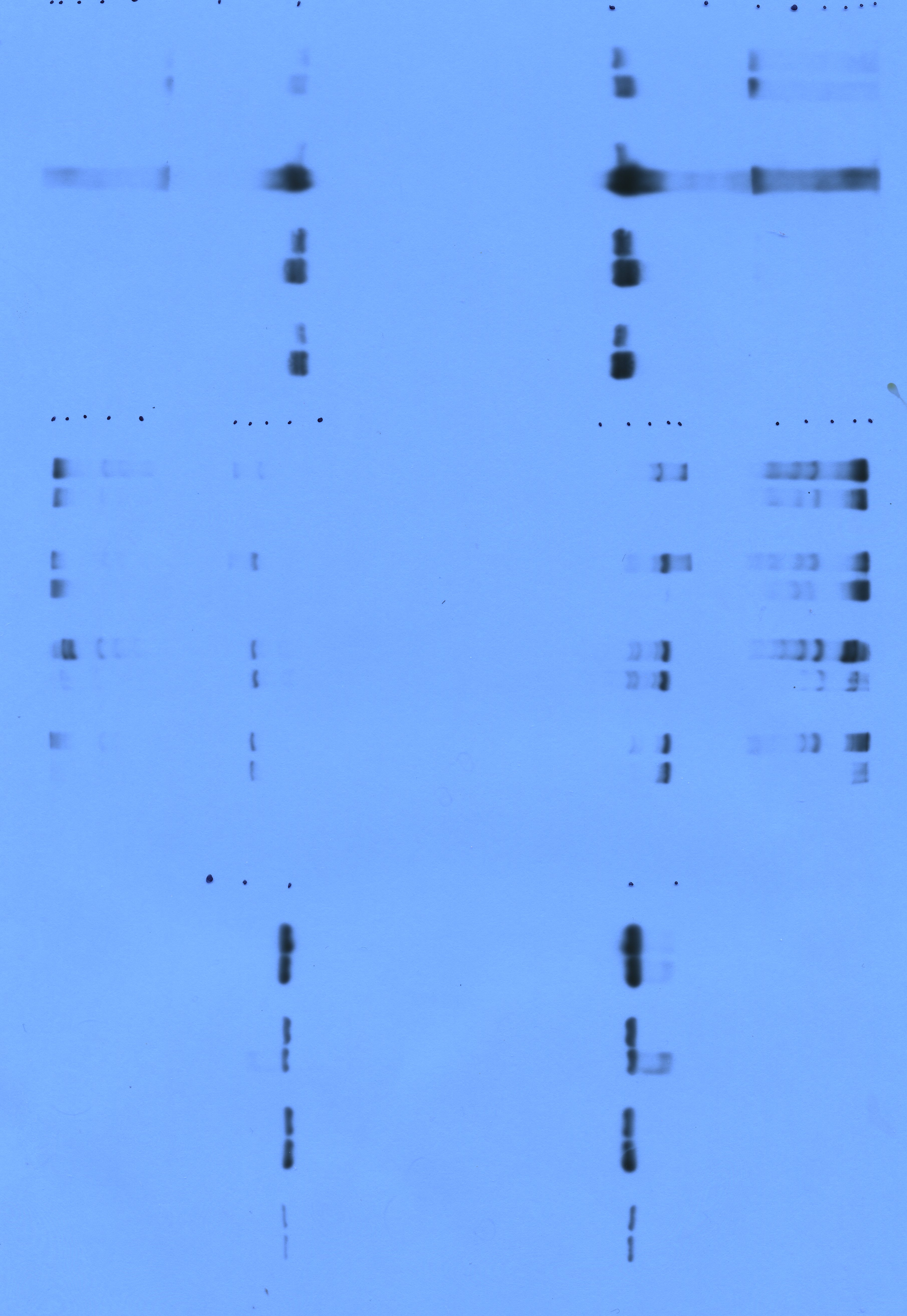

Supplement: Source data 4. [file elife-70079-supp4.zip › Source Data 4_Uncropped images for figure supplements/Figure S8_Tsc2ko-P30-pS6-actin.jpg]

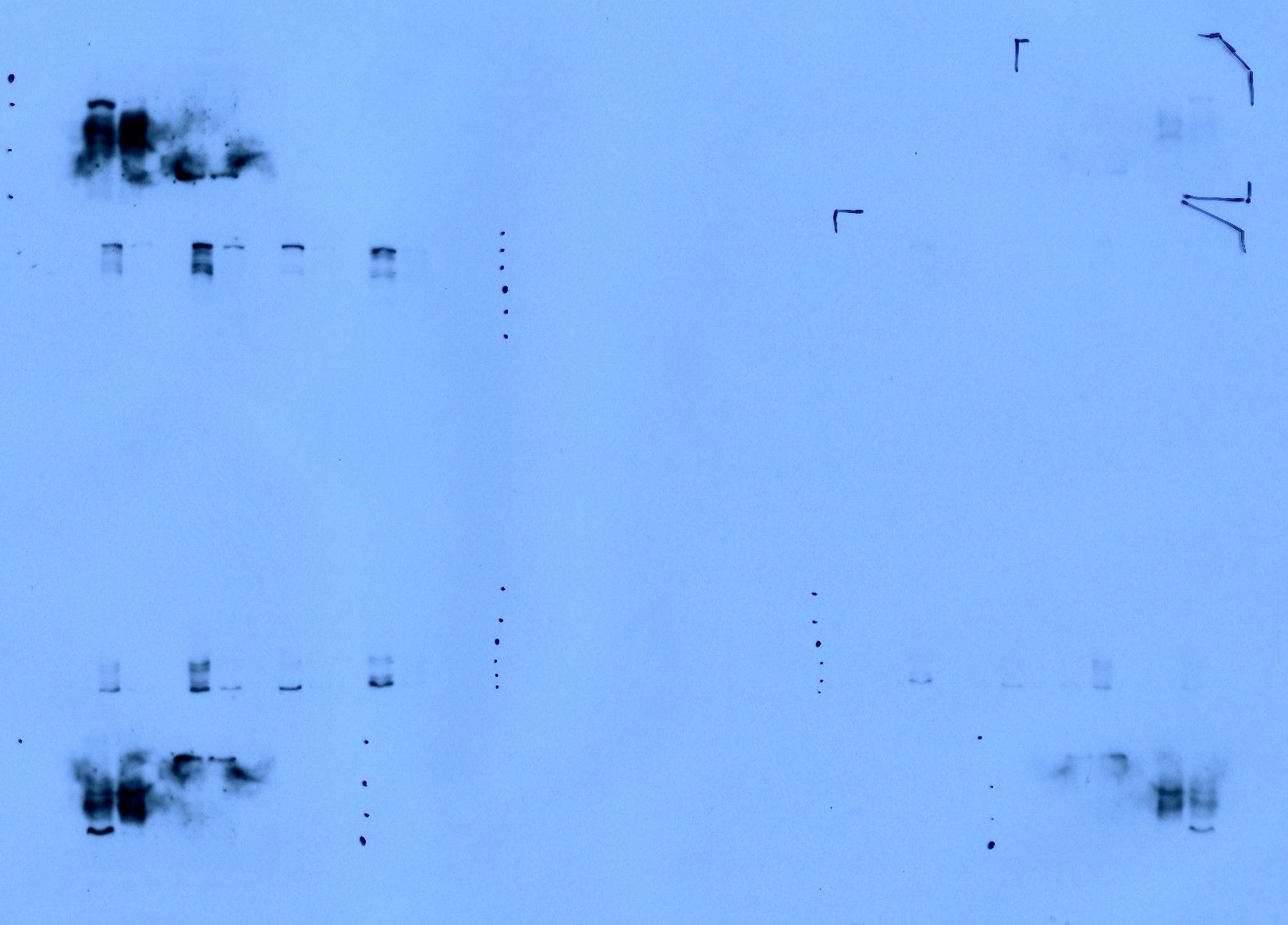

Supplement: Source data 4. [file elife-70079-supp4.zip › Source Data 4_Uncropped images for figure supplements/Figure S2B_RPE65-p14(RPE, Retina).jpg]

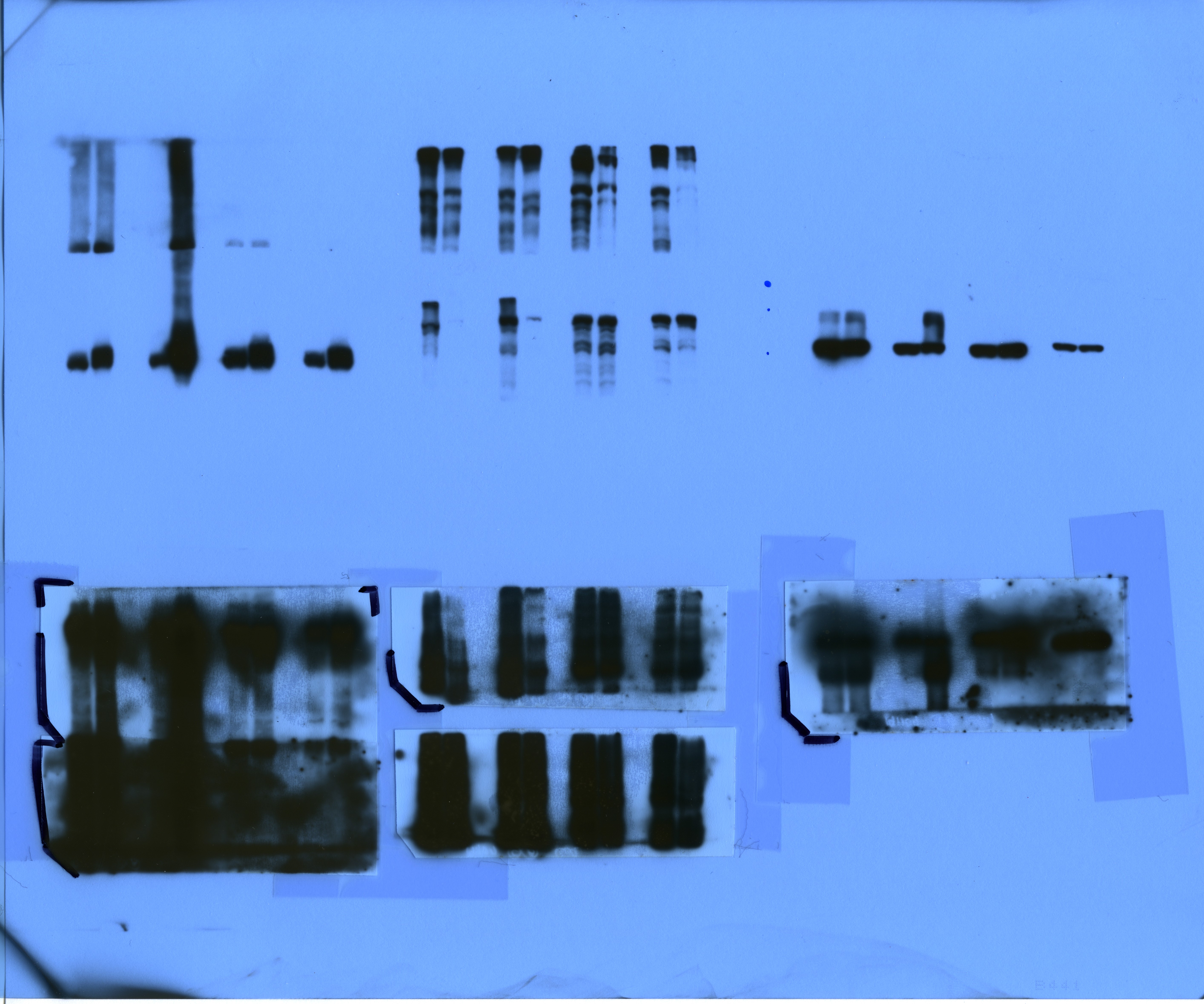

Supplement: Source data 4. [file elife-70079-supp4.zip › Source Data 4_Uncropped images for figure supplements/Figure S4D_actin.jpg]

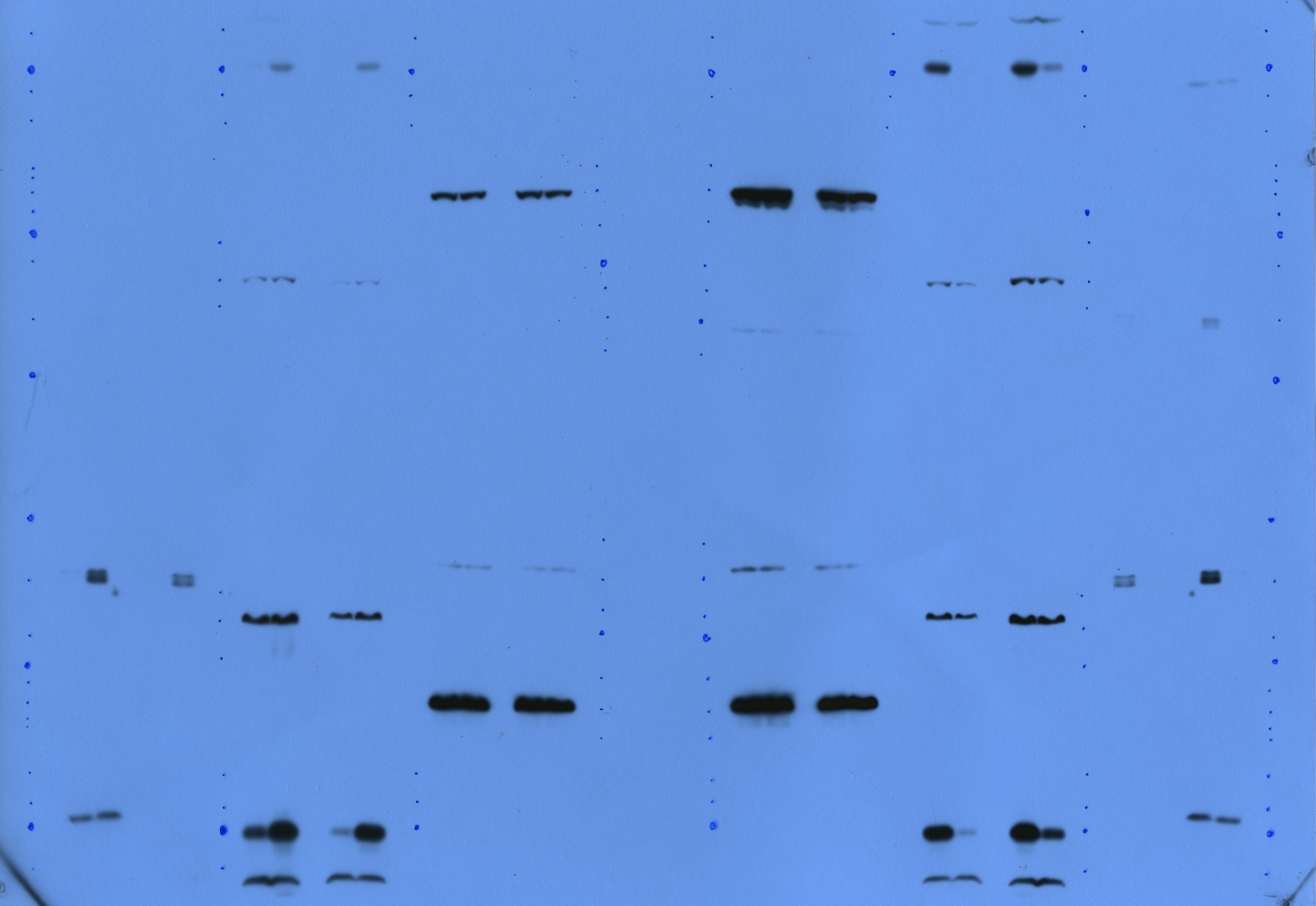

Supplement: Source data 5. [file elife-70079-supp5.zip › Figure S10B_psmb8.jpg]

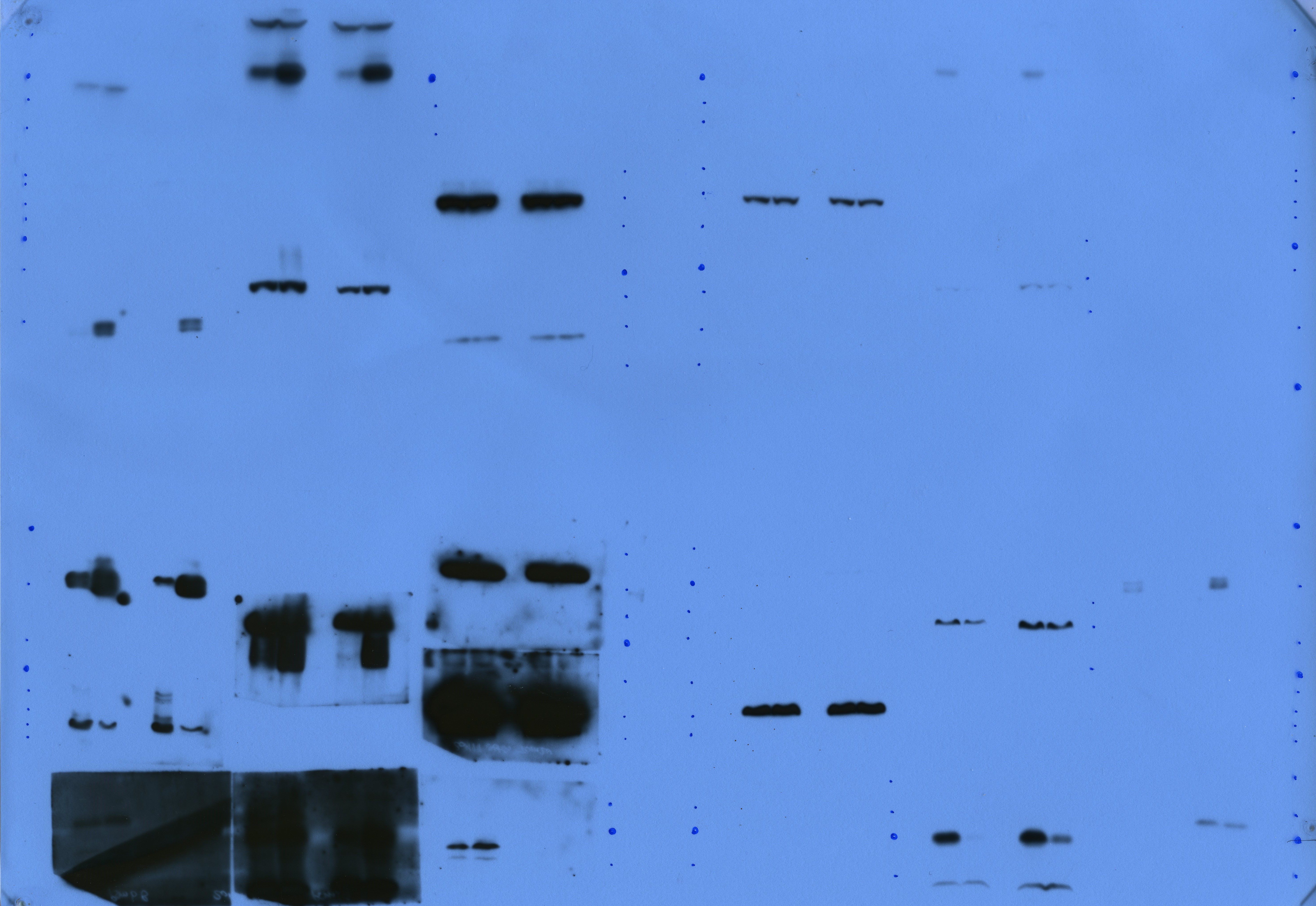

Supplement: Source data 5. [file elife-70079-supp5.zip › Figure S10B_actin.jpg]

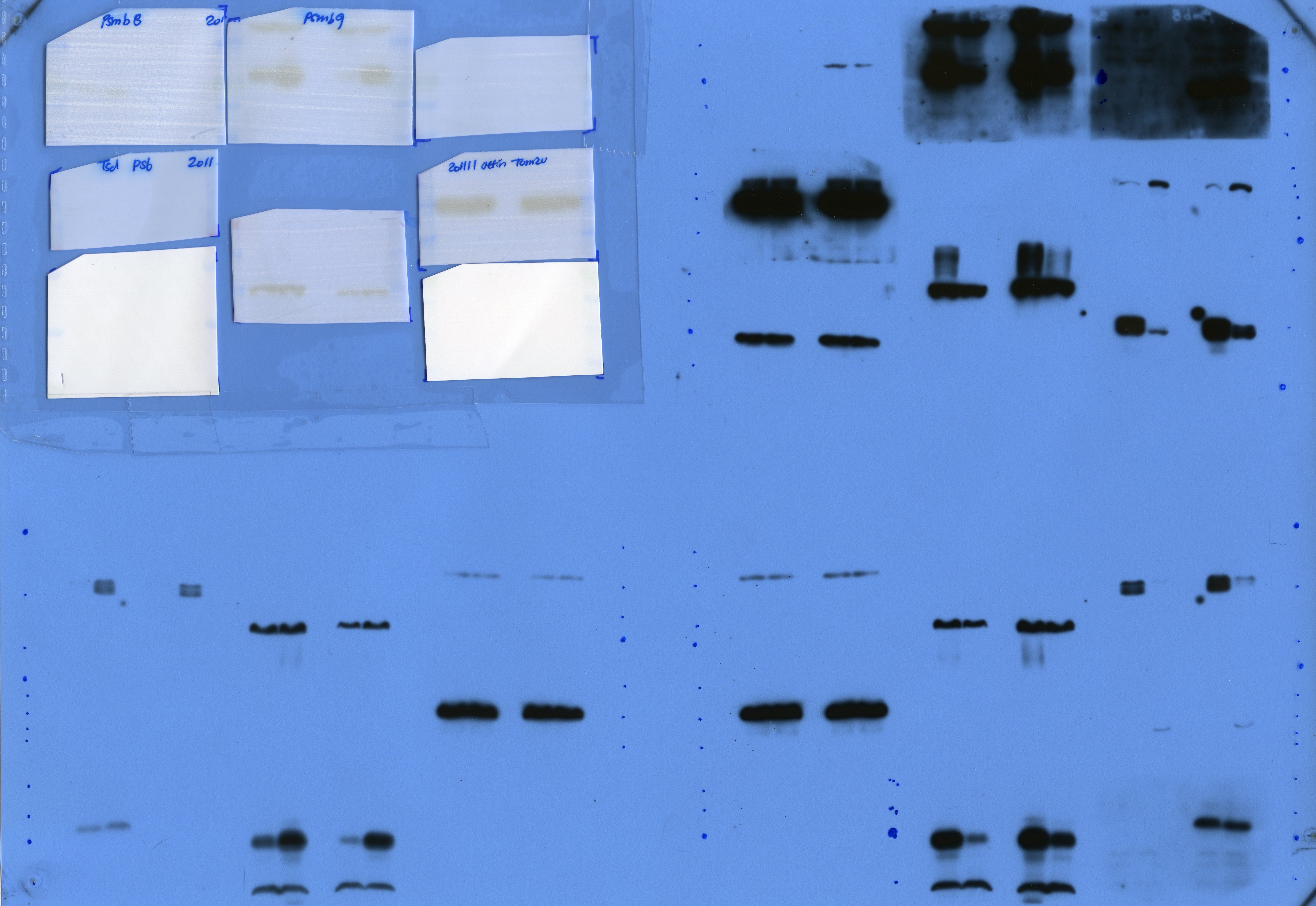

Supplement: Source data 5. [file elife-70079-supp5.zip › Figure S10B_psmb10-Tsc1-pS6.jpg]

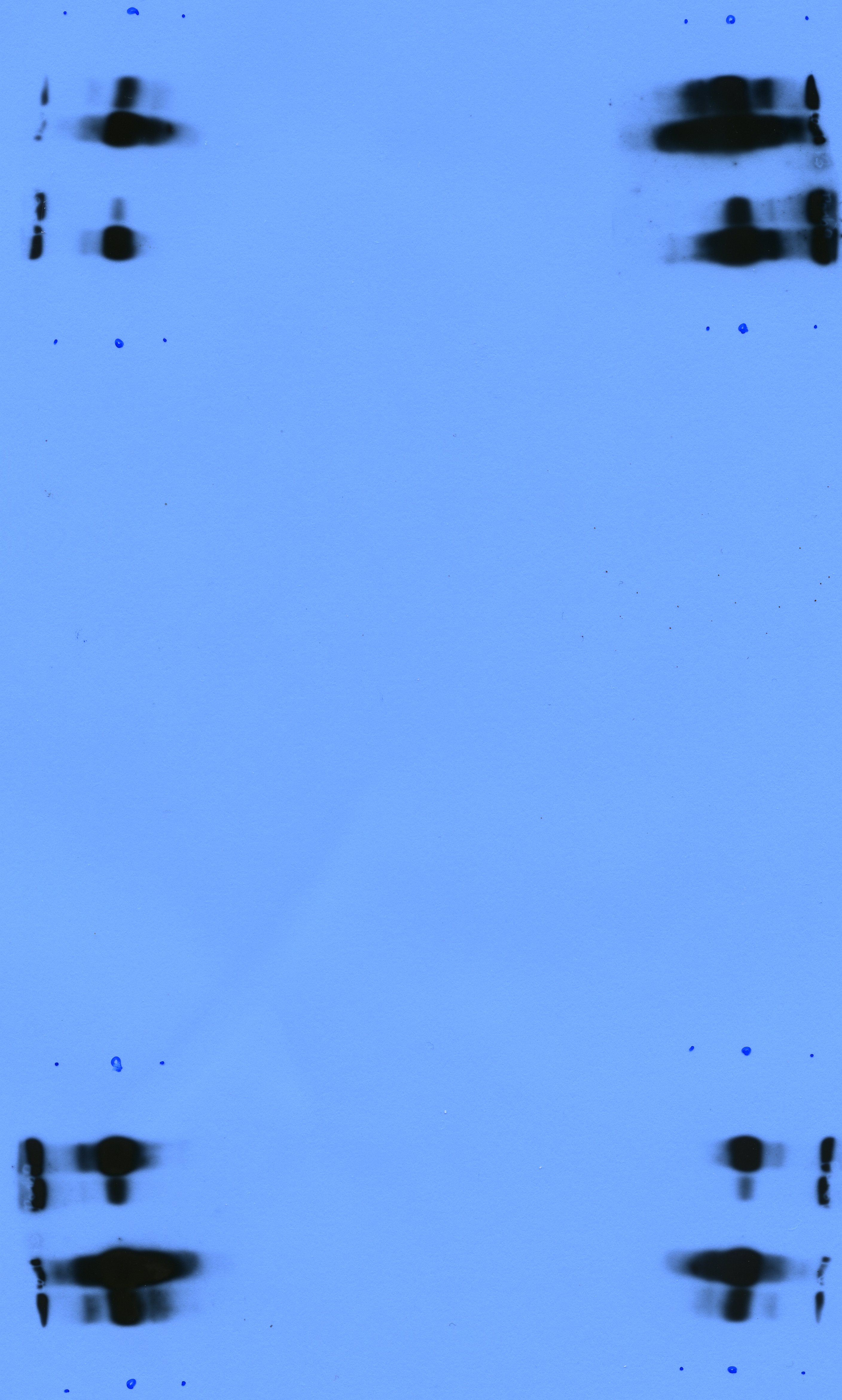

Supplement: Source data 5. [file elife-70079-supp5.zip › Figure S10B_psmb9.jpg]
